# Supplementary material for: Evaluating sickness absence duration by musculoskeletal and mental health issues: a retrospective cohort study of Scottish healthcare workers
Source: BMJ Open. 2018 Jan 26;8(1):e018085. doi: 10.1136/bmjopen-2017-018085 (PMC5829784; doi:10.1136/bmjopen-2017-018085)
Supplement: Supplementary file 1 [file bmjopen-2017-018085supp001.pdf]

Evaluating Sickness Absence Duration by Musculoskeletal and Mental Health Issues. A retrospective cohort study of Scottish Healthcare Workers

[Supplementary material]

Evangelia Demou PhD[1], Shanley Smith[2], Abita Bhaskar[1], Daniel F Mackay[2], Judith Brown PhD[2], Kate Hunt[1], Sergio Vargas-Prada MD[3,4], Ewan B Macdonald MD[2]

1. MRC/CSO Social and Public Health Sciences Unit, University of Glasgow, Glasgow, G2 3QB, United Kingdom

2. Healthy Working Lives Group, University of Glasgow, Glasgow, G12 8RZ, United Kingdom

3. Center for Research in Occupational Health (CiSAL), Universitat Pompeu Fabra, Barcelona, Spain

4. Unidad Central de Contingencias Comunes (U3C), ASEPEYO, Barcelona, Spain.

Running title Sickness Absence Duration in Healthcare Workers

Corresponding Author

Dr Evangelia Demou

Research Fellow

MRC/CSO Social and Public Health Sciences Unit

University of Glasgow

200 Renfield Street, Glasgow, G2 3QB, UK

Tel: 0141 353 7580

Figure S1a. Mean duration (in days) of absence by cause of sickness

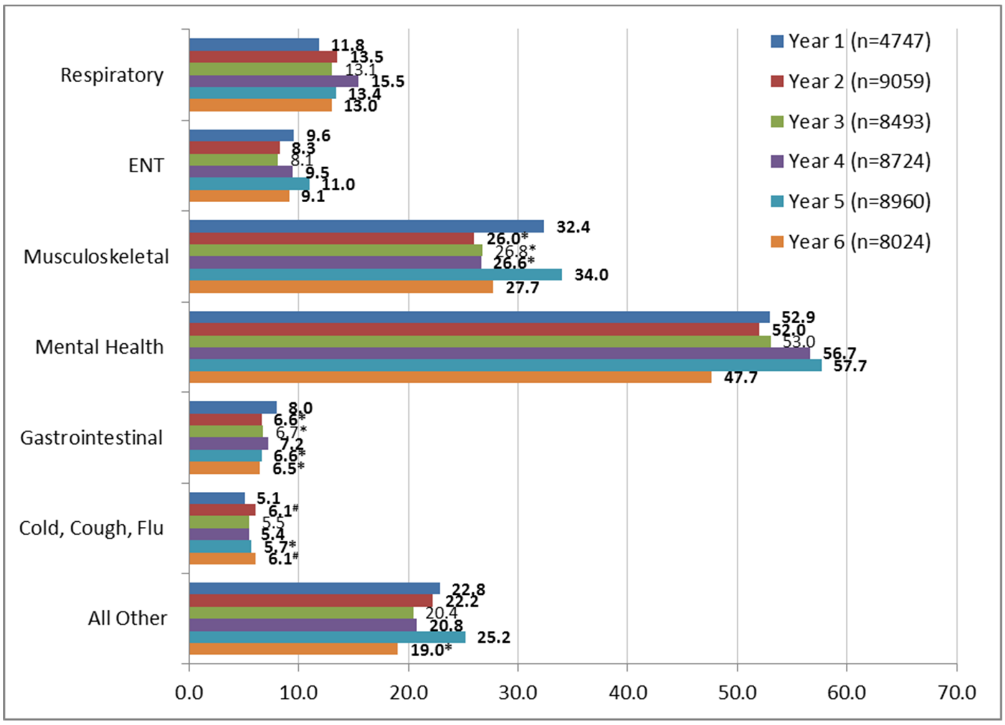

Figure S1b. Mean duration (in days) of absence by MSK cause of sickness

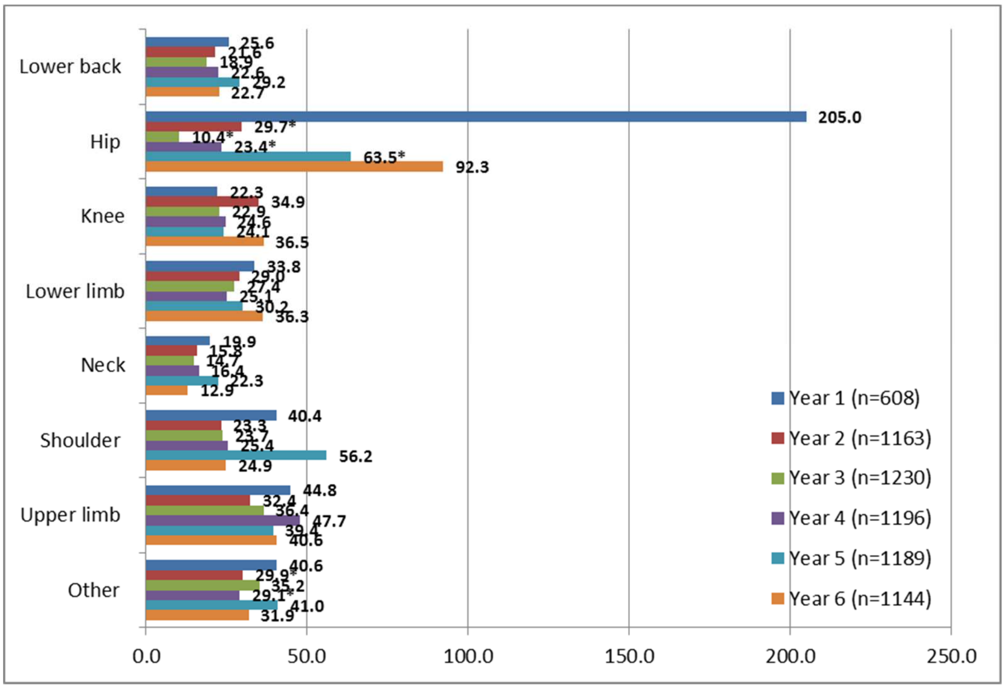

Figure S1c. Mean duration (in days) of absence by MH cause of sickness

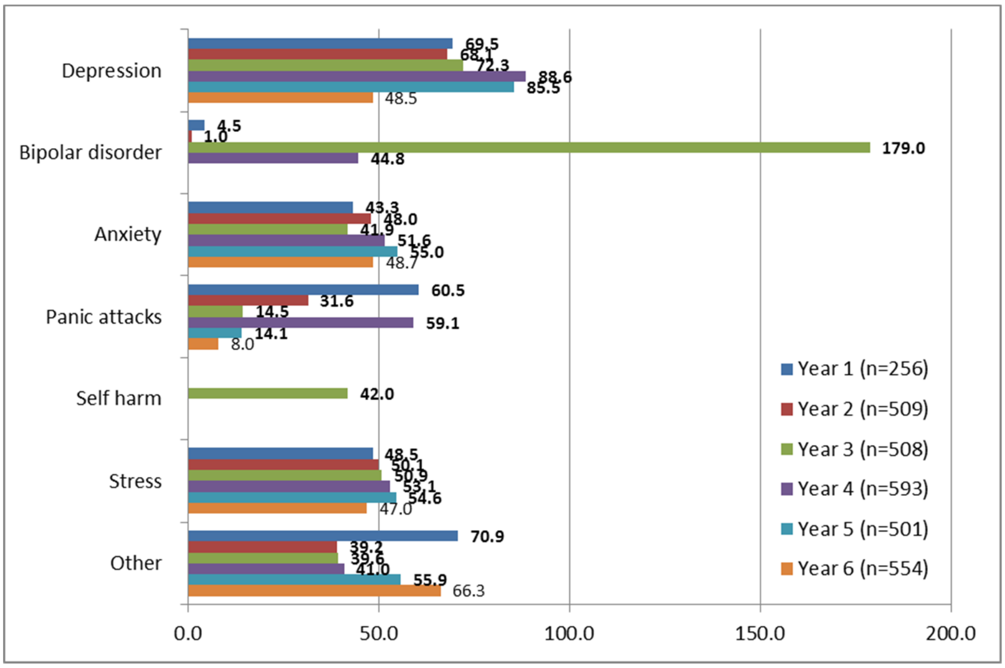

Table S1. Multivariate cox regression RTW hazard ratios for all (minus MSK and MH), MSK and MH conditions* with results from the Proportional Hazards test.

	All Conditions (minus MSK & MH)				Musculoskeletal Conditions				Mental Health Conditions			
	HR	95% CI	P	PH Test	HR	95% CI	P	PH Test	HR	95% CI	P	PH Test
**Population**												
All EASY except MSK & MH	1				-	-	-		-	-	-	
MSK	0.54	(0.53, 0.56)	0.000	0.0000	-	-	-		-	-	-	
MH	0.54	(0.33, 0.35)	0.000	0.0000	-	-	-		-	-	-	
**MSK condition**												
Lower back	-	-	-		1				-	-	-	
Knee	-	-	-		0.85	(0.75, 0.96)	0.008	0.0142	-	-	-	
Lower limb	-	-	-		0.80	(0.73, 0.88)	0.000	0.0005	-	-	-	
Neck	-	-	-		1.20	(1.08, 1.34)	0.001	0.5913	-	-	-	
Shoulder	-	-	-		0.80	(0.71, 0.91)	0.000	0.0013	-	-	-	
Upper limb	-	-	-		0.63	(0.59, 0.69)	0.000	0.0000	-	-	-	
Other	-	-	-		0.77	(0.72, 0.83)	0.000	0.0061	-	-	-	
**MH condition**												
Depression	-	-	-		-	-	-		1			
Anxiety	-	-	-		-	-	-		1.53	(1.33, 1.76)	0.000	0.2158
Stress	-	-	-		-	-	-		1.46	(1.30, 1.64)	0.000	0.0857
Other	-	-	-		-	-	-		1.51	(1.24, 1.85)	0.000	0.0004
**Gender**												
Male	1				1		1		1			
Female	0.91	(0.87, 0.95)	0.000	0.0000	0.81	(0.74, 0.88)	0.81	0.0004	1.06	(0.92, 1.23)	0.388	0.0702
**Age**	0.99	(0.99, 0.99)	0.000	0.0000	0.99	(0.99, 0.99)	0.99	0.8371	0.99	(0.99, 1.00)	0.000	0.4740
**Job category**												
Nursing/Midwifery	1				1				1			
Administrative services	1.19	(1.15, 1.23)	0.000	0.0059	1.18	(1.08, 1.28)	0.000	0.0025	0.97	(0.88, 1.08)	0.595	0.5969
Allied Health Profession	1.31	(1.26, 1.37)	0.000	0.0128	1.16	(1.05, 1.29)	0.004	0.3794	1.16	(1.00, 1.34)	0.043	0.7472
Healthcare Sciences	1.21	(1.14, 1.28)	0.000	0.3422	1.28	(1.09, 1.50)	0.003	0.0769	1.10	(0.87, 1.38)	0.437	0.3889

Manager	1.31	(1.04, 1.64)	0.020	0.0000	1.80	(1.04, 3.11)	0.036	0.7607	1.46	(0.79, 2.71)	0.229	0.7521
Medical & Dental	1.44	(1.33, 1.57)	0.000	0.0000	1.24	(0.97, 1.57)	0.080	0.0001	1.46	(0.90, 2.39)	0.127	0.0011
Medical and Dental Support	1.16	(1.06, 1.27)	0.001	0.4761	1.39	(1.06, 1.82)	0.018	0.9529	0.91	(0.62, 1.35)	0.644	0.7918
Other Therapeutic	1.17	(1.04, 1.31)	0.010	0.0017	1.44	(1.20, 1.75)	0.000	0.9414	1.22	(0.89, 1.67)	0.214	0.0001
Personal and Social Care	1.02	(0.97, 1.06)	0.458	0.7318	1.71	(1.28, 2.28)	0.000	0.8777	0.79	(0.51, 1.24)	0.308	0.7339
Support Services	1				1.10	(1.01, 1.19)	0.026	0.1622	1.21	(1.05, 1.40)	0.009	0.7192
**Job type**												
Part time	1				1				1			
Full time	1.12	(1.09, 1.14)	0.000	0.0003	1.04	(0.99, 1.11)	0.143	0.5504	1.09	(1.00, 1.18)	0.042	0.4597
**Year**												
May 08-Apr 09	1				1				1			
May 09-Apr 10	0.98	(0.95, 1.02)	0.417	0.0540	1.13	(1.02, 1.25)	0.020	0.9628	0.99	(0.85, 1.16)	0.91	0.9568
May 10-Apr 11	0.95	(0.92, 0.99)	0.013	0.0025	1.13	(1.03, 1.25)	0.014	0.7210	0.98	(0.84, 1.15)	0.822	0.2428
May 11-Apr 12	0.94	(0.90, 0.97)	0.001	0.0012	1.11	(1.00, 1.22)	0.045	0.6067	0.92	(0.79, 1.07)	0.279	0.5608
May 12-Apr 13	0.92	(0.88, 0.95)	0.000	0.0133	0.97	(0.87, 1.08)	0.560	0.3715	0.89	(0.77, 1.04)	0.154	0.3844
May 13-Apr 14	0.96	(0.92, 1.00)	0.029	0.0000	1.07	(0.96, 1.18)	0.217	0.3519	1.03	(0.88, 1.21)	0.687	0.9330

*results by Day of Absence and Season presented in Supplementary Material Tables S1a-f

Table S2a. Multivariate cox regression hazard ratios for full population, adjusted for sex, age, job category, job type, day of first absence, season of absence and year of absence.

All	HR	95% CI	P	PH Assumption
Day of absence				
Mon	1			
Tues	1.07	(1.04, 1.10)	0.000	0.5252
Wed	1.01	(0.98, 1.04)	0.384	0.3434
Thurs	1.01	(0.98, 1.04)	0.402	0.5309
Fri	0.96	(0.93, 0.99)	0.017	0.0021
Season				
Spring	1			
Summer	1.00	(0.97, 1.03)	0.931	0.0228
Autumn	1.00	(0.98, 1.03)	0.784	0.2458
Winter	1.02	(0.99, 1.05)	0.133	0.0905

Table S2b. Multivariate cox regression hazard ratios for full population, adjusted for sex, age, job category, job type, day of first absence, season of absence, year of absence and time varying coefficients.

All	HR	95% CI	P
Day of absence			
Mon	1		
Tues	1.06	(1.04, 1.09)	0.000
Wed	1.01	(0.98, 1.04)	0.612
Thurs	1.00	(0.97, 1.03)	0.976
Fri	0.94	(0.91, 0.98)	0.001
Season			
Spring	1		
Summer	1.00	(0.97, 1.03)	0.992
Autumn	1.01	(0.98, 1.03)	0.598
Winter	1.02	(0.99, 1.04)	0.163

Table S2c. Multivariate cox regression hazard ratios for staff with musculoskeletal conditions, adjusted for sex, age, job category, job type, day of first absence, season of absence and year of absence.

Musculoskeletal	HR	95% CI	P	PH Assumption
Day of absence				
Mon	1			
Tues	1.06	(0.99, 1.14)	0.105	0.2140
Wed	1.06	(0.99, 1.14)	0.1	0.5936
Thurs	1.08	(1.00, 1.17)	0.064	0.1939
Fri	1.00	(0.92, 1.09)	0.978	0.9834
Season				
Spring	1			
Summer	1.01	(0.94, 1.09)	0.694	0.7564
Autumn	1.01	(0.94, 1.08)	0.793	0.3528
Winter	1.02	(0.95, 1.09)	0.641	0.4783

Table S2d. Multivariate cox regression hazard ratios for staff with musculoskeletal conditions, adjusted for sex, age, job category, job type, day of first absence, season of absence, year of absence and time varying coefficients.

Musculoskeletal	HR	95% CI	P
Day of absence			
Mon	1		
Tues	1.06	(0.99, 1.14)	0.116
Wed	1.06	(0.99, 1.15)	0.090
Thurs	1.08	(1.00, 1.17)	0.050
Fri	1.00	(0.92, 1.09)	0.996
Season			
Spring	1		
Summer	1.01	(0.94, 1.08)	0.824
Autumn	1.01	(0.94, 1.08)	0.798
Winter	1.01	(0.95, 1.08)	0.679

Table S2e. Multivariate cox regression hazard ratios for staff with mental health conditions, adjusted for sex, age, job category, job type, day of first absence, season of absence and year of absence.

Mental health	HR	95% CI	P	PH Assumption
Job type				
Part time	1			
Full time	1.030726	(0.93, 1.15)	0.579	0.3336
Day of absence	1.015467	(0.92, 1.13)	0.772	0.027
Mon	0.987987	(0.88, 1.11)	0.839	0.5043
Tues	0.915464	(0.81, 1.03)	0.139	0.0068
Wed				
Thurs	1			
Fri	1.039512	(0.93, 1.16)	0.496	0.3026
Season	1.056732	(0.96, 1.17)	0.275	0.4850
Spring	1.019983	(0.92, 1.13)	0.712	0.5526
Summer	1			
Autumn	1.030726	(0.93, 1.15)	0.579	0.3336
Winter	1.015467	(0.92, 1.13)	0.772	0.027

Table S2f. Multivariate cox regression hazard ratios for staff with mental health conditions, adjusted for sex, age, job category, job type, day of first absence, season of absence, year of absence and time varying coefficients.

Mental health	HR	95% CI	P
Job type			
Part time	1		
Full time	1.00	(0.90, 1.12)	0.956
Day of absence	0.96	(0.86, 1.08)	0.519
Mon	0.91	(0.79, 1.05)	0.188
Tues	0.82	(0.70, 0.96)	0.011
Wed			
Thurs	1		
Fri	1.04	(0.93, 1.17)	0.448
Season	1.06	(0.96, .17)	0.235
Spring	1.03	(0.92, 1.14)	0.638
Summer	1		
Autumn	1.00	(0.90, 1.12)	0.956
Winter	0.96	(0.86, 1.08)	0.519

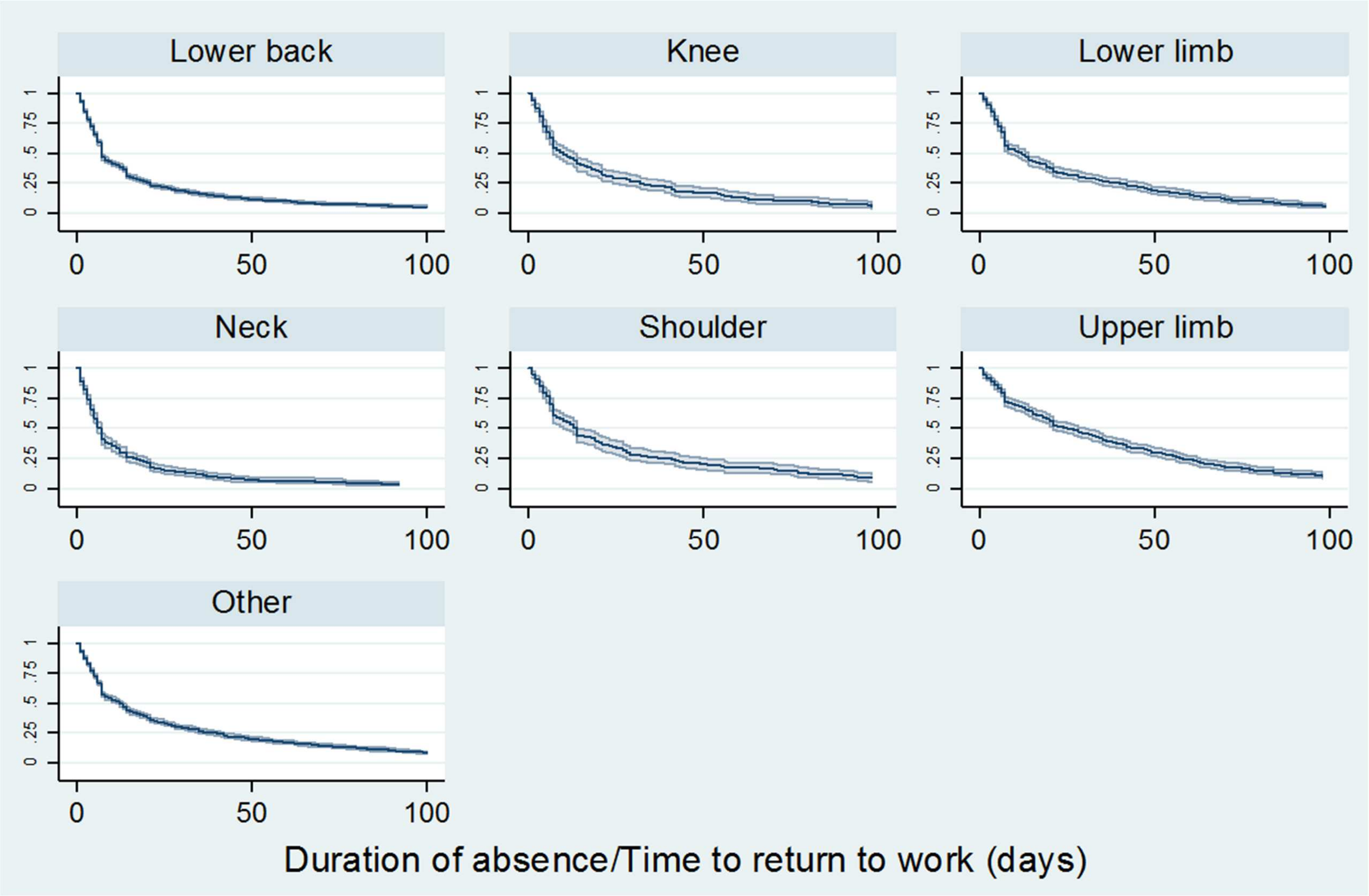

Figure S2. Return to Work curves for all MSK-related absences by MSK condition with 95%CI

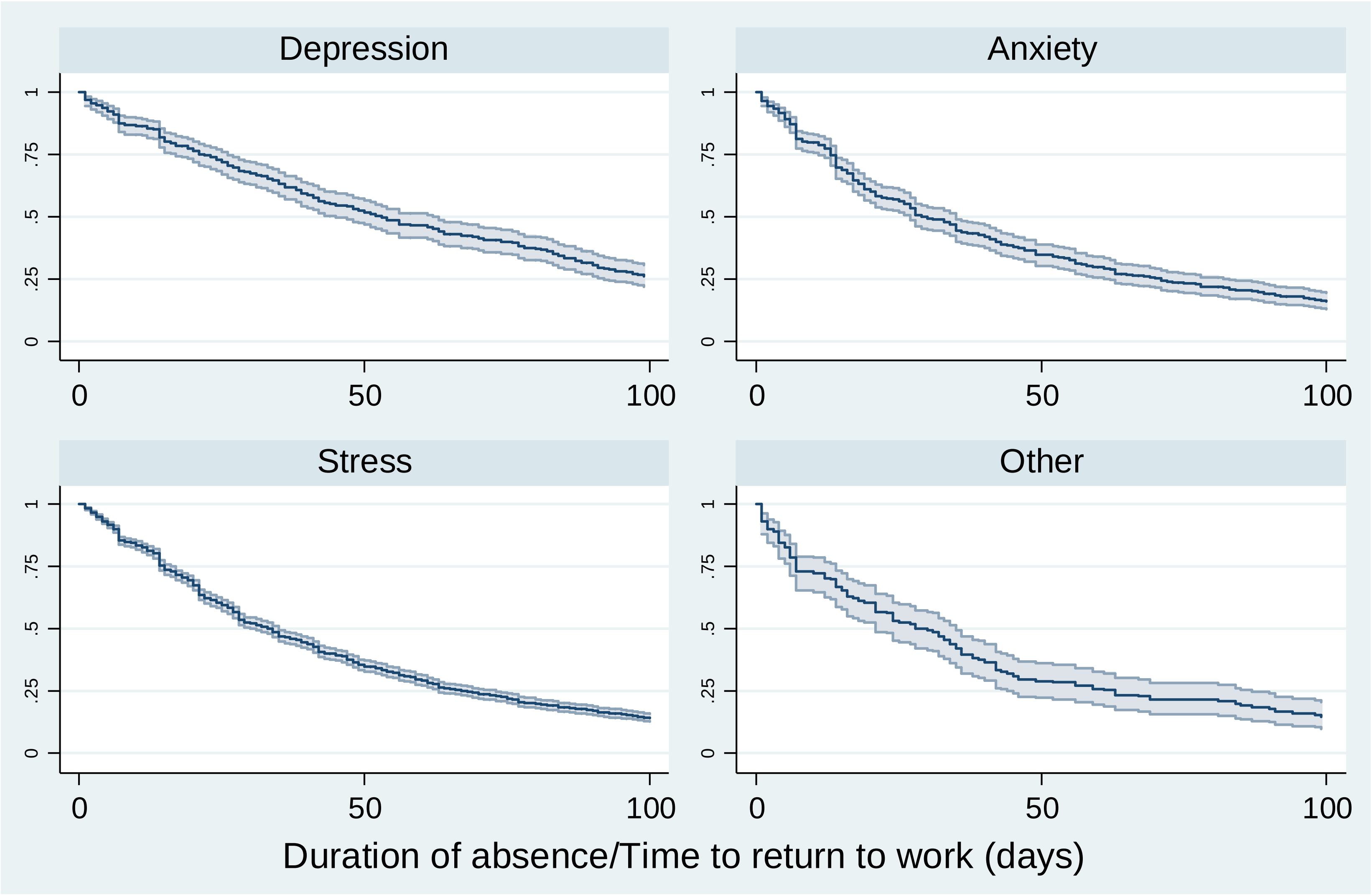

Figure S3. Return to Work curves for all MH-related absences by MH condition with 95%CI
