# Supplementary material for: Evaluating sickness absence duration by musculoskeletal and mental health issues: a retrospective cohort study of Scottish healthcare workers
Source: BMJ Open. 2018 Jan 26;8(1):e018085. doi: 10.1136/bmjopen-2017-018085 (PMC5829784; doi:10.1136/bmjopen-2017-018085)
Supplement: Author's manuscript [file bmjopen-2017-018085.draft_revisions.pdf]

BMJ Open is committed to open peer review. As part of this commitment we make the peer review history of every article we publish publicly available.

When an article is published we post the peer reviewers' comments and the authors' responses online. We also post the versions of the paper that were used during peer review. These are the versions that the peer review comments apply to.

The versions of the paper that follow are the versions that were submitted during the peer review process. They are not the versions of record or the final published versions. They should not be cited or distributed as the published version of this manuscript.

BMJ Open is an open access journal and the full, final, typeset and author-corrected version of record of the manuscript is available on our site with no access controls, subscription charges or pay-per-view fees (http://bmjopen.bmj.com).

If you have any questions on BMJ Open's open peer review process please email editorial.bmjopen@bmj.com

BMJ Open

Sickness Absence Duration in Healthcare Workers: The Impact of Musculoskeletal and Mental Health Problems

Journal:	*BMJ Open*
Manuscript ID	bmjopen-2017-018085
Article Type:	Research
Date Submitted by the Author:	05-Jun-2017
Complete List of Authors:	Demou, Evangelia; University of Glasgow, MRC/CSO Social and Public Health Sciences Unit; University of Glasgow, Public Health, Institute of Health and Wellbeing<br>Smith, Shanley; University of Glasgow, Public Health, Institute of Health and Wellbeing<br>Bhaskar, Abita; University of Glasgow, MRC/CSO Social and Public Health Sciences Unit<br>Brown, Judith; University of Glasgow, Public Health, Institute of health and Wellbeing<br>Hunt, Kate; University of Glasgow, MRC/CSO Social and Public Health Sciences Unit<br>Vargas, Sergio; Pompeu Fabra University, Center for Research in Occupational Health (CiSAL)<br>Macdonald, Ewan; The University of Glasgow, Healthy working lives group
**Primary Subject Heading**:	Occupational and environmental medicine
Secondary Subject Heading:	Epidemiology, Health policy, Mental health, Occupational and environmental medicine, Public health
Keywords:	MENTAL HEALTH, healthcare worker, anxiety, depression, Musculoskeletal disorders < ORTHOPAEDIC & TRAUMA SURGERY, return to work

SCHOLARONE™
Manuscripts

Sickness Absence Duration in Healthcare Workers: The Impact of Musculoskeletal and Mental Health Problems

Evangelia Demou PhD[1], Shanley Smith[2], Abita Bhaskar[1], Judith Brown PhD[2], Kate Hunt PhD[1], Sergio Vargas-Prada MD PhD[3,4,5,6], Ewan B Macdonald MD[2]

1. MRC/CSO Social and Public Health Sciences Unit, University of Glasgow, Glasgow, G2 3QB, United Kingdom

2. Public Health, Institute of Health and Wellbeing, University of Glasgow, Glasgow, G12 8RZ, United Kingdom

3. Center for Research in Occupational Health (CiSAL), Universitat Pompeu Fabra, Barcelona, Spain

4. Unidad Central de Contingencias Comunes (U3C), ASEPEYO, Barcelona, Spain.

5. CIBER Epidemiología y Salud Pública (CIBERESP), Spain.

6. IMIM (Hospital del Mar Research Institute), Barcelona, Spain

Running title Sickness Absence Duration in Healthcare Workers

Word count: 4,025

Number of figures: 2

Number of tables: 2

Key terms work; return to work; nurses; doctors, back pain, depression, anxiety, healthcare worker

Corresponding Author
Dr Evangelia Demou
Research Fellow
MRC/CSO Social and Public Health Sciences Unit
University of Glasgow
200 Renfield Street, Glasgow, G2 3QB, UK
Email: evangelia.demou@glasgow.ac.uk
Tel: 0141 353 7580

For peer review only

For peer review only - http://bmjopen.bmj.com/site/about/guidelines.xhtml

Abstract

Objectives: Sickness absence (SA) among healthcare workers is associated with occupational and non-occupational risk factors, and impacts employee health, healthcare delivery and patient health. Musculoskeletal (MSK) and mental health (MH) issues are leading causes of SA, but there is a lack of research on the impact of specific MSK/MH conditions on SA duration. The study aim is to determine differences in SA duration by MH and MSK disorders in healthcare employees.

Methods: Survival analyses and Cox's proportional hazards models were used to estimate SA duration due to MSK and MH problems over six years, using a health board bespoke database. SA duration and time to return-to-work were estimated for employees by age, gender, job and health conditions.

Results: MSK and MH conditions accounted for 27% and 6%, respectively, of all SA events and 23.7% and 19.5% of all days lost. Average SA duration over the study period was 43.5 days for MSK and 53.9 days for MH conditions. For MSK conditions, employees with low back pain had the fastest return-to-work (P_{50}: 7 days), while employees absent due to depression took the longest (P_{50}: 54 days). The most influential socio-demographic variables affecting return-to-work were age, gender, and job category.

Conclusions: This cross-sectional study used a unique and rich database to quantify SA duration by specific cause for MSK and MH-related SA events. Our findings can be used by public health practitioners and healthcare managers to develop and implement tailored and targeted workplace interventions for employees with MSK and MH problems.

For peer review only - http://bmjopen.bmj.com/site/about/guidelines.xhtml

Article summary - Strengths and limitations of this study

- Healthcare is one of the sectors with significantly higher rates of sickness absence (SA), impacting on employee health, healthcare delivery and patient health.
- Survival analyses and Cox's proportional hazards models were used to estimate SA duration due to musculoskeletal (MSK) and mental health (MH) problems over six years (N=48,007 SA events).
- SA duration and time to return-to-work were estimated for employees by age, gender, job and health conditions.
- SA data are from a single large health board (c.12,000 employees), with a SA rate similar to the Scottish NHS average.
- Understanding the impact of socio-demographic, health and occupational factors on RTW times can inform the development of tailored SA management interventions.

For peer review only

For peer review only - http://bmjopen.bmj.com/site/about/guidelines.xhtml

Introduction

Sickness absence (SA) is a significant public health burden on government, employees, employers, and public resources [1-3]. This is due to loss of productivity, increased workload on other staff, as well as resources spent to cover incapacity to work and restore health [2]. SA data is increasingly being used as a measure of ill-health [4]. The public health burden of sickness absence is recognized and governments and employers are developing policies toward providing support to employers and employees in improving health, reducing sickness absence rates, and improving return to work (RTW) times [5, 6].

Multiple factors need to be considered to effectively manage SA through policy and practice [7]. Sickness absence rates vary by sector and employer, with the healthcare sector exhibiting one of the highest rates[[8]. Additionally, within the health service, variations in SA rates depend on region, job category and salary grade, among other factors [3, 9, 10]. Cause of SA can have a major impact on duration and overall costs. Long term sickness absence (LTSA), although only responsible for a small percentage of SA *events*, makes up approximately 75% of absence *costs*, with musculoskeletal (MSK) and mental health (MH) disorders being two of the leading causes of LTSA [2, 3, 8, 11, 12].

<u>Healthcare Employees and Sickness Absence</u>

The healthcare sector is a diverse entity, which presents a unique set of challenges in relation to SA duration [13]. Studies show that healthcare workers experience high exposures to both occupational and non-occupational risk factors, negatively impacting on SA events [13-18]. Healthcare employee absence leads to increases in the risks to quality of patient care, workload stress of colleagues, and

For peer review only

For peer review only - http://bmjopen.bmj.com/site/about/guidelines.xhtml

employers' staffing costs [13, 19-22]. A study examining quality of care and treatment in 14 English hospital trusts found a positive association between the in-patient to staff ratio and high hospital standardized mortality ratio (HSMR) scores [23] and also identified insufficient nursing establishments and poor staffing levels on night shifts and weekends, partially due to high SA rates [23].

Impact of Sickness Absence Cause and Duration

Certain health conditions result in longer periods of SA [8]. These thus have large potential interest as the focus of workplace interventions, due to the greater impact longer SA spells have on individuals and employers [24, 25]. There are potential long term effects on individuals who experience particular types of SA events; one Swedish cohort study, for example, found an association between long term absence and lower disposable income in the 2-6 years following the absence event [26]. A previous study investigating the effectiveness of an early SA management service in a Scottish Health Board, found that although the leading causes of SA were gastrointestinal problems, followed by cold/cough/flu, the greatest impact on total number of days lost were due to musculoskeletal (MSK) and mental health (MH) problems [12].

MSK related absence

Health Response UK reports musculoskeletal injury as the leading cause of absenteeism and related cost to UK Industry, with back pain as one of the most common causes of MSK-related sickness absence [11]. Back pain is often recurrent, and the majority of people who have activity-limiting back pain go on to have recurrent SA episodes [27]. A systematic review examining SA and return-to-work (RTW), found

For peer review only

For peer review only - http://bmjopen.bmj.com/site/about/guidelines.xhtml

that approximately 20% of employees with back pain have some sickness absence episodes in the six to 12 month period following the back pain episode. RTW in this review was estimated at up to one month for 68% of individuals, 85% of individuals were back to work 1–6 months and 93% were back to work at 6 months post the SA event [28]. Although RTW rates for back pain tend to be high, the recurrent nature of back pain increases the risk for substantial overall impact on work days lost through repeated periods of SA.

Neck problems are also a common MSK condition resulting in lost working days [11]. One cohort study investigating the relationship between physical and psychosocial features of the workplace and SA, found that work activities involving neck flexion and neck rotation, high job demands, low skill discretion, and low job security were significantly associated with SA due to neck pain [29].

Mental Health related absence

A number of systematic reviews highlight the importance of recognizing mental health issues in the workplace to assist in the reduction of associated SA [30, 31]. However, there is limited evidence on how specific mental health conditions affect RTW times.

Depression is a leading cause of MH-related absence [8]. The extent and severity of depressive symptoms, comorbidity of anxiety, social and emotional support, education, and long symptom periods prior to diagnosis can affect the course of depression and RTW times [32-34].

For those experiencing anxiety that results in SA, previous anxiety episodes, older age, education, and long durations of untreated and undiagnosed symptoms, contribute to longer absences [34].

Detailed return-to-work figures following LTSA are scarce. Estimates suggest that as few as 13% of people who have experienced depression and anxiety are in employment, compared to 33% of people

For peer review only

For peer review only - http://bmjopen.bmj.com/site/about/guidelines.xhtml

experiencing other chronic illnesses [35].

Socio-demographic and Occupational Factors and Sickness Absence

Several studies have investigated the associations between sickness absence and an employee's socio-demographic and socio-economic characteristics [13, 36-40]. Evidence shows that sickness absence is multi-causal, and that in addition to providing support for an individual's specific characteristics and incapacitating condition, it is necessary to also consider an individual's work and workplace environment in order to effectively manage and improve return to work times [41, 42].

Aim

Despite a great deal of research recognizing the prevalence of MSK and MH issues and their impact on SA rates [3, 13-18]; there remains a lack of information on how specific types of musculoskeletal and mental health conditions may affect SA duration, which is vital to inform and improve current and new SA interventions, as well as support workplace modifications for SA prevention. This study aims to examine the impact of specific musculoskeletal and mental health conditions on sickness absence duration in healthcare workers, using a bespoke database [12, 43] over a six year period, and to assess how health, socio-demographic factors, work characteristics and occupational factors affect SA duration.

Methods

Study population

For peer review only

The study population includes all participants in the EASY (Early Access to Support for You) SA management service of a Scottish health board [12, 43]. EASY is a telephone-based service that provides early intervention (from Day 1 of absence) based on the biopsychosocial model [43, 44] . Data on all staff who engage with EASY (compliance rate was c.80%) are routinely entered into a bespoke database, including age, gender, job family, cause of absence, date of first day of absence, and return to work date [12, 43]. Detailed descriptions of the EASY service can be found elsewhere [12, 43]. We examined data on 66 490 unique absence events recorded in the EASY database between May 2008 and December 2014.

Defining and Recoding Variables

Sickness absence duration was calculated from the difference between the first date of absence and the RTW date. Cause of absence was grouped into eight categories: Respiratory, Musculoskeletal, Mental Health, Gastrointestinal, ENT, Cough/Cold/Flu, and All Other. Conditions assigned to the musculoskeletal category were grouped into nine subgroups according to anatomical site: Hip, Knee, Low Back, Lower Limb, Neck, Shoulder, Upper Limb, and All Other. Mental health conditions were allocated to eight subgroups according to diagnostic group: Bipolar Disorder, Anxiety, Depression, Schizophrenia, Panic attacks, Self-Harm, Stress, and All Other Psychiatric Disorders.

After missing data were eliminated (<0.05%), four main exclusion criteria were applied: (a) if there was no first day of absence (FDA) (N=196); (b) if the FDA was a Saturday or Sunday (N=4881), as there was no SA service on those days and absences would not be recorded on FDA; (c) if the 'date opened' (i.e. the date the EASY service contacted the absentee) was before the first day of absence (N=828); (d) if the 'date opened' was equal to or after the RTW date (N=3 465). Due to overlaps among the missing data

For peer review only - http://bmjopen.bmj.com/site/about/guidelines.xhtml

and exclusion criteria, a total of 13 286 absences were excluded; hence analysis was based on 53 193 unique absence events.

Analytic Strategy

Descriptive Statistics

Descriptive statistics and crosstabs were produced for the entire EASY population, which included all causes of sickness absence (N=53 193). Descriptive statistics and crosstabs were then produced for two specific causes, MH (N=3 093) and MSK (N=6 969) conditions.

Mean absence duration was calculated for the entire EASY population in the study, the entire EASY population without MSK and MH (N=43 131), MSK only, MH only, MSK sub categories, and MH sub categories for each year from May 2008 to December 2015. The data were divided into seven years as follows: May 2008–April 2009 (Year 1); May 2009–April 2010 (Year 2); May 2010–April 2011 (Year 3); May 2011–April 2012 (Year 4); May 2012-April 2013 (Year 5); May 2013-April 2014 (Year 6); May 2014-December 2015 (Year 7). Absences commencing in Year 7 were not included as the year was incomplete and in numerous cases absence could still be ongoing.

Kaplan Meier Survival Analysis & Cox Proportional Hazards Model

Absence duration was analyzed using Kaplan Meier survival analyses and Cox's proportional hazards models to determine the hazard (risk) of absentees returning to work. The model takes into account each sickness absence event as well as individuals with multiple absence events by calculating cluster

For peer review only

robust standard errors and the multivariate model controlled for several occupational and individual variables including: gender, age, job family, job type, cause of absence, day of absence, season of absence, and year of absence (Table S1).

All statistical analyses were conducted using STATA version 12.0 and R version 3.1.1.

Results

Descriptive statistics for the entire EASY population (May08-Dec14; N=48,007) are reported in **Table 1**. Almost 9 out of 10 (87.9%) of the EASY population were females (N=42,187). Over the six year period investigated, the three most common causes of SA events are gastrointestinal (N=13,459), cold, cough, and flu (N=8,657), and musculoskeletal (N=6,530) problems. Almost half the EASY population (45.3%) is in the nursing/midwifery job category, and a further 20% in administrative services. Numbers in part-time (43.2%) and full-time employment (56.8%) were relatively similar.

Fewer of those with MSK-related absences (N=6,530) were in the two youngest age groups, than in the total population, and the proportion of those with MSK absences who were in the Nursing/midwifery (N=3,428) job category (52.5%) was even higher than seen in the total population (45.3%). In the MH population, 91.8% are female, compared to 87.9% of the total population (Table 1).

For peer review only

For peer review only - http://bmjopen.bmj.com/site/about/guidelines.xhtml

Table 1. Descriptive statistics for total EASY population, and MSK and MH subgroups

		Total Easy Population (N=48,007)		Total MSK Population (N=6,530)		Total MH Population (N=2,921)	
		N	%	N	%	N	%
Age Group	16-29	6,885	14.34	588	9.00	253	8.66
	30-39	10,772	22.44	1,124	17.21	639	21.88
	40-49	15,257	31.78	2,291	35.08	1,102	37.73
	50-59	13,003	27.09	2,181	33.40	813	27.83
	60+	2,090	4.35	346	5.30	114	3.90
Gender	Male	5,820	12.12	963	14.75	241	8.25
	Female	42,187	87.88	5,567	85.25	2,680	91.75
Job Family	Administrative Services	9,597	19.99	1,003	15.36	550	18.83
	Allied Health Profession	4,914	10.24	502	7.69	208	7.12
	Healthcare Sciences	2,130	4.44	284	4.35	105	3.59
	Manager	150	0.31	13	0.20	5	0.17
	Medical & Dental	1,301	2.71	125	1.91	33	1.13
	Medical & Dental Support	876	1.82	83	1.27	35	1.20
	Nursing/Midwifery	21,734	45.27	3,428	52.50	1,614	55.26
	Other Therapeutic	2,100	4.37	193	2.96	74	2.53
	Personal and Social Care	443	0.92	56	0.86	18	0.62
	Support Services	4,762	9.92	843	12.91	279	9.55
Job Type	Part time	20,758	43.24	2,871	43.97	1,431	48.99
	Full time	27,249	56.76	3,659	56.03	1,490	51.01

For peer review only

For peer review only - http://bmjopen.bmj.com/site/about/guidelines.xhtml

Mean duration (in days) of absence by cause of sickness

Gastrointestinal (GI) and cold, cough, and flu (CCF) problems account for the largest number of sickness absence events, 28% and 18%, respectively. However, Figure 1a shows that the impact these causes have on total number of working days lost is much less (11.8% for GI and 6.3% for CCF). The health conditions with the highest impact on total number of working days lost are musculoskeletal (24%) and mental health (20%) conditions. Mean absence duration ranged from 5.6 days for CCF to 53.3 days for MH-related absences. MSK absences had an overall mean duration of 28.9 days.

Insert Figure 1.

The three most common types of MSK problems in this population are low back pain (33.6%), lower limb (9%), and upper limb problems (9%). Figure 1b shows that, within the subgroup with absences due to MSK, low back pain had the highest percent impact on total number of working days lost (26.8%), followed by upper limb problems (12.3%), and lower limb problems (9.1%). Mean absence duration within the MSK-related absences ranged from 17 days due to neck problems to 40 days for upper limb. All other MSK conditions did not differ greatly in duration, and ranged from 23.4 to 34.1 days (Figure S1b).

The three most common types of MH problems amongst those in the EASY population are stress (64.8%), anxiety (15.9%), and depression (13.7%). The job categories with the highest cases of MH-related absences are nursing/midwifery (55.3%), and administrative services (18.8%). Figure 1c demonstrates that stress accounts for the largest percent of working days lost (62.7%), followed by depression (18.9%) and anxiety (14.6%). There was no significant change year on year in mean duration of MH-related absences (Figure S1c), with depression resulting in the longest absences in all years

For peer review only - http://bmjopen.bmj.com/site/about/guidelines.xhtml

(overall mean duration of 72.1 days). Anxiety and stress related absences had a mean duration of 48.1 and 50.7 days, respectively.

Mean absence duration in years two, three, four, five, and six of the EASY service was compared to year one using linear regression for all causes of SA to examine potential significant changes over time, and then for each of the nine conditions (Figure S1). Compared to year one, mean absence duration for MSK cases in Year 2, Year 3 and Year 4 decreased significantly (Figure S1).; mean absence duration for gastrointestinal (GI) cases in years five and six decreased significantly; and mean absence duration for cold, cough, and flu cases in years two, five and six decreased significantly (Figure S1c).

<u>Sickness absence duration & return to work</u>

Figure 2 shows the Kaplan Meier RTW curves for all sickness absence events minus MSK and MH related absences (Figure 2a), for the MSK-related absences (Figure 2b) and for the MH-related absences (Figure 2c). RTW for staff absent because of MH problems was much longer than all other causes of absences (Figure 2a). For example, 50% for staff absent from work due to a MH problem had returned to work by 35 days, whereas 50% of those with an absence due to an MSK condition or all other conditions had returned within 10 and 5 days (respectively) of their FDA. As shown in Figure 2b, there are significant differences in RTW duration by sub-conditions within absences due to a MSK condition. Upper limb conditions result in the longest absences (50% of staff RTW by 25 days) whereas lower back and neck problems, result in the shortest absences (50% of staff RTW by 7 days for both conditions). For the other musculoskeletal conditions (knee, lower limb, shoulder and other), 50% of the population RTW (P_{50}) between 10 and 14 days. Mental health related absences are much longer (**Figure 2c**). Depression is the leading cause of longer SA events, with 50% of staff RTW by 53 days, followed by stress (50% of staff

For peer review only - http://bmjopen.bmj.com/site/about/guidelines.xhtml

RTW by 34 days) and anxiety and Other mental health conditions (50% of staff RTW by 29 days).

Table 2. Multivariate cox regression RTW hazard ratios for all (minus MSK and MH), MSK and MH conditions*.

	**All Conditions (minus MSK & MH)**			**Musculoskeletal Conditions**			**Mental Health Conditions**		
	**HR**	**95% CI**	**P**	**HR**	**95% CI**	**P**	**HR**	**95% CI**	**P**
**Population**									
All EASY except MSK & MH	1			-	-	-	-	-	-
MSK	0.51	(0.49, 0.52)	0.000	-	-	-	-	-	-
MH	0.24	(0.23, 0.26)	0.000	-	-	-	-	-	-
**MSK-specific**									
Lower back	-	-	-	1			-	-	-
Knee	-	-	-	0.84	(0.75, 0.95)	0.006	-	-	-
Lower limb	-	-	-	0.80	(0.73, 0.88)	0.000	-	-	-
Neck	-	-	-	1.16	(1.05, 1.29)	0.003	-	-	-
Shoulder	-	-	-	0.79	(0.69, 0.89)	0.000	-	-	-
Upper limb	-	-	-	0.61	(0.56, 0.68)	0.000	-	-	-
Other	-	-	-	0.73	(0.68, 0.78)	0.000	-	-	-
**MH-specific**									
Depression	-	-	-	-	-	-	1		
Anxiety	-	-	-	-	-	-	1.63	(1.40, 1.89)	0.000
Stress	-	-	-	-	-	-	1.64	(1.41, 1.90)	0.000
Other	-	-	-	-	-	-	1.80	(1.43, 2.27)	0.000
**Gender**									
Male	1			1			1		
Female	0.94	(0.89, 0.94)	0.000	0.77	(0.71, 0.84)	0.000	1.07	(0.93, 1.23)	0.336
**Age**	0.99	(0.99, 0.99)	0.000	0.99	(0.99, 0.99)	0.000	0.99	(0.99, 1.00)	0.000
**Job category**									
Administrative services	1			1			1		
Allied Health Profession	1.10	(1.06, 1.13)	0.000	0.99	(0.88, 1.10)	0.797	1.18	(1.00, 1.38)	0.045
Healthcare Sciences	1.01	(0.96, 1.06)	0.642	1.09	(0.96, 1.25)	0.197	1.11	(0.90, 1.37)	0.330
Manager	1.11	(0.95, 1.31)	0.195	1.49	(0.86, 2.58)	0.156	1.46	(0.60, 3.55)	0.399
Medical & Dental	1.17	(1.11, 1.25)	0.000	0.99	(0.82, 1.20)	0.921	1.43	(1.00, 2.05)	0.048
Medical and Dental Support	0.96	(0.90, 1.03)	0.292	1.18	(0.95, 1.49)	0.141	0.98	(0.69, 1.38)	0.892
Nursing/Midwifery	0.84	(0.82, 0.86)	0.000	0.85	(0.79, 0.91)	0.000	1.01	(0.91, 1.11)	0.918
Other Therapeutic	1.23	(1.17, 1.29)	0.000	1.24	(1.06, 1.45)	0.007	1.21	(0.95, 1.55)	0.124
Personal and Social Care	0.95	(0.86, 1.05)	0.290	1.41	(1.08, 1.85)	0.013	0.84	(0.53, 1.36)	0.485

For peer review only - http://bmjopen.bmj.com/site/about/guidelines.xhtml

Support Services	0.85	(0.82, 0.89)	0.000	0.93	(0.84, 1.02)	0.129	1.23	(1.06, 1.42)	0.007
**Job type**									
Part time	1			1			1		
Full time	1.12	(1.10, 1.15)	0.000	1.05	(0.99, 1.10)	0.090	1.08	(1.00, 1.17)	0.048
**Year**									
May 08-Apr 09	1			1			1		
May 09-Apr 10	0.99	(0.95, 1.02)	0.466	1.13	(1.02, 1.24)	0.020	1.00	(0.86, 1.17)	0.978
May 10-Apr 11	0.95	(0.92, 0.99)	0.012	1.13	(1.03, 1.25)	0.013	0.99	(0.85, 1.15)	0.884
May 11-Apr 12	0.94	(0.91, 0.98)	0.002	1.11	(1.00, 1.22)	0.043	0.92	(0.79, 1.07)	0.273
May 12-Apr 13	0.92	(0.89, 0.96)	0.000	0.98	(0.88, 1.08)	0.624	0.91	(0.78, 1.06)	0.232
May 13-Apr 14	0.96	(0.93, 1.00)	0.036	1.08	(0.97, 1.19)	0.144	1.04	(0.89, 1.21)	0.617

*results by Day of Absence and Season presented in Supplementary Material Tables S1a-c

For peer review only

Insert Figure 2.

For peer review only - http://bmjopen.bmj.com/site/about/guidelines.xhtml

Multivariate Analysis for all EASY absences (minus MSK & MH), MSK and MH

Table 2 shows the results of the multivariate cox regression analysis to investigate the differences in return to work between the three main absence groups, gender, job title and year of absence. In terms of absence cause, in comparison to all of EASY (minus MSK & MH), time to RTW was 49% (95% CI .49-.52, P<0.0001) and 76% (95% CI .23-.66, P<0.0001) longer for absences due to MSK and MH conditions, respectively. No real differences were seen after adjusting for gender, age, job family, job type, cause of absence, day of absence, season of absence, and year of absence.

For MSK-related absences, staff absent due to neck problems took 16% longer to return to work (95% CI 1.05-1.29, P<0.0001) compared to those absent because of lower back problems, whereas employees who were absent because of all other MSK conditions (0.73; CI: 0.68, 0.78) had quicker RTW.

Anxiety, stress and all other mental health related absences resulted in significantly longer times to return to work, when compared to depression, and were 63%, 64% and 80% of longer duration, respectively (all p<0.001).

The analysis by gender demonstrated that for all absences (minus MSK & MH) and for MSK absences women exhibited longer RTW times than men (Table 2). No significant gender difference in RTW was observed for mental health conditions. From the data, it is not possible to ascertain, whether full-time or part-time working has any association with RTW, as hours and days of employment were not included in the database.

In terms of job category, for all conditions (minus MSK & MH) nurses had the longer duration to RTW followed by staff in 'support services' when compared to staff in the reference category of

For peer review only - http://bmjopen.bmj.com/site/about/guidelines.xhtml

'administrative services', (Table 2). Employees in 'other therapeutic services' and 'medical and dental' staff demonstrated 23% and 17%, respectively, shorter times to RTW than staff in 'administrative services'. For MSK related absence, the only significant differences to the reference category 'administrative services' was observed for 'nursing/midwifery' (HR: 0.85; Cis: 0.79, 0.91; p=0.000), and the 'other therapeutic' and 'personal and social care' categories. For mental health related absences, only 'personal and social care' staff took significantly longer to RTW compared to 'administrative' staff, whereas 'medical and dental' staff and 'allied health professionals' had 43% and 18%, respectively, shorter lengths of absence RTW than the 'administrative' job group.

The analysis by year of absence using Year 1 as the reference category, showed that staff take significantly longer to return to work in years 3-6 for all conditions (minus MSK & MH). When only MSK absences are examined, in years 2-4 staff time to RTW is significantly shorter than for year 1. There are no significant differences in time to RTW by year for staff absences due to a mental health condition. Additionally, when examining time to RTW by day of absence start on absences starting on a Tuesday took significantly longer to RTW (Tables S1a-c). No significant differences in time to RTW were observed for season in which the absence occurred (p>0.05) (Tables S1a-c).

Discussion

<u>Summary of findings</u>

We found significant differences in sickness absence duration by presenting condition in a population of healthcare workers. Mental health conditions, and depression specifically, accounted for the most

For peer review only - http://bmjopen.bmj.com/site/about/guidelines.xhtml

working days' absence. We also observed significant variations in duration for different musculoskeletal conditions. Upper limb disorders resulted in the longest sickness absence durations among these healthcare workers, with 50% of staff returning to work by day 25. Lower back and neck problems resulted in the shortest MSK-related absences (50% RTW by 7 days), whereas knee, lower limb, shoulder and other MSK conditions resulted in SA durations that were comparable to each other (50% RTW between 10-14 days).

Employees within the nursing and midwifery job category account for almost half (45.3%) of all sickness absence events recorded in the six year period, and over half of the MSK (52.5%) and MH-related (55.3%) absence events. Nurses and midwives also had longer times to RTW than every other job category when compared to the reference category of 'administrative services'. In terms of gender, no significant differences were detected in RTW between men and women who were absent from work because of mental health conditions, but for all other absence events women took longer to return to work than men.

Research in context to previous studies

The results of our study are in agreement with previous published work on the impact of musculoskeletal and mental health conditions on sickness absence and return to work [2, 8, 45-47]. A Danish cohort study aiming to identify prognostic factors associated with neck-shoulder pain resulting in long term sickness absence, found pain intensity and job characteristics, such as heavy physical workload, were significantly associated with longer absence duration [48]. Armijo-Olivo et al. (2016) also demonstrated that occupation and health condition were significant factors, among others, in the

For peer review only - http://bmjopen.bmj.com/site/about/guidelines.xhtml

rehabilitation process of people affected by MSK conditions [49]. Similarly for mental health conditions, previous research suggests that sickness absence associated with psychological ill-health tends to be higher among NHS healthcare workers than for other employment sectors in the UK [50]. This may be due to the pressured nature of the work, constant organizational changes, and the large workload [51], supporting the notion that organizational factors may contribute to the level of psychological ill health experienced by staff. These findings are also reflected in our study, where significant variations in absence duration are observed not only by specific MSK and MH conditions, but also by job categories - with nursing and midwifery staff experiencing the majority of and longest SA events. One study looking at job family and sickness absence in the healthcare sector, found that doctors have nine times lower rates of short-term sickness absence and four times lower rates of long-term sickness absence, while nurses had three times lower rates of short-term sickness absence when compared to other healthcare sector job families [36].

<u>Strengths of the study</u>

The latest Health and Safety Executive (HSE) report lists healthcare staff as one of the categories with the highest sickness absence rates, and mental health and MSK as leading causes [8]. This study is therefore particularly important as we are able to investigate in detail the durations of SA events by specific MSK and MH conditions in this population. The unique and rich EASY database [43] enables analysis of routine data collected in a systemic way across all job categories within the healthcare sector. While several studies have reviewed sickness absence in healthcare settings, these have been limited in several ways. For instance, they examine a narrow range of healthcare workers, mainly doctors and

For peer review only - http://bmjopen.bmj.com/site/about/guidelines.xhtml

nurses; or examine only broad categories of absence [31]. Thus, the size of the EASY database base, giving six full years of sickness absence and return to work data, and the range of variables collected - including demographic, job, start and end dates, self-reported conditions - are considerable strengths.

Study limitations

While the availability of such rich data on routine sickness absence is a major strength of this study, there are also some limitations to the data. This SA management service is only available Monday to Friday and therefore not all absences may have been recorded. To avoid any confounding we removed absences starting on a weekend. The cause of absence is self-reported by the employee when they call the service and not based on a clinical diagnosis, and co-morbidities - which may also impact on absence duration - are not collected [45].

As this health board has a unique SA management service, the results may not be representative of all healthcare workers. However, a national standard requires all health boards in Scotland to work towards a 4% or less sickness absence rate [52]. While NHS Lanarkshire (NHSL) did have a higher SA rate in early 2008, by the end of 2008 the SA rate had fallen to similar levels to the other health boards [12]. The latest data show that the NHSL SA rate of 5.20% is in line with the Scottish NHS average of 5.16% [52], which demonstrates that currently there is not a significant difference in SA rates across Scotland.

Implications for policy and practice

Due to the financial and morale repercussions sickness absence in healthcare employees has not only on

For peer review only - http://bmjopen.bmj.com/site/about/guidelines.xhtml

healthcare staff themselves, but also their patients and employers [23], it is an important focus of attention for healthcare management and public health policies and practice reform. Recent systematic reviews have evaluated the effectiveness of different types of SA interventions and have found that multidisciplinary interventions involving collaboration between employees, health practitioners and employers working to implement tailored modifications for the absentee were consistently more effective than generic non-tailored interventions targeted at all employees [7, 53].

Conclusions

The results of this study further establish the need for occupational health, organizational and management interventions to address recognized individual and workplace stressors that can impact on sickness absence duration. Our results suggest that employees with upper limb disorders and depressive symptoms in particular, require more tailored interventions to assist them in the return to work process following a SA event. A great burden of work loss due to both musculoskeletal and mental health condition was observed for nurses and midwives.

This research is important in terms of improving the health and wellbeing of NHS staff but may also improve the quality of patient care, and subsequently public health. SA has far-ranging economic consequences for both employers and employees, as it simultaneously impacts on NHS resources/service delivery and on people's earnings if the SA is prolonged. These findings can therefore help inform the development of tailored sickness absence interventions for NHS staff.

For peer review only - http://bmjopen.bmj.com/site/about/guidelines.xhtml

Acknowledgements

The authors would like to acknowledge Mark Kennedy, Keith Murray, Moyra Anderson, and Raymond Hamill for their support.

Author Contributions

ED developed the study and was the main author of the manuscript. AB and SS conducted the statistical analysis and contributed to the manuscript writing. JB, KH, SVP and EBM all reviewed and contributed to the manuscript.

Ethical Considerations

NHS Lanarkshire Research and Development (R&D) Management Approval (ref number: L11071).

Funding

This work was supported by the Medical Research Council partnership grant MC/PC/13027 to ED and funding from the UK MRC and Chief Scientist Office (MC_UU_12017/12; SPHSU12) to AB and KH.

Competing Interests

None

For peer review only - http://bmjopen.bmj.com/site/about/guidelines.xhtml

Data Sharing Statement

No additional data available

References

1. Henderson M, Glozier N, Elliott KH. Long term sickness absence - Is caused by common conditions and needs managing. *BMJ* 2005;330(7495):802-3.
2. Ritchie KA, Macdonald EB, Gilmour WH*, et al.* Analysis of sickness absence among employees of four NHS trusts. *Occup Environ Med* 1999;56(10):702-8.
3. Wise J. Audit finds large variations in NHS staff sick days. *BMJ* 2011;342.
4. Vahtera J, Kivimaeki M. Reducing sickness absence in occupational settings. *Occup Environ Med* 2008;65(4):219-20.
5. Fit for Work. http://www.fitforwork.org/. 2015.
6. Health Works. 2009. *A review of the Scottish Government's Healthy Working Lives Strategy,* The Scottish Government, Edinburgh, Available from: http://www.scotland.gov.uk/Publications/2009/12/11095000/0. Edinburgh:
7. van Amelsvoort LG, Jansen NW, Kant I. Addressing long-term sickness absence: moving beyond disease, illness and work-related factors for effective prevention. *Scandinavian journal of work, environment & health* 2017(1):1-4.
8. HSE. 2015. Health and Safety Statistics, Annual report for Great Britain, 2014/2015 (Avaialbale from: http://www.hse.gov.uk/statistics/overall/hssh1415.pdf).
9. Boorman S. NHS Health and Well-being - Final Report. Leeds: Department of Health; 2009. [http://webarchive.nationalarchives.gov.uk/20130107105354/http://www.dh.gov.uk/prod_consum_dh/groups/dh_digitalassets/documents/digitalasset/dh_108907.pdf]. 2009.
10. Suff P. Absence in the NHS. *Occup Health at Work* 2013;10(3):19-23.
11. ONS. Sickness absence in the labour market: february 2014. Available from: http://www.ons.gov.uk/employmentandlabourmarket/peopleinwork/labourproductivity/articles/sicknessabsenceinthelabourmarket/2014-02-25 2014.
12. Brown J, Mackay D, Demou E*, et al.* The EASY (Early Access to Support for You) sickness absence service: a four-year evaluation of the impact on absenteeism. *Scand J Work Environ Health,* 2015;41(2):204-15.
13. Gorman E, Yu S, Alamgir H. When healthcare workers get sick: exploring sickness absenteeism in British Columbia, Canada. *Work (Reading, Mass)* 2010;35(2):117-23.
14. Andersen LL, Clausen T, Burr H*, et al.* Threshold of Musculoskeletal Pain Intensity for Increased Risk of Long-Term Sickness Absence among Female Healthcare Workers in Eldercare. *Plos One* 2012;7(7).
15. Franche RL, Murray EJ, Ostry A*, et al.* Work disability prevention in rural areas: a focus on healthcare workers. *Rural and Remote Health* 2010;10(4).

For peer review only - http://bmjopen.bmj.com/site/about/guidelines.xhtml

16. Murphy IJ. Self-reported and employer-recorded sickness absence in doctors. *Occupational medicine (Oxford, England)* 2014;64(6):417-20.
17. Roelen CAM, Mageroy N, van Rhenen W*, et al.* Low job satisfaction does not identify nurses at risk of future sickness absence: Results from a Norwegian cohort study. *International Journal of Nursing Studies* 2013;50(3):366-73.
18. van der Meer EWC, Boot CRL, van der Gulden JWJ*, et al.* Hand eczema among healthcare professionals in the Netherlands: prevalence, absenteeism, and presenteeism. *Contact Dermatitis* 2013;69(3):164-71.
19. Lalloo D, Demou E, Macdonald EB. Trends in NHS doctor and dentist referrals to occupational health. *Occupational medicine (Oxford, England)* 2016;66(4):316-9.
20. Alexis O. The management of sickness absence in the NHS. *British journal of nursing (Mark Allen Publishing)* 2011;20(22):1437-40, 42.
21. Johnson CJ, Croghan E, Crawford J. The problem and management of sickness absence in the NHS: considerations for nurse managers. *Journal of nursing management* 2003;11(5):336-42.
22. McEwan IM. Absenteeism and sickness Absence. *Postgraduate Medical Journal* 1991;67(794):1067-71.
23. Professor Sir Bruce Keogh KBE. 2013. Section 4.3. Workforce; "Review into the quality of care and treatment provided by 14 hospital trusts in England: overview report".

http://www.nhs.uk/nhsengland/bruce-keogh-review/documents/outcomes/keogh-review-finalreport.Pdf.
24. NICE. Depression: Treatment and Management of Depression in Adults. Clinical Guideline 90. London: Available at www.nice.org.uk. 2009.
25. NICE. Anxiety: Management of Anxiety (Panic Disorder, With and Without Agoraphobia, and Generalised Anxiety Disorder) in Adults in Primary, Secondary and Community Care. Clinical Guideline 113. London: . Available at www.nice.org.uk. 2011.
26. Wiberg M, Friberg E, Palmer E*, et al.* Sickness absence and subsequent disposable income: A population-based cohort study. *Scand J Public Health* 2015;43(4):432-40.
27. Hoy D, March L, Brooks P*, et al.* The global burden of low back pain: estimates from the Global Burden of Disease 2010 study. *Annals of the rheumatic diseases* 2014;73(6):968-74.
28. Wynne-Jones G, Cowen J, Jordan JL*, et al.* Absence from work and return to work in people with back pain: a systematic review and meta-analysis. *Occupational and Environmental Medicine* 2013.
29. Ariens GA, Bongers PM, Hoogendoorn WE*, et al.* High physical and psychosocial load at work and sickness absence due to neck pain. *Scandinavian journal of work, environment & health* 2002;28(4):222-31.
30. Dewa CS, Loong D, Bonato S. Work outcomes of sickness absence related to mental disorders: a systematic literature review. *BMJ open* 2014;4(7).
31. Michie S, Williams S. Reducing work related psychological ill health and sickness absence: a systematic literature review. *Occup Environ Med* 2003;60(1):3-9.
32. Judd LL, Schettler PJ, Solomon DA*, et al.* Psychosocial disability and work role function compared across the long-term course of bipolar I, bipolar II and unipolar major depressive disorders. *Journal of Affective Disorders* 2008;108(1-2):49-58.

For peer review only

For peer review only - http://bmjopen.bmj.com/site/about/guidelines.xhtml

33. Soderman E, Lisspers J, Sundin O. Depression as a predictor of return to work in patients with coronary artery disease. *Social science & medicine (1982)* 2003;56(1):193-202.
34. van den Brink RHS, Ormel J, Tiemens BG*, et al.* Predictability of the one-year course of depression and generalized anxiety in primary care. *General Hospital Psychiatry* 2002;24(3):156-63.
35. Munir F, Yarker J, Haslam C*, et al.* 2009. Returning to Work - The Role of Depression. Available from: http://www.mentalhealth.org.uk/publication-download/returning-work-role-depression. Loughborough,:
36. Lim A, Chongsuvivatwong V, Geater A*, et al.* Influence of work type on sickness absence among personnel in a teaching hospital. *Journal of Occupational Health* 2002;44(4):254-63.
37. Sandanger I, Nygard JF, Brage S*, et al.* Relation between health problems and sickness absence: gender and age differences--a comparison of low-back pain, psychiatric disorders, and injuries. *Scand J Public Health* 2000;28(4):244-52.
38. Krane L, Johnsen R, Fleten N*, et al.* Sickness absence patterns and trends in the healthcare sector: 5-year monitoring of female municipal employees in the health and care sectors in Norway and Denmark. *Human Resources for Health* 2014;12.
39. Kristensen TR, Jensen SM, Kreiner S*, et al.* Socioeconomic status and duration and pattern of sickness absence. A 1-year follow-up study of 2331 hospital employees. *BMC public health* 2010;10.
40. d'Errico A, Costa G. Socio-demographic and work-related risk factors for medium- and long-term sickness absence among Italian workers. *European journal of public health* 2012;22(5):683-8.
41. MacEachen E, Clarke J, Franche R-L*, et al.* Systematic review of the qualitative literature on return to work after injury. *Scandinavian journal of work, environment & health* 2006;32(4):257-69.
42. Macdonald EB, Asanati K. Absence from work. *Contemporary Social Science* 2016;11(1):30-9.
43. Demou E, Brown J, Sanati K*, et al.* A novel approach to early sickness absence management: The EASY (Early Access to Support for You) way. *Work (Reading, Mass)* 2015;53(3):597-608.
44. Engel GL. Need for a new medical model - Challenge for biomedicine. *Science* 1977;196(4286):129-36.
45. Ervasti J, Mattila-Holappa P, Joensuu M*, et al.* Predictors of Depression and Musculoskeletal Disorder Related Work Disability Among Young, Middle-Aged, and Aging Employees. *Journal of Occupational and Environmental Medicine* 2017;59(1):114-9.
46. Lotters F, Burdorf A. Prognostic factors for duration of sickness absence due to musculoskeletal disorders. *The Clinical journal of pain* 2006;22(2):212-21.
47. Nieuwenhuijsen K, Verbeek JH, de Boer A*, et al.* Predicting the duration of sickness absence for patients with common mental disorders in occupational healthcare. *Scandinavian Journal of Work Environment & Health* 2006;32(1):67-74.
48. Holtermann A, Hansen JV, Burr H*, et al.* Prognostic factors for long-term sickness absence among employees with neck-shoulder and low-back pain. *Scandinavian journal of work, environment & health* 2010;36(1):34-41.
49. Armijo-Olivo S, Woodhouse LJ, Steenstra IA*, et al.* Predictive value of the DASH tool for predicting return to work of injured workers with musculoskeletal disorders of the upper extremity. *Occupational and Environmental Medicine* 2016.
50. Williams S, Michie S, Pattani S. Improving the health of the NHS workforce. London: Nuffield Trust; http://discovery.ucl.ac.uk/16094/. 1998.
51. Cox T. The nature and measurement of stress. *Ergonomics* 1985;28(8):1155-63.

For peer review only - http://bmjopen.bmj.com/site/about/guidelines.xhtml

52. ISD NHS. 2016. NHSScotland Workforce Information. Quarterly update of Staff in Post, Vacancies and Turnover at 31 March 2016. Available from: https://www.isdscotland.org/Health-Topics/Workforce/Publications/2016-06-07/2016-06-07-Workforce-Report.pdf.

53. Durand MJ, Corbiere M, Coutu MF*, et al.* A review of best work-absence management and return-to-work practices for workers with musculoskeletal or common mental disorders. *Work (Reading, Mass)* 2014;48(4):579-89.

For peer review only

For peer review only - http://bmjopen.bmj.com/site/about/guidelines.xhtml

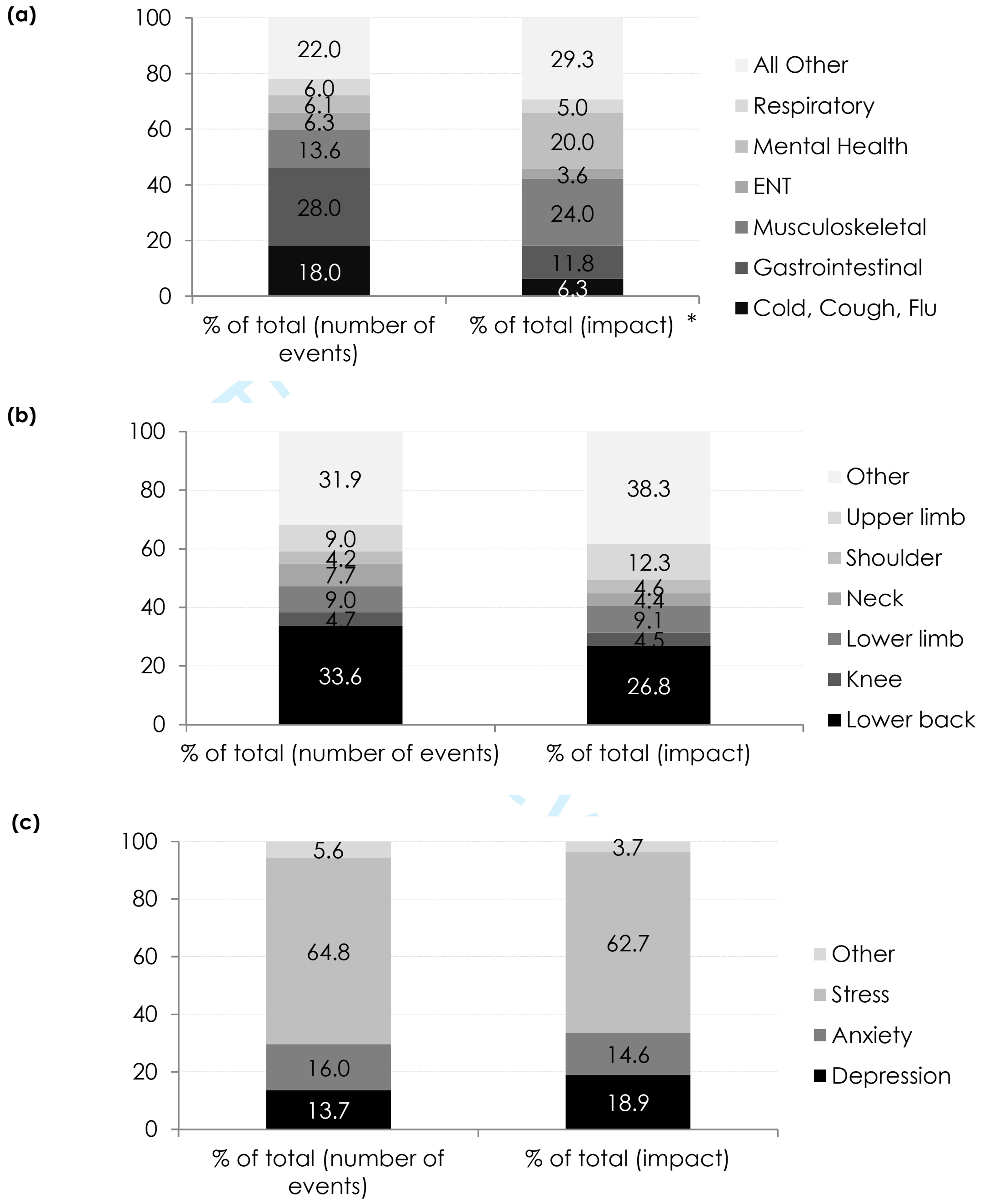

Figure 1. Cause of sickness absence (% of total number of events & % Impact) for (a) all conditions, (b) MSK conditions**, and (c) mental health conditions***

*Impact is estimated as percent impact and calculated by number of events times the average condition-specific absence duration divided by the sum of impact for all causes times 100
**Number of absences due to 'Hip' were to small and grouped into 'Other' category
*** Number of absences due to 'Bipolar Disorder', 'Schizophrenia', 'Panic Attacks' and 'Self harm' were to small and grouped into 'Other' category

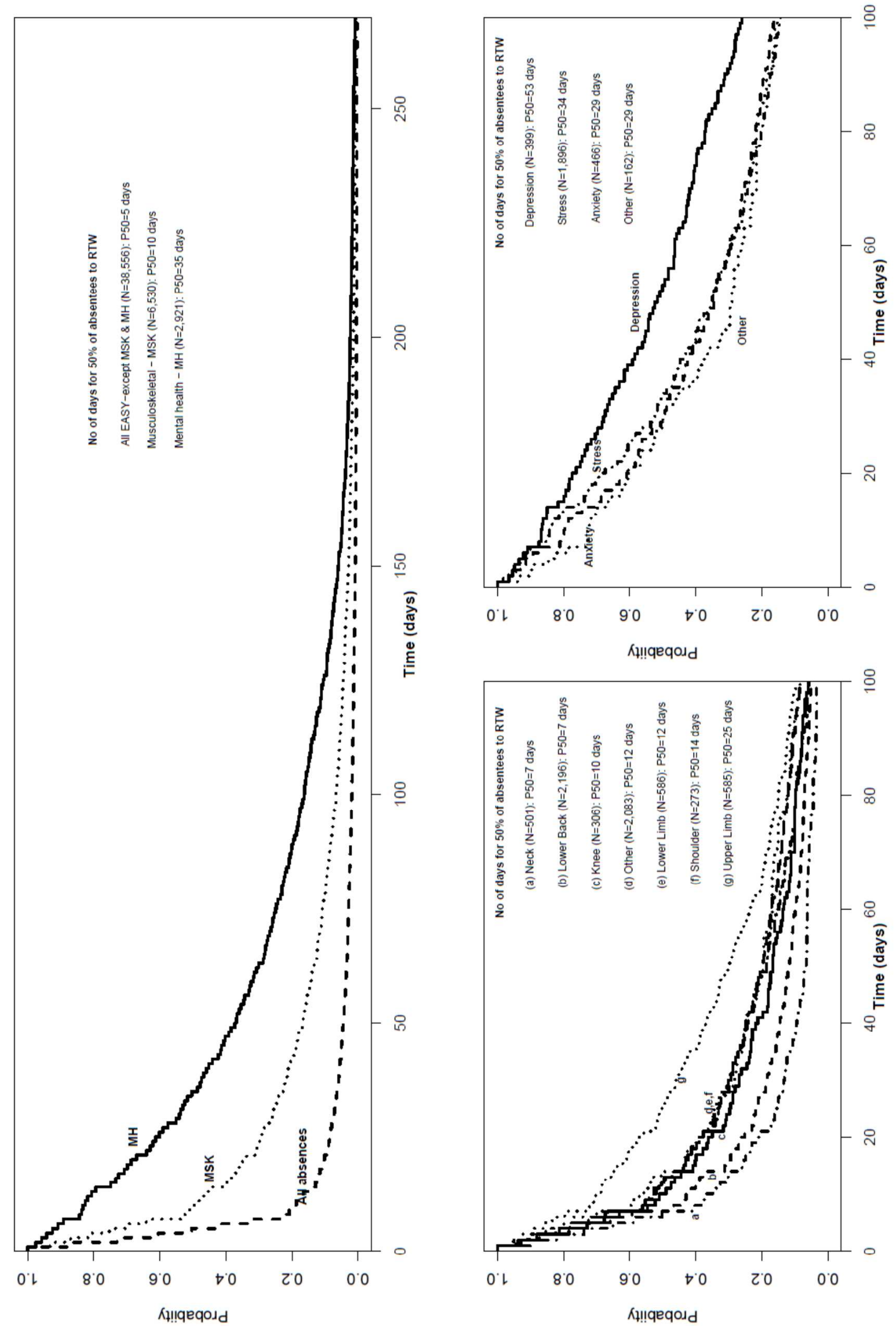

Figure 2. a. Return to Work curves for (top) all absences; (bottom-left) b. MSK-related absences by MSK condition; and (bottom-right) c. MH-related absences by MH condition

Sickness Absence Duration in Healthcare Workers: The Impact of Musculoskeletal and Mental Health Problems on Duration of Absence

[Supplementary material]

Evangelia Demou PhD[1], Shanley Smith[2], Abita Bhaskar[1], Judith Brown PhD[2], Kate Hunt[1], Sergio Vargas-Prada MD[3,4], Ewan B Macdonald MD[2]

1. MRC/CSO Social and Public Health Sciences Unit, University of Glasgow, Glasgow, G2 3QB, United Kingdom

2. Healthy Working Lives Group, University of Glasgow, Glasgow, G12 8RZ, United Kingdom

3. Center for Research in Occupational Health (CiSAL), Universitat Pompeu Fabra, Barcelona, Spain

4. Unidad Central de Contingencias Comunes (U3C), ASEPEYO, Barcelona, Spain.

Running title Sickness Absence Duration in Healthcare Workers

Corresponding Author

Dr Evangelia Demou

Research Fellow

MRC/CSO Social and Public Health Sciences Unit

University of Glasgow

200 Renfield Street, Glasgow, G2 3QB, UK

Tel: 0141 353 7580

For peer review only

For peer review only - http://bmjopen.bmj.com/site/about/guidelines.xhtml

Figure S1a. Mean duration (in days) of absence by cause of sickness

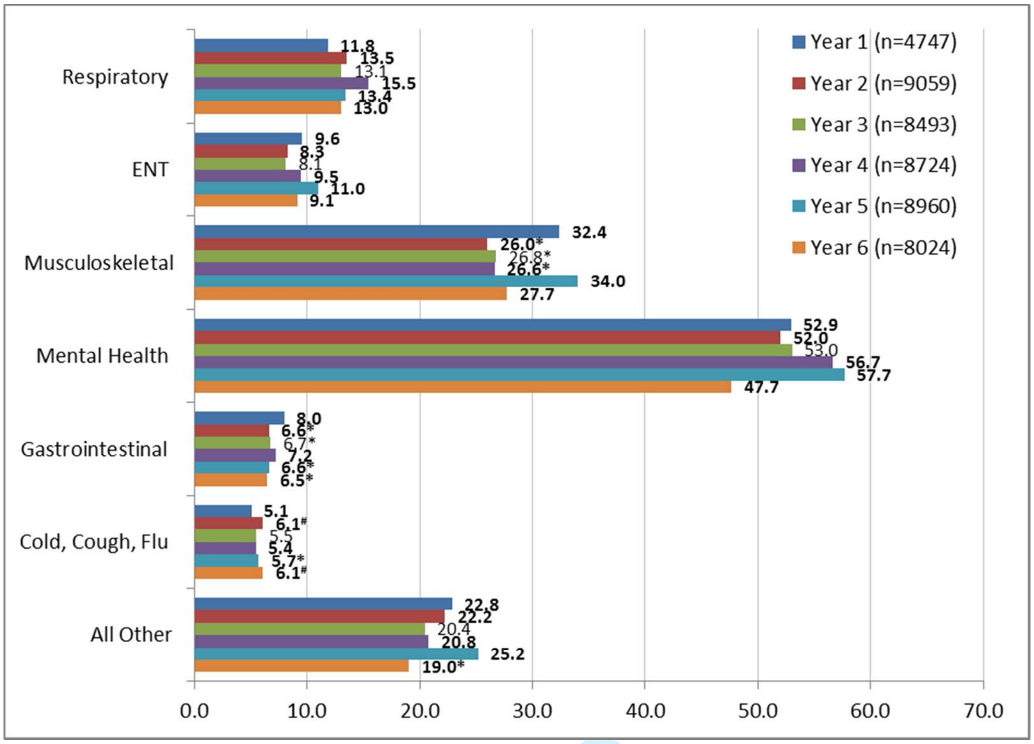

Figure S1b. Mean duration (in days) of absence by MSK cause of sickness

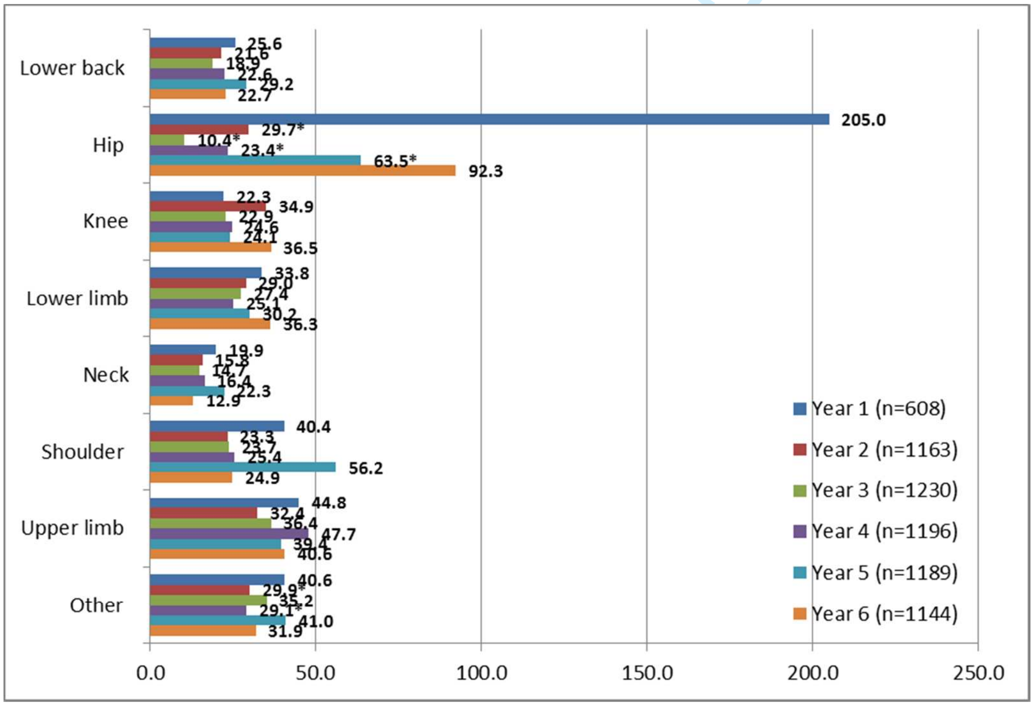

For peer review only - http://bmjopen.bmj.com/site/about/guidelines.xhtml

Figure S1c. Mean duration (in days) of absence by MH cause of sickness

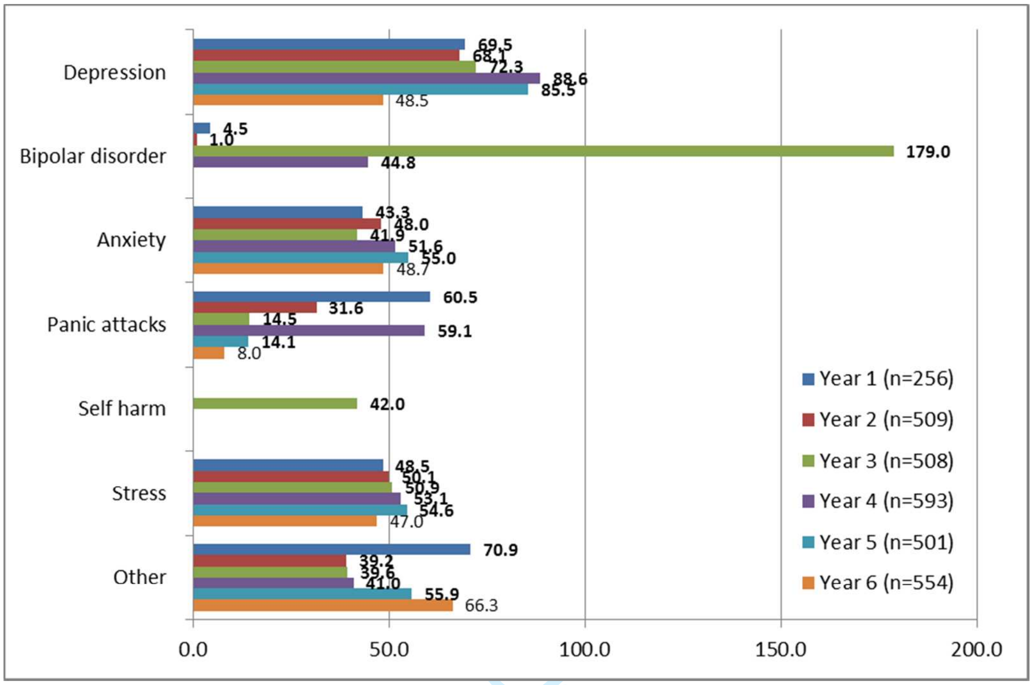

Table S1a. Multivariate cox regression hazard ratios for full population, adjusted for sex, age, job category, job type, day of first absence, season of absence and year of absence.

All	HR	95% CI	P
Day of absence			
Mon	1		
Tues	1.07	(1.04, 1.10)	0.000
Wed	1.02	(0.99, 1.05)	0.155
Thurs	1.02	(0.99, 1.05)	0.263
Fri	0.96	(0.93, 1.00)	0.026
Season			
Spring	1		
Summer	1.00	(0.97, 1.03)	0.960
Autumn	1.01	(0.98, 1.03)	0.675
Winter	1.01	(0.99, 1.04)	0.238

For peer review only - http://bmjopen.bmj.com/site/about/guidelines.xhtml

Table S1b. Multivariate cox regression hazard ratios for staff with musculoskeletal conditions, adjusted for sex, age, job category, job type, day of first absence, season of absence and year of absence.

Musculoskeletal	HR	95% CI	P
Day of absence			
Mon	1		
Tues	1.06	(0.99, 1.14)	0.088
Wed	1.07	(1.00, 1.15)	0.063
Thurs	1.08	(1.01, 1.17)	0.031
Fri	1.01	(0.93, 1.10)	0.753
Season			
Spring	1		
Summer	1.01	(0.94, 1.08)	0.793
Autumn	1.01	(0.94, 1.08)	0.778
Winter	1.01	(0.94, 1.08)	0.772

Table S1c. Multivariate cox regression hazard ratios for staff with mental health conditions, adjusted for sex, age, job category, job type, day of first absence, season of absence and year of absence.

Mental health	HR	95% CI	P
Job type			
Part time	1		
Full time	1.08	(1.00, 1.17)	0.048
Day of absence			
Mon	1		
Tues	1.03	(0.93, 1.15)	0.540
Wed	1.00	(0.90, 1.11)	0.993
Thurs	0.99	(0.89, 1.11)	0.917
Fri	0.90	(0.80, 1.02)	0.089
Season			
Spring	1		
Summer	1.03	(0.92, 1.14)	0.649
Autumn	1.06	(0.96, 1.18)	0.235
Winter	1.02	(0.92, 1.13)	0.689

For peer review only

STROBE Statement—checklist of items that should be included in reports of observational studies

	Item No.	Recommendation	Page No.	Relevant text from manuscript
**Title and abstract**	1	(*a*) Indicate the study's design with a commonly used term in the title or the abstract	2	This cross-sectional study…..
		(*b*) Provide in the abstract an informative and balanced summary of what was done and what was found	2	Survival analyses and Cox's proportional hazards models were used to estimate SA duration……
**Introduction**				
Background/rationale	2	Explain the scientific background and rationale for the investigation being reported	4-7	
Objectives	3	State specific objectives, including any prespecified hypotheses	7	Study aim
**Methods**				
Study design	4	Present key elements of study design early in the paper	8	
Setting	5	Describe the setting, locations, and relevant dates, including periods of recruitment, exposure, follow-up, and data collection	8-9	
Participants	6	(*a*) *Cohort study*—Give the eligibility criteria, and the sources and methods of selection of participants. Describe methods of follow-up<br>*Case-control study*—Give the eligibility criteria, and the sources and methods of case ascertainment and control selection. Give the rationale for the choice of cases and controls<br>*Cross-sectional study*—Give the eligibility criteria, and the sources and methods of selection of participants	8-10	
		(*b*) *Cohort study*—For matched studies, give matching criteria and number of exposed and unexposed<br>*Case-control study*—For matched studies, give matching criteria and the number of controls per case		
Variables	7	Clearly define all outcomes, exposures, predictors, potential confounders, and effect modifiers. Give diagnostic criteria, if applicable	9-10	
Data sources/ measurement	8*	For each variable of interest, give sources of data and details of methods of assessment (measurement). Describe comparability of assessment methods if there is more than one group	*9-10*	
Bias	9	Describe any efforts to address potential sources of bias	9-10	multivariate model controlled for several occupational and individual
Study size	10	Explain how the study size was arrived at	8-9	

Continued on next page

For peer review only - http://bmjopen.bmj.com/site/about/guidelines.xhtml

Quantitative variables	11	Explain how quantitative variables were handled in the analyses. If applicable, describe which groupings were chosen and why	9-10	
Statistical methods	12	(*a*) Describe all statistical methods, including those used to control for confounding	9-10	
		(*b*) Describe any methods used to examine subgroups and interactions	8-10	
		(*c*) Explain how missing data were addressed	8-9	
		(*d*) *Cohort study*—If applicable, explain how loss to follow-up was addressed<br>*Case-control study*—If applicable, explain how matching of cases and controls was addressed<br>*Cross-sectional study*—If applicable, describe analytical methods taking account of sampling strategy	9	
		(*e*) Describe any sensitivity analyses		
**Results**				
Participants	13*	(a) Report numbers of individuals at each stage of study—eg numbers potentially eligible, examined for eligibility, confirmed eligible, included in the study, completing follow-up, and analysed	10-11	
		(b) Give reasons for non-participation at each stage	na	
		(c) Consider use of a flow diagram	na	
Descriptive data	14*	(a) Give characteristics of study participants (eg demographic, clinical, social) and information on exposures and potential confounders	11	Table 1
		(b) Indicate number of participants with missing data for each variable of interest	na	
		(c) *Cohort study*—Summarise follow-up time (eg, average and total amount)		
Outcome data	15*	*Cohort study*—Report numbers of outcome events or summary measures over time		
		Case-control study—Report numbers in each exposure category, or summary measures of exposure		
		Cross-sectional study—Report numbers of outcome events or summary measures	*11& 14*	*Tables 1 &2*
Main results	16	(*a*) Give unadjusted estimates and, if applicable, confounder-adjusted estimates and their precision (eg, 95% confidence interval). Make clear which confounders were adjusted for and why they were included	14 & 16	
		(*b*) Report category boundaries when continuous variables were categorized		
		(*c*) If relevant, consider translating estimates of relative risk into absolute risk for a meaningful time period		

Continued on next page

For peer review only - http://bmjopen.bmj.com/site/about/guidelines.xhtml

Other analyses	17	Report other analyses done—eg analyses of subgroups and interactions, and sensitivity analyses	17	The analysis by year of absence…
**Discussion**				
Key results	18	Summarise key results with reference to study objectives	17-18	Summary of findings section
Limitations	19	Discuss limitations of the study, taking into account sources of potential bias or imprecision. Discuss both direction and magnitude of any potential bias	20	Study limitations section
Interpretation	20	Give a cautious overall interpretation of results considering objectives, limitations, multiplicity of analyses, results from similar studies, and other relevant evidence	17-20	
Generalisability	21	Discuss the generalisability (external validity) of the study results	20	The latest data show that the NHSL SA rate is in line with the Scottish NHS average
**Other information**				
Funding	22	Give the source of funding and the role of the funders for the present study and, if applicable, for the original study on which the present article is based	22	Funding section

*Give information separately for cases and controls in case-control studies and, if applicable, for exposed and unexposed groups in cohort and cross-sectional studies.

Note: An Explanation and Elaboration article discusses each checklist item and gives methodological background and published examples of transparent reporting. The STROBE checklist is best used in conjunction with this article (freely available on the Web sites of PLoS Medicine at http://www.plosmedicine.org/, Annals of Internal Medicine at http://www.annals.org/, and Epidemiology at http://www.epidem.com/). Information on the STROBE Initiative is available at www.strobe-statement.org.

For peer review only

For peer review only - http://bmjopen.bmj.com/site/about/guidelines.xhtml

Evaluating Sickness Absence Duration by Musculoskeletal and Mental Health Issues. A retrospective study of Scottish Healthcare Workers.

Journal:	*BMJ Open*
Manuscript ID	bmjopen-2017-018085.R1
Article Type:	Research
Date Submitted by the Author:	03-Oct-2017

Complete List of Authors:	Demou, Evangelia; University of Glasgow, MRC/CSO Social and Public Health Sciences Unit; University of Glasgow, Public Health, Institute of Health and Wellbeing<br>Smith, Shanley; University of Glasgow, Public Health, Institute of Health and Wellbeing<br>Bhaskar, Abita; University of Glasgow, MRC/CSO Social and Public Health Sciences Unit<br>Brown, Judith; University of Glasgow, Public Health, Institute of health and Wellbeing<br>Hunt, Kate; University of Glasgow, MRC/CSO Social and Public Health Sciences Unit<br>Vargas, Sergio; Pompeu Fabra University, Center for Research in Occupational Health (CiSAL)<br>Macdonald, Ewan; The University of Glasgow, Healthy working lives group

**Primary Subject Heading**:	Occupational and environmental medicine
Secondary Subject Heading:	Epidemiology, Health policy, Mental health, Occupational and environmental medicine, Public health
Keywords:	MENTAL HEALTH, healthcare worker, anxiety, depression, Musculoskeletal disorders < ORTHOPAEDIC & TRAUMA SURGERY, return to work

SCHOLARONE™
Manuscripts

Evaluating Sickness Absence Duration by Musculoskeletal and Mental Health Issues. A retrospective study of Scottish Healthcare Workers.

Evangelia Demou PhD[1], Shanley Smith[2], Abita Bhaskar[1], Judith Brown PhD[2], Kate Hunt PhD[1], Sergio Vargas-Prada MD PhD[3,4,5,6], Ewan B Macdonald MD[2]

1. MRC/CSO Social and Public Health Sciences Unit, University of Glasgow, Glasgow, G2 3QB, United Kingdom

2. Public Health, Institute of Health and Wellbeing, University of Glasgow, Glasgow, G12 8RZ, United Kingdom

3. Center for Research in Occupational Health (CiSAL), Universitat Pompeu Fabra, Barcelona, Spain

4. Unidad Central de Contingencias Comunes (U3C), ASEPEYO, Barcelona, Spain.

5. CIBER Epidemiología y Salud Pública (CIBERESP), Spain.

6. IMIM (Hospital del Mar Research Institute), Barcelona, Spain

Running title Sickness Absence Duration in Healthcare Workers

Word count: 4,298

Number of figures: 2

Number of tables: 2

Key terms work; return to work; nurses; doctors, back pain, depression, anxiety, healthcare worker

Corresponding Author
Dr Evangelia Demou
Research Fellow
MRC/CSO Social and Public Health Sciences Unit
University of Glasgow
200 Renfield Street, Glasgow, G2 3QB, UK
Email: evangelia.demou@glasgow.ac.uk
Tel: 0141 353 7580

For peer review only

For peer review only - http://bmjopen.bmj.com/site/about/guidelines.xhtml

Abstract

Objectives: Sickness absence (SA) among healthcare workers is associated with occupational and non-occupational risk factors, and impacts employee health, healthcare delivery and patient health. At the same time, it is one of the industries with the highest rates of work-related ill-health in the UK. Musculoskeletal (MSK) and mental health (MH) issues are leading causes of SA, but there is a lack of research on how certain MSK/MH conditions impact on SA duration. The study aim is to determine differences in SA duration by MH and MSK disorders in healthcare employees.

Methods: Survival analyses were used to estimate SA duration due to MSK and MH problems over six years and Cox's proportional hazards models to determine the hazard ratios of the absentees returning to work, using a Scottish health board bespoke database with over 53 thousand SA events. SA duration and time to return-to-work were estimated for employees by age, gender, job and health conditions.

Results: MSK and MH conditions accounted for 27% and 6%, respectively, of all SA events and 23.7% and 19.5% of all days lost. Average SA duration over the study period was 43.5 days for MSK and 53.9 days for MH conditions. For MSK conditions, employees with low back pain had the fastest return-to-work (P_{50}: 7 days), while employees absent due to depression took the longest (P_{50}: 54 days). The most influential socio-demographic variables affecting return-to-work were age, gender, and job category.

Conclusions: This longitudinal study used a unique and rich database to quantify SA duration by cause for certain MSK and MH-related SA events. Our findings can be used by public health practitioners and healthcare managers to develop and implement tailored and targeted workplace interventions for employees with MSK and MH problems.

For peer review only - http://bmjopen.bmj.com/site/about/guidelines.xhtml

Article summary - Strengths and limitations of this study

- Healthcare is one of the sectors with significantly higher rates of sickness absence (SA), impacting on employee health, healthcare delivery and patient health. This study uses a unique, data-rich and bespoke database that allows for detailed assessment of the impact of MSK and MH conditions on absence duration and return to work for healthcare employees of a Scottish health board.

- Survival analyses and Cox's proportional hazards models applying a time varying coefficient were used to estimate SA duration due to musculoskeletal (MSK) and mental health (MH) problems over six years (N=48,007 unique SA events).

- The size and granularity of the database, giving six full years of sickness absence and return to work data, and the range of variables collected - including demographic, job, start and end dates, self-reported conditions, allowed for allowed for the investigation of SA duration and time to return-to-work for healthcare employees by age, gender, job and health conditions.

- SA data are from a single large health board (c.12,000 employees) which limits the degree of generalizability. Also as SA absence is multi-causal and it is necessary to consider an individual's work and workplace environment, there are a number of variables that are not collected as part of the service (e.g. operational and organizational variables) and could potentially be important risk factors for SA and RTW in this population.

For peer review only

For peer review only - http://bmjopen.bmj.com/site/about/guidelines.xhtml

Introduction

Sickness absence (SA) is a significant public health burden on government, employees, employers, and public resources [1-5]. This is due to loss of productivity, increased workload on other staff, as well as resources spent to cover incapacity to work and restore health [2 6]. SA data is increasingly being used as a measure of ill-health [7]. The public health burden of sickness absence is recognized and governments and employers are developing policies toward providing support to employers and employees in improving health, reducing sickness absence rates, and improving return to work (RTW) times [8-12].

Multiple factors need to be considered to effectively manage SA through policy and practice [13]. Sickness absence rates vary by sector and employer, with the healthcare sector exhibiting one of the highest rates[14]. Additionally, within the health service, variations in SA rates depend on region, job category and salary grade, among other factors [3 15 16]. Cause of SA can have a major impact on duration and overall costs. Long term sickness absence (LTSA), although only responsible for a small percentage of SA *events*, makes up approximately 75% of absence *costs*, with musculoskeletal (MSK) and mental health (MH) disorders being two of the leading causes of LTSA [2 3 14 17 18].

Healthcare Employees and Sickness Absence

The healthcare sector is a diverse entity, which presents a unique set of challenges in relation to SA duration [19]. Studies show that healthcare workers experience high exposures to both occupational and non-occupational risk factors, negatively impacting on SA events [19-24]. Healthcare employee absence leads to increases in the risks to quality of patient care, workload stress of colleagues, and employers'

For peer review only - http://bmjopen.bmj.com/site/about/guidelines.xhtml

staffing costs [19 25-28]. The most recent 2016 figures from the Health and safety Executive (HSE) report the healthcare industry of ones of the industries with the highest rates of work-related illness [29]. Ill health in the health and social care sector leads to around 4.8 million working days lost with the majority due to mental health disorders, followed by work-related musculoskeletal disorders [30]. A study examining quality of care and treatment in 14 English hospital trusts found a positive association between the in-patient to staff ratio and high hospital standardized mortality ratio (HSMR) scores [31] and also identified insufficient nursing establishments and poor staffing levels on night shifts and weekends, partially due to high SA rates [31].

Impact of Sickness Absence Cause and Duration

Certain health conditions result in longer periods of SA [14]. These thus have large potential interest as the focus of workplace interventions, due to the greater impact longer SA spells have on individuals and employers [32 33]. There are potential long term effects on individuals who experience particular types of SA events; one Swedish cohort study, for example, found an association between long term absence and lower disposable income in the 2-6 years following the absence event [34]. A previous study investigating the effectiveness of an early SA management service in a Scottish Health Board, found that although the leading causes of SA were gastrointestinal problems, followed by cold/cough/flu, the greatest impact on total number of days lost were due to musculoskeletal (MSK) and mental health (MH) problems [18].

MSK related absence

Health Response UK reports musculoskeletal injury as the leading cause of absenteeism and related cost

For peer review only

to UK Industry, with back pain as one of the most common causes of MSK-related sickness absence [17]. Back pain is often recurrent, and the majority of people who have activity-limiting back pain go on to have recurrent SA episodes [35]. A systematic review examining SA and return-to-work (RTW), found that approximately 20% of employees with back pain have some sickness absence episodes in the six to 12 month period following the back pain episode. RTW in this review was estimated at up to one month for 68% of individuals, 85% of individuals were back to work 1–6 months and 93% were back to work at 6 months post the SA event [36]. Although RTW rates for back pain tend to be high, the recurrent nature of back pain increases the risk for substantial overall impact on work days lost through repeated periods of SA.

Neck problems are also a common MSK condition resulting in lost working days [17]. One cohort study investigating the relationship between physical and psychosocial features of the workplace and SA, found that work activities involving neck flexion and neck rotation, high job demands, low skill discretion, and low job security were significantly associated with SA due to neck pain [37].

Mental Health related absence

A number of systematic reviews highlight the importance of recognizing mental health issues in the workplace to assist in the reduction of associated SA [38 39]. However, there is limited evidence on how certain mental health conditions affect RTW times.

Depression is a leading cause of MH-related absence [14]. The extent and severity of depressive symptoms, comorbidity of anxiety, social and emotional support, education, and long symptom periods prior to diagnosis can affect the course of depression and RTW times [40-42].

For those experiencing anxiety that results in SA, previous anxiety episodes, older age, education, and

For peer review only - http://bmjopen.bmj.com/site/about/guidelines.xhtml

long durations of untreated and undiagnosed symptoms, contribute to longer absences [42].

Detailed return-to-work figures following LTSA are scarce. Estimates suggest that as few as 13% of people who have experienced depression and anxiety are in employment, compared to 33% of people experiencing other chronic illnesses [43].

Socio-demographic and Occupational Factors and Sickness Absence

Several studies have investigated the associations between sickness absence and an employee's socio-demographic and socio-economic characteristics [19 44-48]. Evidence shows that sickness absence is multi-causal, and that in addition to providing support for an individual's specific characteristics and incapacitating condition, it is necessary to also consider an individual's work and workplace environment in order to effectively manage and improve return to work times [49 50].

Aim

Despite a great deal of research recognizing the prevalence of MSK and MH issues and their impact on SA rates [3 19-24]; there remains a lack of information on how certain types of musculoskeletal and mental health conditions may affect SA duration, which is vital to inform and improve current and new SA interventions, as well as support workplace modifications for SA prevention. This study aims to examine the impact of certain musculoskeletal and mental health conditions on sickness absence duration in healthcare workers, using a bespoke database [18 51] over a six year period, and to assess how health, socio-demographic factors, work characteristics and occupational factors affect SA duration.

For peer review only - http://bmjopen.bmj.com/site/about/guidelines.xhtml

Methods

Study population

The study population includes all participants in the EASY (Early Access to Support for You) SA management service of a Scottish health board [18 51]. EASY is a telephone-based service that provides early intervention (from Day 1 of absence) based on the biopsychosocial model [51 52] . Data on all staff who voluntarily engage with EASY (compliance rate was c.80%) are routinely entered into a bespoke database, including age, gender, job family, cause of absence, date of first day of absence, and return to work date [18 51]. Detailed descriptions of the EASY service can be found elsewhere [18 51]. We examined data on 66 490 unique absence events recorded in the EASY database between May 2008 and December 2014.

Defining and Recoding Variables

Sickness absence duration was calculated from the difference between the first date of absence and the RTW date. Cause of absence was grouped into eight categories: Respiratory, Musculoskeletal, Mental Health, Gastrointestinal, ENT, Cough/Cold/Flu, and All Other. Conditions assigned to the musculoskeletal category were grouped into nine subgroups according to anatomical site: Hip, Knee, Low Back, Lower Limb, Neck, Shoulder, Upper Limb, and All Other. Mental health conditions were allocated to eight subgroups according to diagnostic group: Bipolar Disorder, Anxiety, Depression, Schizophrenia, Panic attacks, Self-Harm, Stress, and All Other Psychiatric Disorders.

After missing data were eliminated (<0.05%), four main exclusion criteria were applied: (a) if there was no first day of absence (FDA) (N=196); (b) if the FDA was a Saturday or Sunday (N=4881), as there was no SA service on those days and absences would not be recorded on FDA; (c) if the 'date opened' (i.e. the

For peer review only - http://bmjopen.bmj.com/site/about/guidelines.xhtml

date the EASY service contacted the absentee) was before the first day of absence (N=828); (d) if the 'date opened' was equal to or after the RTW date (N=3 465). Due to overlaps among the missing data and exclusion criteria, a total of 13 286 absences were excluded, giving a total of 53 193 unique absence events; hence analysis was based on 53 193 unique absence events.

Analytic Strategy

Descriptive Statistics

Descriptive statistics and crosstabs were produced for the entire EASY population, which included all causes of sickness absence (N=53 193). Descriptive statistics and crosstabs were then produced for two specific causes, MH (N=3 093) and MSK (N=6 969) conditions.

Mean absence duration was calculated for the entire EASY population in the study, the entire EASY population without MSK and MH (N=43 131), MSK only, MH only, MSK sub categories, and MH sub categories for each year from May 2008 to December 2015. The data were divided into seven years as follows: May 2008–April 2009 (Year 1); May 2009–April 2010 (Year 2); May 2010–April 2011 (Year 3); May 2011–April 2012 (Year 4); May 2012-April 2013 (Year 5); May 2013-April 2014 (Year 6); May 2014-December 2015 (Year 7). Absences commencing in Year 7 were not included as the year was incomplete and in numerous cases absence could still be ongoing; hence further analyses presented were based on 48 007 unique absence events.

Kaplan Meier Survival Analysis & Cox Proportional Hazards Model

For peer review only

Absence duration was analyzed using Kaplan Meier survival analyses and Cox's proportional hazards models to determine the hazard (risk) of absentees returning to work. We used a time-varying coefficient in our analysis to correct for non-compliance to the Cox assumption. The model takes into account each sickness absence event as well as individuals with multiple absence events by calculating cluster robust standard errors and the multivariate model controlled for several occupational and individual variables including: gender, age, job family, job type, cause of absence, day of absence, season of absence, and year of absence (Table S1).

All statistical analyses were conducted using STATA version 12.0 and R version 3.1.1.

Results

Our sample contains 11,694 individual people and 48,007 absences (May 08 – April 14). Descriptive statistics for our sample (May08-Dec14; N=48,007) are reported in **Table 1**. Almost 9 out of 10 (87.9%) sample's absences were from females (N=42,187). Over the six year period investigated, the three most common causes of SA events are gastrointestinal (N=13,459), cold, cough, and flu (N=8,657), and musculoskeletal (N=6,530) problems. Almost half the absences (45.3%) were from the nursing/midwifery job category, and a further 20% in administrative services. Numbers in part-time (43.2%) and full-time employment (56.8%) were relatively similar.

Fewer of those with MSK-related absences (N=6,530) were in the two youngest age groups, than in the total population, and the proportion of those with MSK absences who were in the Nursing/midwifery (N=3,428) job category (52.5%) was even higher than seen in the total population (45.3%). In the MH

For peer review only - http://bmjopen.bmj.com/site/about/guidelines.xhtml

population, 91.8% are female, compared to 87.9% of the total population (Table 1).

Table 1. Descriptive statistics for total EASY population, and MSK and MH subgroups

		Total Sample (N=48,007)		Total MSK Absences (N=6,530)		Total MH Absences (N=2,921)	
		N	%	N	%	N	%
Age Group	16-29	6,885	14.34	588	9.00	253	8.66
	30-39	10,772	22.44	1,124	17.21	639	21.88
	40-49	15,257	31.78	2,291	35.08	1,102	37.73
	50-59	13,003	27.09	2,181	33.40	813	27.83
	60+	2,090	4.35	346	5.30	114	3.90
Gender	Male	5,820	12.12	963	14.75	241	8.25
	Female	42,187	87.88	5,567	85.25	2,680	91.75
Job Family	Administrative Services	9,597	19.99	1,003	15.36	550	18.83
	Allied Health Profession	4,914	10.24	502	7.69	208	7.12
	Healthcare Sciences	2,130	4.44	284	4.35	105	3.59
	Manager	150	0.31	13	0.20	5	0.17
	Medical & Dental	1,301	2.71	125	1.91	33	1.13
	Medical & Dental Support	876	1.82	83	1.27	35	1.20
	Nursing/Midwifery	21,734	45.27	3,428	52.50	1,614	55.26
	Other Therapeutic	2,100	4.37	193	2.96	74	2.53
	Personal and Social Care	443	0.92	56	0.86	18	0.62
	Support Services	4,762	9.92	843	12.91	279	9.55
Job Type	Part time	20,758	43.24	2,871	43.97	1,431	48.99
	Full time	27,249	56.76	3,659	56.03	1,490	51.01

For peer review only

Mean duration (in days) of absence by cause of sickness

Gastrointestinal (GI) and cold, cough, and flu (CCF) problems account for the largest number of sickness absence events, 28% and 18%, respectively. However, Figure 1a shows that the impact, in number of days absent, these causes have on total number of working days lost is much less (11.8% for GI and 6.3% for CCF). The health conditions with the highest impact on total number of working days lost are musculoskeletal (24%) and mental health (20%) conditions. Mean absence duration ranged from 5.6 days for CCF to 53.3 days for MH-related absences. MSK absences had an overall mean duration of 28.9 days (Figures S1a,b,c in the Supplementary Material).

Insert Figure 1.

The three most common types of MSK problems in this population are low back pain (33.6%), lower limb (9%), and upper limb problems (9%). Figure 1b shows that, within the subgroup with absences due to MSK, low back pain had the highest percent impact on total number of working days lost (26.8%), followed by upper limb problems (12.3%), and lower limb problems (9.1%). Mean absence duration within the MSK-related absences ranged from 17 days due to neck problems to 40 days for upper limb. All other MSK conditions did not differ greatly in duration, and ranged from 23.4 to 34.1 days (Figure S1b in the Supplementary Material).

The three most common types of MH problems amongst those in the EASY population are stress (64.8%), anxiety (15.9%), and depression (13.7%). The job categories with the highest cases of MH-related absences are nursing/midwifery (55.3%), and administrative services (18.8%). Figure 1c demonstrates that stress accounts for the largest percent of working days lost (62.7%), followed by

For peer review only - http://bmjopen.bmj.com/site/about/guidelines.xhtml

depression (18.9%) and anxiety (14.6%). There was no significant change year on year in mean duration of MH-related absences (Figure S1c in the Supplementary Material), with depression resulting in the longest absences in all years (overall mean duration of 72.1 days). Anxiety and stress related absences had a mean duration of 48.1 and 50.7 days, respectively.

Mean absence duration in years two, three, four, five, and six of the EASY service was compared to year one using linear regression for all causes of SA to examine potential significant changes over time, and then for each of the nine conditions (Figure S1a in the Supplementary Material). Compared to year one, mean absence duration for MSK cases in Year 2, Year 3 and Year 4 decreased significantly (Figure S1a in the Supplementary Material).; mean absence duration for gastrointestinal (GI) cases in years five and six decreased significantly; and mean absence duration for cold, cough, and flu cases in years two, five and six decreased significantly (Figure S1a in the Supplementary Material).

<u>Sickness absence duration & return to work</u>

Figure 2 shows the Kaplan Meier RTW curves for all sickness absence events minus MSK and MH related absences (Figure 2a), for the MSK-related absences (Figure 2b, and Figure S2 in Supplementary Material) and for the MH-related absences (Figure 2c, and Figure S3 in Supplementary Material). RTW for staff absent because of MH problems was much longer than all other causes of absences (Figure 2a). For example, 50% for staff absent from work due to a MH problem had returned to work by 35 days, whereas 50% of those with an absence due to an MSK condition or all other conditions had returned within 10 and 5 days (respectively) of their FDA. As shown in Figure 2b, there are significant differences in RTW duration by sub-conditions within absences due to a MSK condition. Upper limb conditions result in the longest absences (50% of staff RTW by 25 days) whereas lower back and neck problems, result in

For peer review only - http://bmjopen.bmj.com/site/about/guidelines.xhtml

the shortest absences (50% of staff RTW by 7 days for both conditions). For the other musculoskeletal conditions (knee, lower limb, shoulder and other), 50% of the population RTW (P_{50}) between 10 and 14 days. Mental health related absences are much longer (**Figure 2c**). Depression is the leading cause of longer SA events, with 50% of staff RTW by 53 days, followed by stress (50% of staff RTW by 34 days) and anxiety and Other mental health conditions (50% of staff RTW by 29 days).

Table 2. Multivariate cox regression RTW hazard ratios for all (minus MSK and MH), MSK and MH conditions*.

	**All Conditions (minus MSK & MH)**			**Musculoskeletal Conditions**			**Mental Health Conditions**		
	**HR**	**95% CI**	**P**	**HR**	**95% CI**	**P**	**HR**	**95% CI**	**P**
**Population**									
All EASY except MSK & MH	1			-	-	-	-	-	-
MSK	0.51	(0.49, 0.52)	0.000	-	-	-	-	-	-
MH	0.24	(0.23, 0.26)	0.000	-	-	-	-	-	-
**MSK condition**									
Lower back	-	-	-	1			-	-	-
Knee	-	-	-	0.84	(0.75, 0.95)	0.006	-	-	-
Lower limb	-	-	-	0.80	(0.73, 0.88)	0.000	-	-	-
Neck	-	-	-	1.16	(1.05, 1.29)	0.003	-	-	-
Shoulder	-	-	-	0.79	(0.69, 0.89)	0.000	-	-	-
Upper limb	-	-	-	0.61	(0.56, 0.68)	0.000	-	-	-
Other	-	-	-	0.73	(0.68, 0.78)	0.000	-	-	-
**MH condition**									
Depression	-	-	-	-	-	-	1		
Anxiety	-	-	-	-	-	-	1.63	(1.40, 1.89)	0.000
Stress	-	-	-	-	-	-	1.64	(1.41, 1.90)	0.000
Other	-	-	-	-	-	-	1.80	(1.43, 2.27)	0.000
**Gender**									
Male	1			1			1		
Female	0.94	(0.89, 0.94)	0.000	0.77	(0.71, 0.84)	0.000	1.07	(0.93, 1.23)	0.336
**Age**	0.99	(0.99, 0.99)	0.000	0.99	(0.99, 0.99)	0.000	0.99	(0.99, 1.00)	0.000
**Job category**									
Administrative services	1			1			1		
Allied Health Profession	1.10	(1.06, 1.13)	0.000	0.99	(0.88, 1.10)	0.797	1.18	(1.00, 1.38)	0.045

For peer review only - http://bmjopen.bmj.com/site/about/guidelines.xhtml

Healthcare Sciences	1.01	(0.96, 1.06)	0.642	1.09	(0.96, 1.25)	0.197	1.11	(0.90, 1.37)	0.330
Manager	1.11	(0.95, 1.31)	0.195	1.49	(0.86, 2.58)	0.156	1.46	(0.60, 3.55)	0.399
Medical & Dental	1.17	(1.11, 1.25)	0.000	0.99	(0.82, 1.20)	0.921	1.43	(1.00, 2.05)	0.048
Medical and Dental Support	0.96	(0.90, 1.03)	0.292	1.18	(0.95, 1.49)	0.141	0.98	(0.69, 1.38)	0.892
Nursing/Midwifery	0.84	(0.82, 0.86)	0.000	0.85	(0.79, 0.91)	0.000	1.01	(0.91, 1.11)	0.918
Other Therapeutic	1.23	(1.17, 1.29)	0.000	1.24	(1.06, 1.45)	0.007	1.21	(0.95, 1.55)	0.124
Personal and Social Care	0.95	(0.86, 1.05)	0.290	1.41	(1.08, 1.85)	0.013	0.84	(0.53, 1.36)	0.485
Support Services	0.85	(0.82, 0.89)	0.000	0.93	(0.84, 1.02)	0.129	1.23	(1.06, 1.42)	0.007
**Job type**									
Part time	1			1			1		
Full time	1.12	(1.10, 1.15)	0.000	1.05	(0.99, 1.10)	0.090	1.08	(1.00, 1.17)	0.048
**Year**									
May 08-Apr 09	1			1			1		
May 09-Apr 10	0.99	(0.95, 1.02)	0.466	1.13	(1.02, 1.24)	0.020	1.00	(0.86, 1.17)	0.978
May 10-Apr 11	0.95	(0.92, 0.99)	0.012	1.13	(1.03, 1.25)	0.013	0.99	(0.85, 1.15)	0.884
May 11-Apr 12	0.94	(0.91, 0.98)	0.002	1.11	(1.00, 1.22)	0.043	0.92	(0.79, 1.07)	0.273
May 12-Apr 13	0.92	(0.89, 0.96)	0.000	0.98	(0.88, 1.08)	0.624	0.91	(0.78, 1.06)	0.232
May 13-Apr 14	0.96	(0.93, 1.00)	0.036	1.08	(0.97, 1.19)	0.144	1.04	(0.89, 1.21)	0.617

*results by Day of Absence and Season presented in Supplementary Material Tables S1a-c

Insert Figure 2.

For peer review only - http://bmjopen.bmj.com/site/about/guidelines.xhtml

Multivariate Analysis for all EASY absences (minus MSK & MH), MSK and MH

Table 2 shows the results of the multivariate cox regression analysis to investigate the differences in return to work between the three main absence groups, gender, job title and year of absence. In terms of absence cause, in comparison to all of EASY (minus MSK & MH), time to RTW was 49% (95% CI .49-.52, P<0.0001) and 76% (95% CI .23-.66, P<0.0001) longer for absences due to MSK and MH conditions, respectively. No real differences were seen after adjusting for gender, age, job family, job type, cause of absence, day of absence, season of absence, and year of absence.

For MSK-related absences, staff absent due to neck problems took 16% longer to return to work (95% CI 1.05-1.29, P<0.0001) compared to those absent because of lower back problems, whereas employees who were absent because of all other MSK conditions (0.73; CI: 0.68, 0.78) had quicker RTW.

Anxiety, stress and all other mental health related absences resulted in significantly longer times to return to work, when compared to depression, and were 63%, 64% and 80% of longer duration, respectively (all p<0.001).

The analysis by gender demonstrated that for all absences (minus MSK & MH) and for MSK absences women exhibited longer RTW times than men (Table 2). No significant gender difference in RTW was observed for mental health conditions. From the data, it is not possible to ascertain, whether full-time or part-time working has any association with RTW, as hours and days of employment were not included in the database.

In terms of job category, for all conditions (minus MSK & MH) nurses had the longer duration to RTW followed by staff in 'support services' when compared to staff in the reference category of

For peer review only - http://bmjopen.bmj.com/site/about/guidelines.xhtml

'administrative services', (Table 2). Employees in 'other therapeutic services' and 'medical and dental' staff demonstrated 23% and 17%, respectively, shorter times to RTW than staff in 'administrative services'. For MSK related absence, the only significant differences to the reference category 'administrative services' was observed for 'nursing/midwifery' (HR: 0.85; Cis: 0.79, 0.91; p=0.000), and the 'other therapeutic' and 'personal and social care' categories. For mental health related absences, only 'personal and social care' staff took significantly longer to RTW compared to 'administrative' staff, whereas 'medical and dental' staff and 'allied health professionals' had 43% and 18%, respectively, shorter lengths of absence RTW than the 'administrative' job group.

The analysis by year of absence using Year 1 as the reference category, showed that staff take significantly longer to return to work in years 3-6 for all conditions (minus MSK & MH). When only MSK absences are examined, in years 2-4 staff time to RTW is significantly shorter than for year 1. There are no significant differences in time to RTW by year for staff absences due to a mental health condition. Additionally, when examining time to RTW by day of absence start on absences starting on a Tuesday took significantly longer to RTW (Tables S1a-c). No significant differences in time to RTW were observed for season in which the absence occurred (p>0.05) (Tables S1a-c).

Discussion

Summary of findings

We found significant differences in sickness absence duration by presenting condition in a population of healthcare workers. Mental health conditions, and depression specifically, accounted for the most

For peer review only - http://bmjopen.bmj.com/site/about/guidelines.xhtml

working days' absence. We also observed significant variations in duration for different musculoskeletal conditions. Upper limb disorders resulted in the longest sickness absence durations among these healthcare workers, with 50% of staff returning to work by day 25. Lower back and neck problems resulted in the shortest MSK-related absences (50% RTW by 7 days), whereas knee, lower limb, shoulder and other MSK conditions resulted in SA durations that were comparable to each other (50% RTW between 10-14 days).

Employees within the nursing and midwifery job category account for almost half (45.3%) of all sickness absence events recorded in the six year period, and over half of the MSK (52.5%) and MH-related (55.3%) absence events. Nurses and midwives also had longer times to RTW than every other job category when compared to the reference category of 'administrative services'. In terms of gender, no significant differences were detected in RTW between men and women who were absent from work because of mental health conditions, but for all other absence events women took longer to return to work than men.

<u>Research in context to previous studies</u>

The results of our study are in agreement with previous published work on the impact of musculoskeletal and mental health conditions on sickness absence and return to work [2 14 53-55]. A Danish cohort study aiming to identify prognostic factors associated with neck-shoulder pain resulting in long term sickness absence, found pain intensity and job characteristics, such as heavy physical workload, were significantly associated with longer absence duration [56]. Armijo-Olivo et al. (2016) also demonstrated that occupation and health condition were significant factors, among others, in the

For peer review only - http://bmjopen.bmj.com/site/about/guidelines.xhtml

rehabilitation process of people affected by MSK conditions [57]. Similarly for mental health conditions, previous research suggests that sickness absence associated with psychological ill-health tends to be higher among NHS healthcare workers than for other employment sectors in the UK [58]. This may be due to the pressured nature of the work, constant organizational changes, and the large workload [59], supporting the notion that organizational factors may contribute to the level of psychological ill health experienced by staff. These findings are also reflected in our study, where significant variations in absence duration are observed not only by certain MSK and MH conditions, but also by job categories - with nursing and midwifery staff experiencing the majority of and longest SA events. One study looking at job family and sickness absence in the healthcare sector, found that doctors have nine times lower rates of short-term sickness absence and four times lower rates of long-term sickness absence, while nurses had three times lower rates of short-term sickness absence when compared to other healthcare sector job families [44].

Strengths of the study

The latest Health and Safety Executive (HSE) report lists healthcare staff as one of the categories with the highest sickness absence rates, and mental health and MSK as leading causes [14]. This study is therefore particularly important as we are able to investigate in detail the durations of SA events by certain MSK and MH conditions in this population. The unique and rich EASY database [51] enables analysis of routine data collected in a systemic way across all job categories within the healthcare sector. While several studies have reviewed sickness absence in healthcare settings, these have been limited in several ways. For instance, they examine a narrow range of healthcare workers, mainly doctors and

For peer review only - http://bmjopen.bmj.com/site/about/guidelines.xhtml

nurses; or examine only broad categories of absence [39]. Thus, the size of the EASY database base, giving six full years of sickness absence and return to work data, and the range of variables collected - including demographic, job, start and end dates, self-reported conditions - are considerable strengths.

Study limitations

While the availability of such rich data on routine sickness absence is a major strength of this study, there are also some limitations to the data. This SA management service is only available Monday to Friday and therefore not all absences may have been recorded. To avoid any confounding we removed absences starting on a weekend. The cause of absence is self-reported by the employee when they call the service and not based on a clinical diagnosis, and co-morbidities - which may also impact on absence duration - are not collected [53].

Sickness absence is multi-causal and it is necessary to consider an individual's work and workplace environment. However, the lack of granularity in SA data recorded is often the barrier into investigating. Linking bespoke databases, such as the one in this study, to personnel data or having uniformity in the variables collected across health boards would allow to investigate the impact of operational (e.g. shift work) and organisational (region, structures) risk factors on sickness absence rates and duration.

As this health board has a unique SA management service, the results may not be representative of all healthcare workers. However, a national standard requires all health boards in Scotland to work towards a 4% or less sickness absence rate [60]. While NHS Lanarkshire (NHSL) did have a higher SA rate in early 2008, by the end of 2008 the SA rate had fallen to similar levels to the other health boards [18]. The latest data show that the NHSL SA rate of 5.20% is in line with the Scottish NHS average of 5.16% [60],

For peer review only - http://bmjopen.bmj.com/site/about/guidelines.xhtml

which demonstrates that currently there is not a significant difference in SA rates across Scotland.

Implications for policy and practice

Due to the financial and morale repercussions sickness absence in healthcare employees has not only on healthcare staff themselves, but also their patients and employers [31], it is an important focus of attention for healthcare management and public health policies and practice reform. Recent systematic reviews have evaluated the effectiveness of different types of SA interventions and have found that multidisciplinary interventions involving collaboration between employees, health practitioners and employers working to implement tailored modifications for the absentee were consistently more effective than generic non-tailored interventions targeted at all employees [13 61].

Conclusions

The results of this study further establish the need for occupational health, organizational and management interventions to address recognized individual and workplace stressors that can impact on sickness absence duration. Our results suggest that employees with upper limb disorders and depressive symptoms in particular, require more tailored interventions to assist them in the return to work process following a SA event. A great burden of work loss due to both musculoskeletal and mental health condition was observed for nurses and midwives.

This research is important in terms of improving the health and wellbeing of NHS staff but may also improve the quality of patient care, and subsequently public health. SA has far-ranging economic consequences for both employers and employees, as it simultaneously impacts on NHS

For peer review only

resources/service delivery and on people's earnings if the SA is prolonged. These findings give a deeper insight into the link between health, organizational, operational and sociodemographic factors influencing sickness absence. Understanding these relationships allows health providers to be better placed to plan the allocation of resources across their different competing stresses, build better models of sickness absence management and inform the development of tailored sickness absence interventions for NHS staff.

Acknowledgements

The authors would like to acknowledge Mark Kennedy, Keith Murray, Moyra Anderson, and Raymond Hamill for their support.

Author Contributions

ED developed the study and was the main author of the manuscript. AB and SS conducted the statistical analysis and contributed to the manuscript writing. JB, KH, SVP and EBM all reviewed and contributed to the manuscript.

Ethical Considerations

NHS Lanarkshire Research and Development (R&D) Management Approval (ref number: L11071).

For peer review only - http://bmjopen.bmj.com/site/about/guidelines.xhtml

Funding

This work was supported by the Medical Research Council partnership grant MC/PC/13027 to ED and funding from the UK MRC and Chief Scientist Office (MC_UU_12017/12; SPHSU12) to AB and KH.

Competing Interests

None

Data Sharing Statement

No additional data available

References

1. Henderson M, Glozier N, Elliott KH. Long term sickness absence - Is caused by common conditions and needs managing. *BMJ* 2005;330(7495):802-03. doi: 10.1136/bmj.330.7495.802
2. Ritchie KA, Macdonald EB, Gilmour WH, et al. Analysis of sickness absence among employees of four NHS trusts. *Occup Environ Med* 1999;56(10):702-08.
3. Wise J. Audit finds large variations in NHS staff sick days. *BMJ* 2011;342 doi: 10.1136/bmj.d953
4. Hussey L, Turner S, Thorley K, et al. Work-related sickness absence as reported by UK general practitioners. *Occupational Medicine* 2012;62(2):105-11. doi: 10.1093/occmed/kqr205
5. Pekkala J, Blomgren J, Pietiläinen O, et al. Occupational class differences in long sickness absence: a register-based study of 2.1 million Finnish women and men in 1996–2013. *BMJ Open* 2017;7(7) doi: 10.1136/bmjopen-2016-014325
6. Black C. Health at Work - an independent review of sickness absence. In: The Stationary Office, ed., 2011.
7. Vahtera J, Kivimaeki M. Reducing sickness absence in occupational settings. *Occup Environ Med* 2008;65(4):219-20. doi: 10.1136/oem.2007.036061
8. Fit for Work. http://www.fitforwork.org/. 2015
9. Health Works. *A review of the Scottish Government's Healthy Working Lives Strategy,* The Scottish Government, Edinburgh, Available from:

For peer review only - http://bmjopen.bmj.com/site/about/guidelines.xhtml

http://www.scotland.gov.uk/Publications/2009/12/11095000/0. In: Government TS, ed. Edinburgh, 2009.
10. Lammerts L, Schaafsma FG, Eikelenboom M, et al. Longitudinal Associations Between Biopsychosocial Factors and Sustainable Return to Work of Sick-Listed Workers with a Depressive or Anxiety Disorder. *Journal of occupational rehabilitation* 2016;26(1):70-9. doi: 10.1007/s10926-015-9588-z [published Online First: 2015/06/22]
11. van Vilsteren M, van Oostrom SH, de Vet HCW, et al. Workplace interventions to prevent work disability in workers on sick leave. *Cochrane Database of Systematic Reviews* 2015;Issue 10. Art. No.: CD006955.
12. Burton A, Kendall N, McCluskey S, et al. Telephonic support to facilitate return to work: what works, how, and when? In: Department for Work and Pensions, ed. London, 2013.
13. van Amelsvoort LG, Jansen NW, Kant I. Addressing long-term sickness absence: moving beyond disease, illness and work-related factors for effective prevention. *Scandinavian journal of work, environment & health* 2017(1):1-4. doi: 10.5271/sjweh.3605
14. HSE. Health and Safety Statistics, Annual report for Great Britain, 2014/2015 (Avaialbale from: http://www.hse.gov.uk/statistics/overall/hssh1415.pdf). In: Executive HaS, ed., 2015.
15. Boorman S. NHS Health and Well-being - Final Report. Leeds: Department of Health; 2009. [http://webarchive.nationalarchives.gov.uk/20130107105354/http://www.dh.gov.uk/prod_consum_dh/groups/dh_digitalassets/documents/digitalasset/dh_108907.pdf]. 2009
16. Suff P. Absence in the NHS. *Occup Health at Work* 2013;10(3):19-23.
17. ONS. Sickness absence in the labour market: february 2014. Available from: http://www.ons.gov.uk/employmentandlabourmarket/peopleinwork/labourproductivity/articles/sicknessabsenceinthelabourmarket/2014-02-25 2014 [
18. Brown J, Mackay D, Demou E, et al. The EASY (Early Access to Support for You) sickness absence service: a four-year evaluation of the impact on absenteeism. *Scand J Work Environ Health,* 2015;41(2):204-15. doi: 10.5271/sjweh.3480 [published Online First: 2015/01/28]
19. Gorman E, Yu S, Alamgir H. When healthcare workers get sick: exploring sickness absenteeism in British Columbia, Canada. *Work (Reading, Mass)* 2010;35(2):117-23. doi: 10.3233/wor-2010-0963 [published Online First: 2010/02/19]
20. Andersen LL, Clausen T, Burr H, et al. Threshold of Musculoskeletal Pain Intensity for Increased Risk of Long-Term Sickness Absence among Female Healthcare Workers in Eldercare. *Plos One* 2012;7(7) doi: 10.1371/journal.pone.0041287
21. Franche RL, Murray EJ, Ostry A, et al. Work disability prevention in rural areas: a focus on healthcare workers. *Rural and Remote Health* 2010;10(4)
22. Murphy IJ. Self-reported and employer-recorded sickness absence in doctors. *Occupational medicine (Oxford, England)* 2014;64(6):417-20. doi: 10.1093/occmed/kqu098
23. Roelen CAM, Mageroy N, van Rhenen W, et al. Low job satisfaction does not identify nurses at risk of future sickness absence: Results from a Norwegian cohort study. *International Journal of Nursing Studies* 2013;50(3):366-73. doi: 10.1016/j.ijnurstu.2012.09.012
24. van der Meer EWC, Boot CRL, van der Gulden JWJ, et al. Hand eczema among healthcare professionals in the Netherlands: prevalence, absenteeism, and presenteeism. *Contact Dermatitis* 2013;69(3):164-71. doi: 10.1111/cod.12099

For peer review only - http://bmjopen.bmj.com/site/about/guidelines.xhtml

25. Lalloo D, Demou E, Macdonald EB. Trends in NHS doctor and dentist referrals to occupational health. *Occupational medicine (Oxford, England)* 2016;66(4):316-9. doi: 10.1093/occmed/kqw016 [published Online First: 2016/03/02]
26. Alexis O. The management of sickness absence in the NHS. *British journal of nursing (Mark Allen Publishing)* 2011;20(22):1437-40, 42. doi: 10.12968/bjon.2011.20.22.1437 [published Online First: 2012/01/14]
27. Johnson CJ, Croghan E, Crawford J. The problem and management of sickness absence in the NHS: considerations for nurse managers. *Journal of nursing management* 2003;11(5):336-42. doi: 10.1046/j.1365-2834.2003.00404.x
28. McEwan IM. Absenteeism and sickness Absence. *Postgraduate Medical Journal* 1991;67(794):1067-71.
29. HSE. Health and safety at work. Summary statistics for Great Britain 2016. Available from: http://www.hse.gov.uk/statistics/overall/hssh1516.pdf?pdf=hssh1516. In: Executive HaS, ed., 2016.
30. HSE. Health and safety in the health and social care sector in Great Britain, 2014/15. Available from: http://www.hse.gov.uk/statistics/industry/healthservices/health.pdf?pdf=health. In: Executive HaS, ed., 2015.
31. Professor Sir Bruce Keogh KBE. Section 4.3. Workforce; "Review into the quality of care and treatment provided by 14 hospital trusts in England: overview report".

http://www.nhs.uk/nhsengland/bruce-keogh-review/documents/outcomes/keogh-review-finalreport.Pdf, 2013.
32. NICE. Depression: Treatment and Management of Depression in Adults. Clinical Guideline 90. London: Available at www.nice.org.uk. 2009
33. NICE. Anxiety: Management of Anxiety (Panic Disorder, With and Without Agoraphobia, and Generalised Anxiety Disorder) in Adults in Primary, Secondary and Community Care. Clinical Guideline 113. London: . Available at www.nice.org.uk. 2011
34. Wiberg M, Friberg E, Palmer E, et al. Sickness absence and subsequent disposable income: A population-based cohort study. *Scand J Public Health* 2015;43(4):432-40. doi: 10.1177/1403494815575341 [published Online First: 2015/03/31]
35. Hoy D, March L, Brooks P, et al. The global burden of low back pain: estimates from the Global Burden of Disease 2010 study. *Annals of the rheumatic diseases* 2014;73(6):968-74. doi: 10.1136/annrheumdis-2013-204428 [published Online First: 2014/03/26]
36. Wynne-Jones G, Cowen J, Jordan JL, et al. Absence from work and return to work in people with back pain: a systematic review and meta-analysis. *Occupational and Environmental Medicine* 2013 doi: 10.1136/oemed-2013-101571
37. Ariens GA, Bongers PM, Hoogendoorn WE, et al. High physical and psychosocial load at work and sickness absence due to neck pain. *Scandinavian journal of work, environment & health* 2002;28(4):222-31. [published Online First: 2002/08/30]
38. Dewa CS, Loong D, Bonato S. Work outcomes of sickness absence related to mental disorders: a systematic literature review. *BMJ open* 2014;4(7) doi: 10.1136/bmjopen-2014-005533

For peer review only - http://bmjopen.bmj.com/site/about/guidelines.xhtml

39. Michie S, Williams S. Reducing work related psychological ill health and sickness absence: a systematic literature review. *Occup Environ Med* 2003;60(1):3-9. [published Online First: 2002/12/25]
40. Judd LL, Schettler PJ, Solomon DA, et al. Psychosocial disability and work role function compared across the long-term course of bipolar I, bipolar II and unipolar major depressive disorders. *Journal of Affective Disorders* 2008;108(1-2):49-58. doi: 10.1016/j.jad.2007.06.014
41. Soderman E, Lisspers J, Sundin O. Depression as a predictor of return to work in patients with coronary artery disease. *Social science & medicine (1982)* 2003;56(1):193-202. [published Online First: 2002/11/19]
42. van den Brink RHS, Ormel J, Tiemens BG, et al. Predictability of the one-year course of depression and generalized anxiety in primary care. *General Hospital Psychiatry* 2002;24(3):156-63. doi: 10.1016/s0163-8343(02)00183-4
43. Munir F, Yarker J, Haslam C, et al. Returning to Work - The Role of Depression. Available from: http://www.mentalhealth.org.uk/publication-download/returning-work-role-depression. In: Foundation TMH, ed. Loughborough,, 2009.
44. Lim A, Chongsuvivatwong V, Geater A, et al. Influence of work type on sickness absence among personnel in a teaching hospital. *Journal of Occupational Health* 2002;44(4):254-63. doi: 10.1539/joh.44.254
45. Sandanger I, Nygard JF, Brage S, et al. Relation between health problems and sickness absence: gender and age differences--a comparison of low-back pain, psychiatric disorders, and injuries. *Scand J Public Health* 2000;28(4):244-52. [published Online First: 2001/03/03]
46. Krane L, Johnsen R, Fleten N, et al. Sickness absence patterns and trends in the health care sector: 5-year monitoring of female municipal employees in the health and care sectors in Norway and Denmark. *Human Resources for Health* 2014;12 doi: 10.1186/1478-4491-12-37
47. Kristensen TR, Jensen SM, Kreiner S, et al. Socioeconomic status and duration and pattern of sickness absence. A 1-year follow-up study of 2331 hospital employees. *BMC public health* 2010;10 doi: 10.1186/1471-2458-10-643
48. d'Errico A, Costa G. Socio-demographic and work-related risk factors for medium- and long-term sickness absence among Italian workers. *European journal of public health* 2012;22(5):683-8. doi: 10.1093/eurpub/ckr140 [published Online First: 2011/12/14]
49. MacEachen E, Clarke J, Franche R-L, et al. Systematic review of the qualitative literature on return to work after injury. *Scand J Work Environ Health* 2006;32(4):257-69.
50. Macdonald EB, Asanati K. Absence from work. *Contemporary Social Science* 2016;11(1):30-39. doi: 10.1080/21582041.2016.1246749
51. Demou E, Brown J, Sanati K, et al. A novel approach to early sickness absence management: The EASY (Early Access to Support for You) way. *Work (Reading, Mass)* 2015;53(3):597-608. doi: 10.3233/wor-152137 [published Online First: 2015/09/28]
52. Engel GL. Need for a new medical model - Challenge for biomedicine. *Science* 1977;196(4286):129-36. doi: 10.1126/science.847460
53. Ervasti J, Mattila-Holappa P, Joensuu M, et al. Predictors of Depression and Musculoskeletal Disorder Related Work Disability Among Young, Middle-Aged, and Aging Employees. *Journal of Occupational and Environmental Medicine* 2017;59(1):114-19. doi: 10.1097/jom.0000000000000921

For peer review only - http://bmjopen.bmj.com/site/about/guidelines.xhtml

54. Lotters F, Burdorf A. Prognostic factors for duration of sickness absence due to musculoskeletal disorders. *The Clinical journal of pain* 2006;22(2):212-21. [published Online First: 2006/01/24]
55. Nieuwenhuijsen K, Verbeek JH, de Boer A, et al. Predicting the duration of sickness absence for patients with common mental disorders in occupational health care. *Scandinavian Journal of Work Environment & Health* 2006;32(1):67-74.
56. Holtermann A, Hansen JV, Burr H, et al. Prognostic factors for long-term sickness absence among employees with neck-shoulder and low-back pain. *Scandinavian journal of work, environment & health* 2010;36(1):34-41. [published Online First: 2009/12/08]
57. Armijo-Olivo S, Woodhouse LJ, Steenstra IA, et al. Predictive value of the DASH tool for predicting return to work of injured workers with musculoskeletal disorders of the upper extremity. *Occupational and Environmental Medicine* 2016 doi: 10.1136/oemed-2016-103791
58. Williams S, Michie S, Pattani S. Improving the health of the NHS workforce. London: Nuffield Trust; http://discovery.ucl.ac.uk/16094/. 1998
59. Cox T. The nature and measurement of stress. *Ergonomics* 1985;28(8):1155-63. doi: 10.1080/00140138508963238 [published Online First: 1985/08/01]
60. ISD NHS. NHSScotland Workforce Information. Quarterly update of Staff in Post, Vacancies and Turnover at 31 March 2016. Available from: https://www.isdscotland.org/Health-Topics/Workforce/Publications/2016-06-07/2016-06-07-Workforce-Report.pdf. In: Scotland NNS, ed., 2016.
61. Durand MJ, Corbiere M, Coutu MF, et al. A review of best work-absence management and return-to-work practices for workers with musculoskeletal or common mental disorders. *Work (Reading, Mass)* 2014;48(4):579-89. doi: 10.3233/wor-141914 [published Online First: 2014/07/06]

Figure Captions:

Figure 1. Cause of sickness absence (% of total number of events & % Impact) for (a) all conditions, (b) MSK conditions**, and (c) mental health conditions***

*Impact is estimated as percent impact and calculated by number of events times the average condition-specific absence duration divided by the sum of impact for all causes times 100
**Number of absences due to 'Hip' were to small and grouped into 'Other' category
*** Number of absences due to 'Bipolar Disorder', 'Schizophrenia', 'Panic Attacks' and 'Self harm' were to small and grouped into 'Other' category

Figure 2. a. Return to Work curves for (top) all absences with 95%CI; (bottom-left) b. MSK-related absences by MSK condition; and (bottom-right) c. MH-related absences by MH condition (for Survival

For peer review only - http://bmjopen.bmj.com/site/about/guidelines.xhtml

curves with 95%Cis for MSK and MH conditions see Supplementary Material Figures S2 and S3

For peer review only

For peer review only - http://bmjopen.bmj.com/site/about/guidelines.xhtml

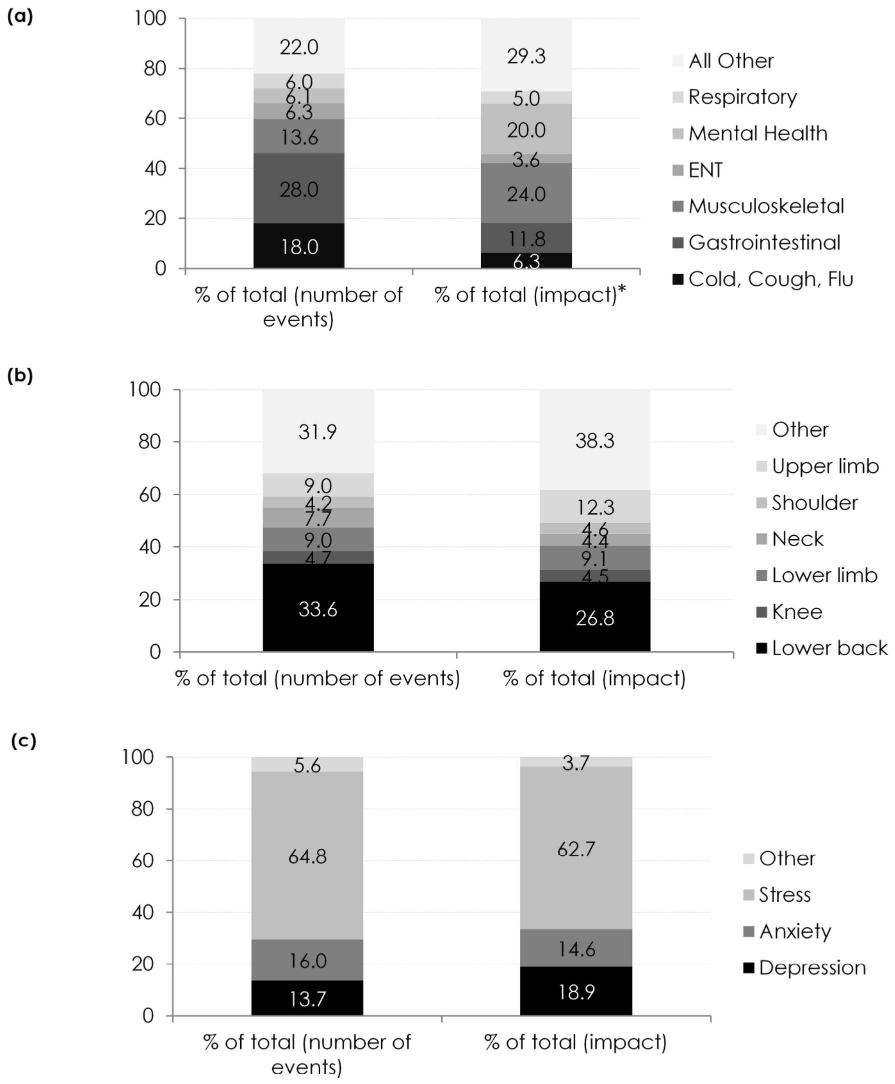

Figure 1. Cause of sickness absence (% of total number of events & % Impact) for (a) all conditions, (b) MSK conditions**, and (c) mental health conditions***
*Impact is estimated as percent impact and calculated by number of events times the average condition-specific absence duration divided by the sum of impact for all causes times 100
**Number of absences due to 'Hip' were to small and grouped into 'Other' category
*** Number of absences due to 'Bipolar Disorder', 'Schizophrenia', 'Panic Attacks' and 'Self harm' were to small and grouped into 'Other' category

225x300mm (300 x 300 DPI)

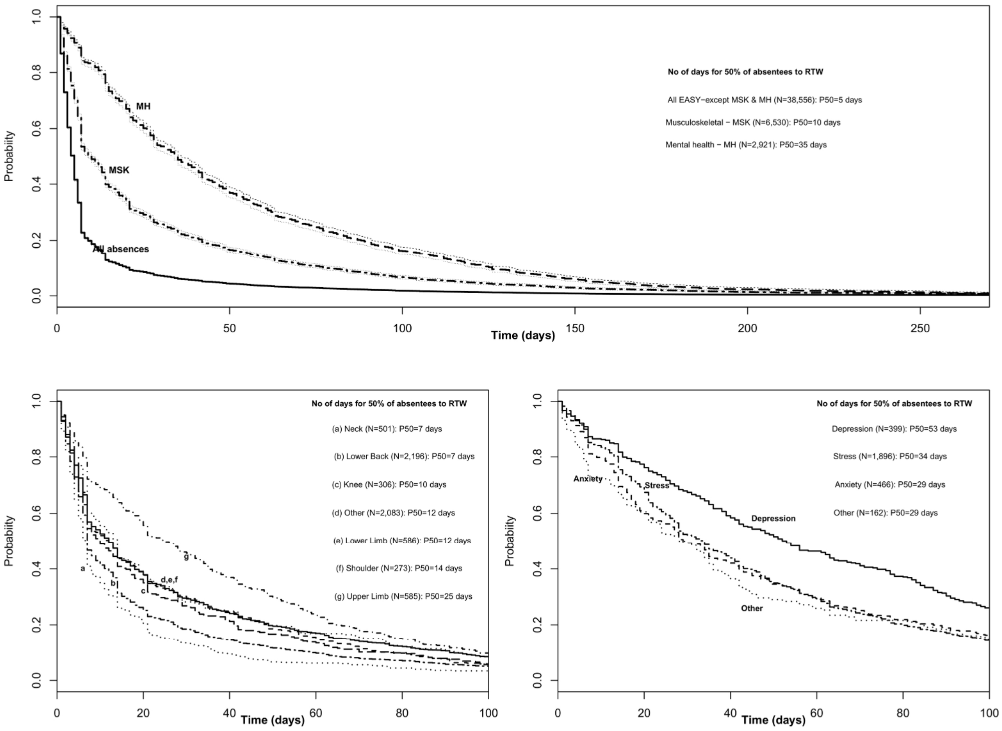

Caption : Figure 2. a. Return to Work curves for (top) all absences with 95%CI; (bottom-left) b. MSK-related absences by MSK condition; and (bottom-right) c. MH-related absences by MH condition (for Survival curves with 95%Cis for MSK and MH conditions see Supplementary Material Figures S2 and S3

233x179mm (300 x 300 DPI)

Evaluating Sickness Absence Duration by Musculoskeletal and Mental Health Issues. A retrospective study of Scottish Healthcare Workers

[Supplementary material]

Evangelia Demou PhD[1], Shanley Smith[2], Abita Bhaskar[1], Judith Brown PhD[2], Kate Hunt[1], Sergio Vargas-Prada MD[3,4], Ewan B Macdonald MD[2]

1. MRC/CSO Social and Public Health Sciences Unit, University of Glasgow, Glasgow, G2 3QB, United Kingdom

2. Healthy Working Lives Group, University of Glasgow, Glasgow, G12 8RZ, United Kingdom

3. Center for Research in Occupational Health (CiSAL), Universitat Pompeu Fabra, Barcelona, Spain

4. Unidad Central de Contingencias Comunes (U3C), ASEPEYO, Barcelona, Spain.

Running title Sickness Absence Duration in Healthcare Workers

Corresponding Author

Dr Evangelia Demou

Research Fellow

MRC/CSO Social and Public Health Sciences Unit

University of Glasgow

200 Renfield Street, Glasgow, G2 3QB, UK

Tel: 0141 353 7580

For peer review only - http://bmjopen.bmj.com/site/about/guidelines.xhtml

Figure S1a. Mean duration (in days) of absence by cause of sickness

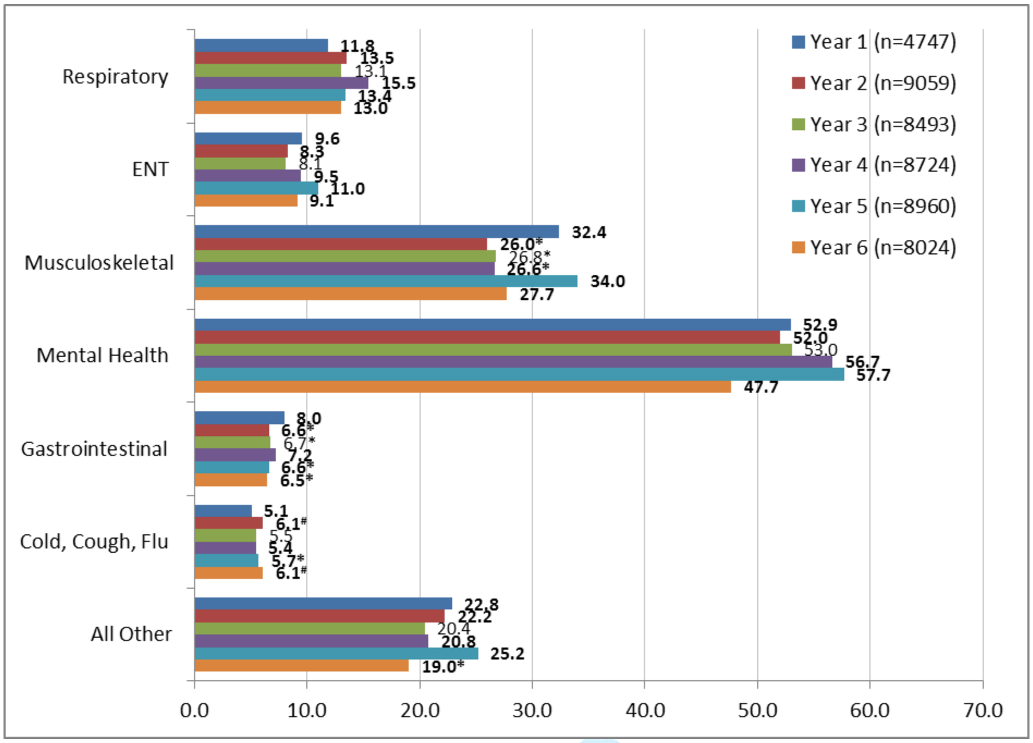

Figure S1b. Mean duration (in days) of absence by MSK cause of sickness

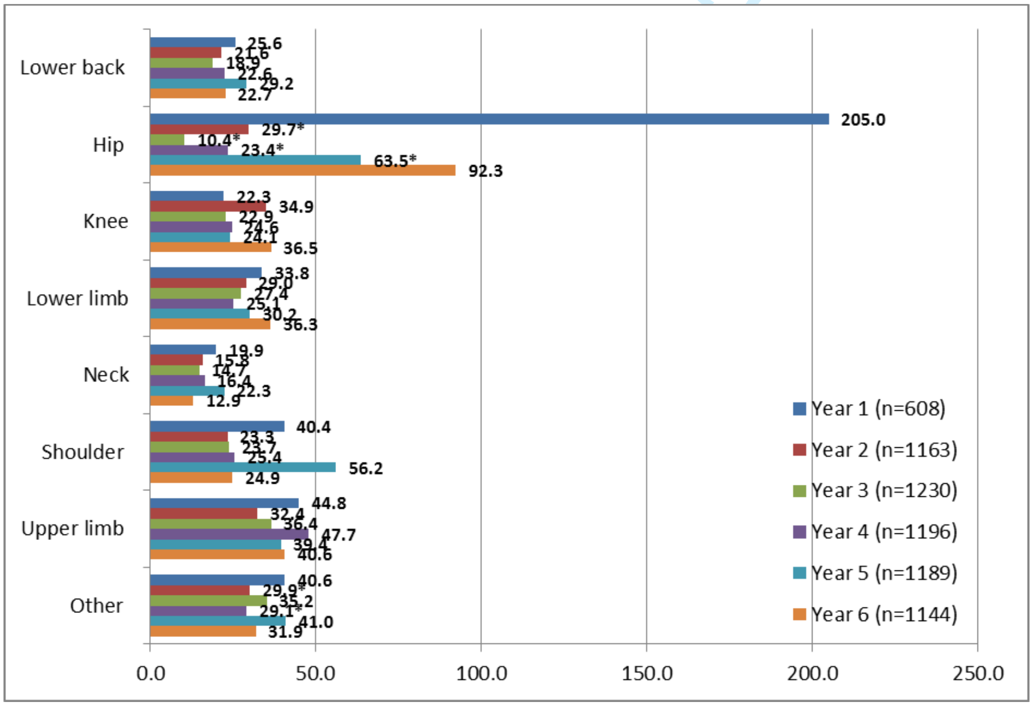

For peer review only - http://bmjopen.bmj.com/site/about/guidelines.xhtml

Figure S1c. Mean duration (in days) of absence by MH cause of sickness

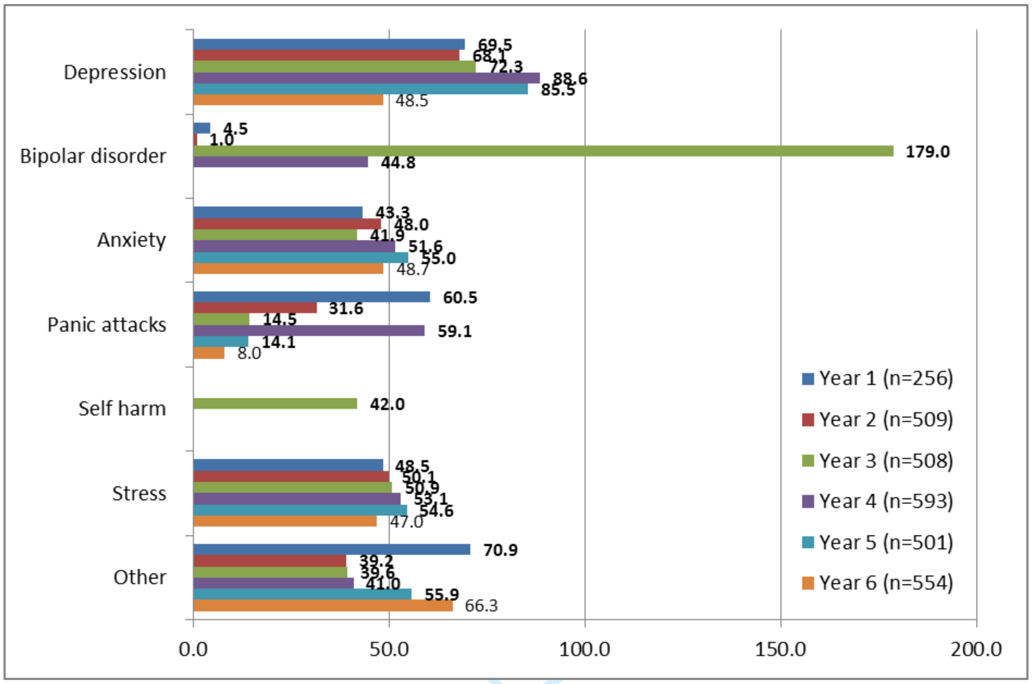

Table S1a. Multivariate cox regression hazard ratios for full population, adjusted for sex, age, job category, job type, day of first absence, season of absence and year of absence.

All	HR	95% CI	P
Day of absence			
Mon	1		
Tues	1.07	(1.04, 1.10)	0.000
Wed	1.02	(0.99, 1.05)	0.155
Thurs	1.02	(0.99, 1.05)	0.263
Fri	0.96	(0.93, 1.00)	0.026
Season			
Spring	1		
Summer	1.00	(0.97, 1.03)	0.960
Autumn	1.01	(0.98, 1.03)	0.675
Winter	1.01	(0.99, 1.04)	0.238

r review only

For peer review only - http://bmjopen.bmj.com/site/about/guidelines.xhtml

Table S1b. Multivariate cox regression hazard ratios for staff with musculoskeletal conditions, adjusted for sex, age, job category, job type, day of first absence, season of absence and year of absence.

Musculoskeletal	HR	95% CI	P
Day of absence			
Mon	1		
Tues	1.06	(0.99, 1.14)	0.088
Wed	1.07	(1.00, 1.15)	0.063
Thurs	1.08	(1.01, 1.17)	0.031
Fri	1.01	(0.93, 1.10)	0.753
Season			
Spring	1		
Summer	1.01	(0.94, 1.08)	0.793
Autumn	1.01	(0.94, 1.08)	0.778
Winter	1.01	(0.94, 1.08)	0.772

Table S1c. Multivariate cox regression hazard ratios for staff with mental health conditions, adjusted for sex, age, job category, job type, day of first absence, season of absence and year of absence.

Mental health	HR	95% CI	P
Job type			
Part time	1		
Full time	1.08	(1.00, 1.17)	0.048
Day of absence			
Mon	1		
Tues	1.03	(0.93, 1.15)	0.540
Wed	1.00	(0.90, 1.11)	0.993
Thurs	0.99	(0.89, 1.11)	0.917
Fri	0.90	(0.80, 1.02)	0.089
Season			
Spring	1		
Summer	1.03	(0.92, 1.14)	0.649
Autumn	1.06	(0.96, 1.18)	0.235
Winter	1.02	(0.92, 1.13)	0.689

For peer review only - http://bmjopen.bmj.com/site/about/guidelines.xhtml

For peer review only

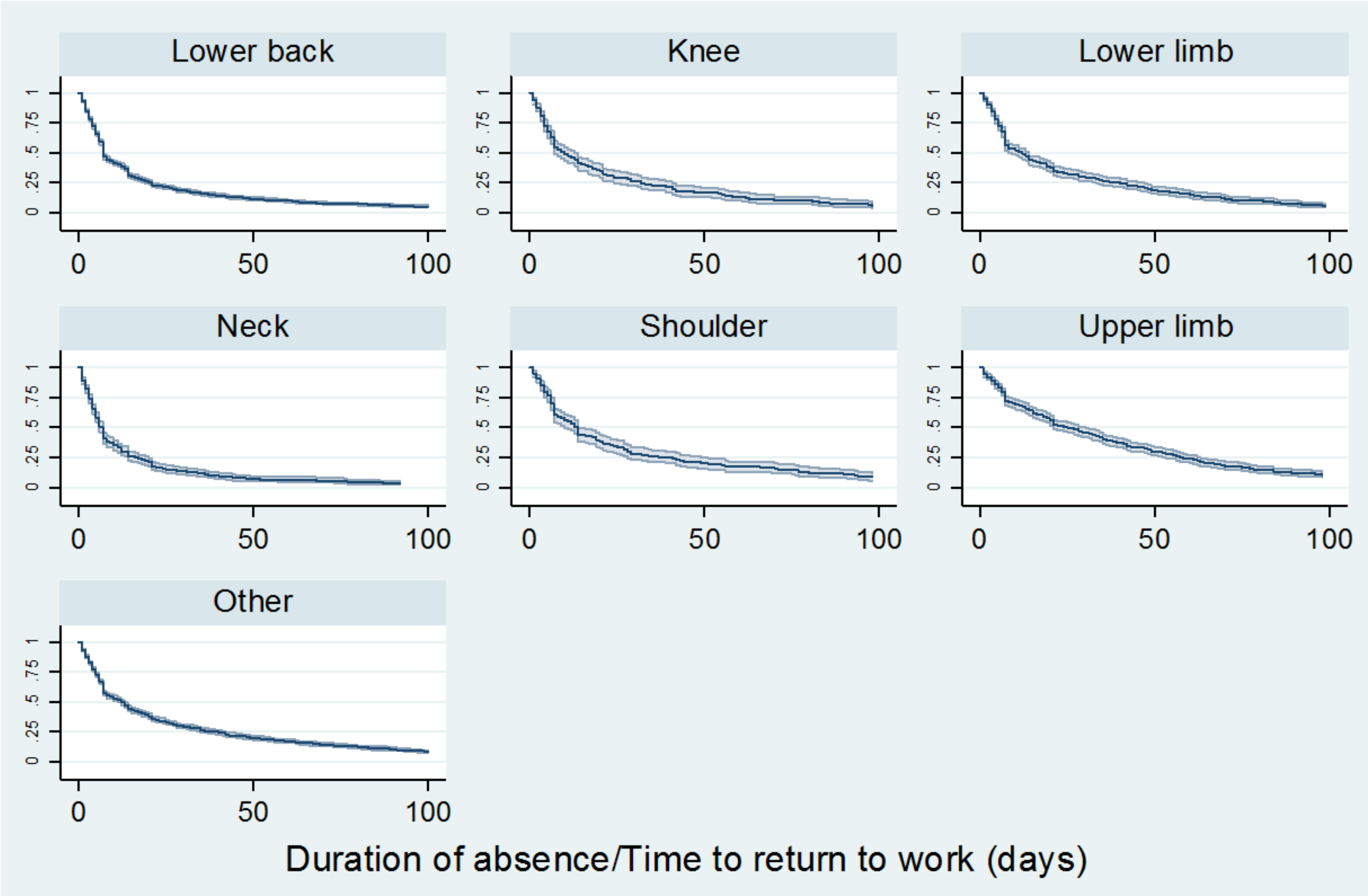

Figure S2. Return to Work curves for all MSK-related absences by MSK condition with 95%CI

For peer review only - http://bmjopen.bmj.com/site/about/guidelines.xhtml

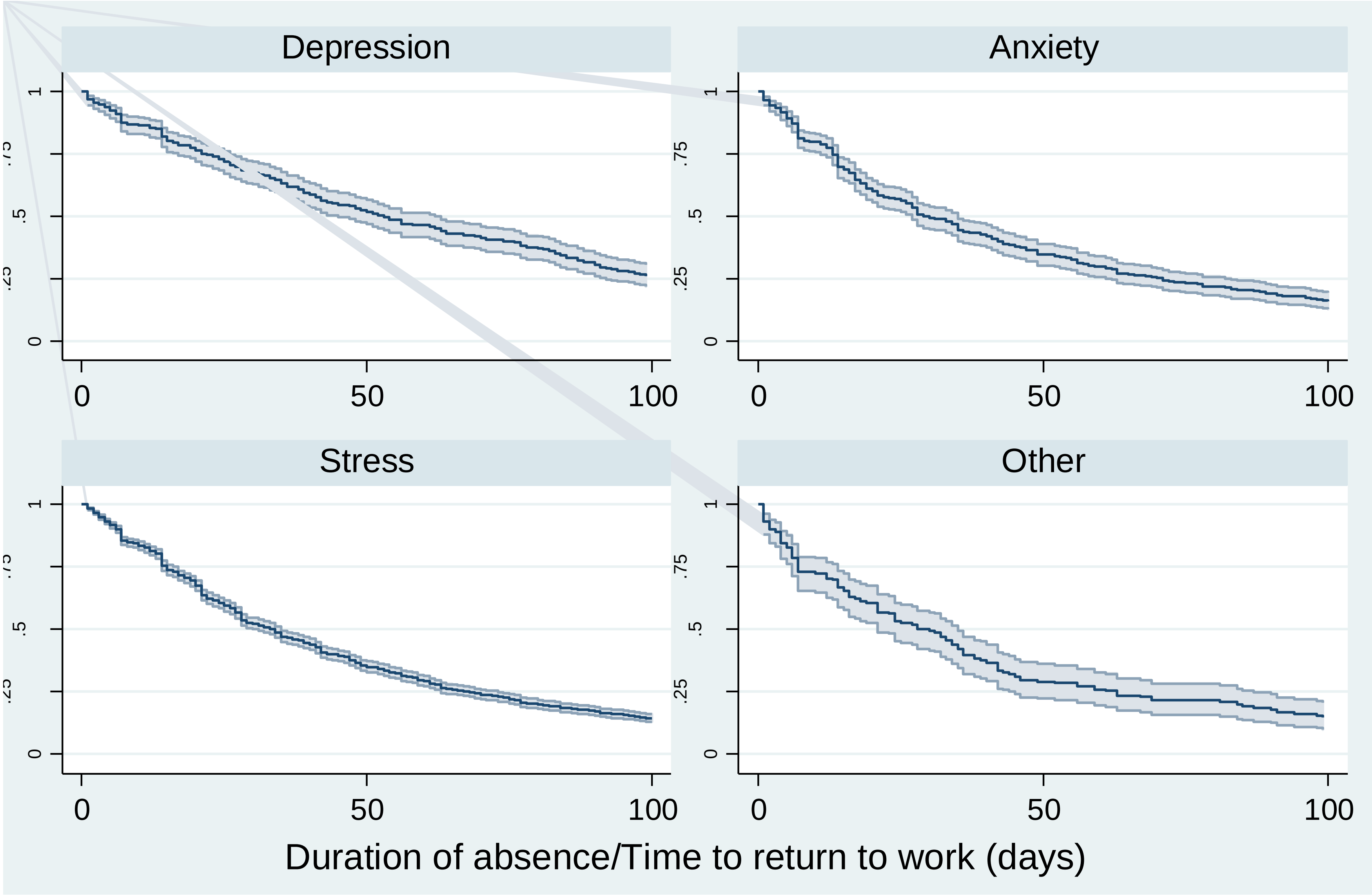

Figure S3. Return to Work curves for all MH-related absences by MH condition with 95%CI

For peer review only - http://bmjopen.bmj.com/site/about/guidelines.xhtml

STROBE Statement—checklist of items that should be included in reports of observational studies

	Item No.	Recommendation	Page No.	Relevant text from manuscript
**Title and abstract**	1	(*a*) Indicate the study's design with a commonly used term in the title or the abstract	2	This cross-sectional study…..
		(*b*) Provide in the abstract an informative and balanced summary of what was done and what was found	2	Survival analyses and Cox's proportional hazards models were used to estimate SA duration……
**Introduction**				
Background/rationale	2	Explain the scientific background and rationale for the investigation being reported	4-7	
Objectives	3	State specific objectives, including any prespecified hypotheses	7	Study aim
**Methods**				
Study design	4	Present key elements of study design early in the paper	8	
Setting	5	Describe the setting, locations, and relevant dates, including periods of recruitment, exposure, follow-up, and data collection	8-9	
Participants	6	(*a*) *Cohort study*—Give the eligibility criteria, and the sources and methods of selection of participants. Describe methods of follow-up<br>*Case-control study*—Give the eligibility criteria, and the sources and methods of case ascertainment and control selection. Give the rationale for the choice of cases and controls<br>*Cross-sectional study*—Give the eligibility criteria, and the sources and methods of selection of participants	8-10	
		(*b*) *Cohort study*—For matched studies, give matching criteria and number of exposed and unexposed<br>*Case-control study*—For matched studies, give matching criteria and the number of controls per case		
Variables	7	Clearly define all outcomes, exposures, predictors, potential confounders, and effect modifiers. Give diagnostic criteria, if applicable	9-10	
Data sources/ measurement	8*	For each variable of interest, give sources of data and details of methods of assessment (measurement). Describe comparability of assessment methods if there is more than one group	*9-10*	
Bias	9	Describe any efforts to address potential sources of bias	9-10	multivariate model controlled for several occupational and individual
Study size	10	Explain how the study size was arrived at	8-9	

Continued on next page

For peer review only - http://bmjopen.bmj.com/site/about/guidelines.xhtml

Quantitative variables	11	Explain how quantitative variables were handled in the analyses. If applicable, describe which groupings were chosen and why	9-10	
Statistical methods	12	(*a*) Describe all statistical methods, including those used to control for confounding	9-10	
		(*b*) Describe any methods used to examine subgroups and interactions	8-10	
		(*c*) Explain how missing data were addressed	8-9	
		(*d*) *Cohort study*—If applicable, explain how loss to follow-up was addressed<br>*Case-control study*—If applicable, explain how matching of cases and controls was addressed<br>*Cross-sectional study*—If applicable, describe analytical methods taking account of sampling strategy	9	
		(*e*) Describe any sensitivity analyses		
**Results**				
Participants	13*	(a) Report numbers of individuals at each stage of study—eg numbers potentially eligible, examined for eligibility, confirmed eligible, included in the study, completing follow-up, and analysed	10-11	
		(b) Give reasons for non-participation at each stage	na	
		(c) Consider use of a flow diagram	na	
Descriptive data	14*	(a) Give characteristics of study participants (eg demographic, clinical, social) and information on exposures and potential confounders	11	Table 1
		(b) Indicate number of participants with missing data for each variable of interest	na	
		(c) *Cohort study*—Summarise follow-up time (eg, average and total amount)		
Outcome data	15*	*Cohort study*—Report numbers of outcome events or summary measures over time		
		Case-control study—Report numbers in each exposure category, or summary measures of exposure		
		Cross-sectional study—Report numbers of outcome events or summary measures	*11& 14*	*Tables 1 &2*
Main results	16	(*a*) Give unadjusted estimates and, if applicable, confounder-adjusted estimates and their precision (eg, 95% confidence interval). Make clear which confounders were adjusted for and why they were included	14 & 16	
		(*b*) Report category boundaries when continuous variables were categorized		
		(*c*) If relevant, consider translating estimates of relative risk into absolute risk for a meaningful time period		

Continued on next page

For peer review only - http://bmjopen.bmj.com/site/about/guidelines.xhtml

Other analyses	17	Report other analyses done—eg analyses of subgroups and interactions, and sensitivity analyses	17	The analysis by year of absence…
**Discussion**				
Key results	18	Summarise key results with reference to study objectives	17-18	Summary of findings section
Limitations	19	Discuss limitations of the study, taking into account sources of potential bias or imprecision. Discuss both direction and magnitude of any potential bias	20	Study limitations section
Interpretation	20	Give a cautious overall interpretation of results considering objectives, limitations, multiplicity of analyses, results from similar studies, and other relevant evidence	17-20	
Generalisability	21	Discuss the generalisability (external validity) of the study results	20	The latest data show that the NHSL SA rate is in line with the Scottish NHS average
**Other information**				
Funding	22	Give the source of funding and the role of the funders for the present study and, if applicable, for the original study on which the present article is based	22	Funding section

*Give information separately for cases and controls in case-control studies and, if applicable, for exposed and unexposed groups in cohort and cross-sectional studies.

Note: An Explanation and Elaboration article discusses each checklist item and gives methodological background and published examples of transparent reporting. The STROBE checklist is best used in conjunction with this article (freely available on the Web sites of PLoS Medicine at http://www.plosmedicine.org/, Annals of Internal Medicine at http://www.annals.org/, and Epidemiology at http://www.epidem.com/). Information on the STROBE Initiative is available at www.strobe-statement.org.

For peer review only

For peer review only - http://bmjopen.bmj.com/site/about/guidelines.xhtml

BMJ Open

Evaluating Sickness Absence Duration by Musculoskeletal and Mental Health Issues. A retrospective cohort study of Scottish Healthcare Workers.

Journal:	*BMJ Open*
Manuscript ID	bmjopen-2017-018085.R2
Article Type:	Research
Date Submitted by the Author:	15-Nov-2017

Complete List of Authors:	Demou, Evangelia; University of Glasgow, MRC/CSO Social and Public Health Sciences Unit; University of Glasgow, Public Health, Institute of Health and Wellbeing<br>Smith, Shanley; University of Glasgow, Public Health, Institute of Health and Wellbeing<br>Bhaskar, Abita; University of Glasgow, MRC/CSO Social and Public Health Sciences Unit<br>Mackay, Daniel; University of Glasgow, Public Health, Institute of Health and Wellbeing<br>Brown, Judith; University of Glasgow, Public Health, Institute of health and Wellbeing<br>Hunt, Kate; University of Glasgow, MRC/CSO Social and Public Health Sciences Unit<br>Vargas, Sergio; Pompeu Fabra University, Center for Research in Occupational Health (CiSAL)<br>Macdonald, Ewan; The University of Glasgow, Healthy working lives group

**Primary Subject Heading**:	Occupational and environmental medicine
Secondary Subject Heading:	Epidemiology, Health policy, Mental health, Occupational and environmental medicine, Public health
Keywords:	MENTAL HEALTH, healthcare worker, anxiety, depression, Musculoskeletal disorders < ORTHOPAEDIC & TRAUMA SURGERY, return to work

SCHOLARONE™
Manuscripts

Evaluating Sickness Absence Duration by Musculoskeletal and Mental Health Issues. A retrospective cohort study of Scottish Healthcare Workers.

Evangelia Demou PhD[1], Shanley Smith[2], Abita Bhaskar[1], Daniel F Mackay[2], Judith Brown PhD[2], Kate Hunt PhD[1], Sergio Vargas-Prada MD PhD[3,4,5,6], Ewan B Macdonald MD[2]

1. MRC/CSO Social and Public Health Sciences Unit, University of Glasgow, Glasgow, G2 3QB, United Kingdom

2. Public Health, Institute of Health and Wellbeing, University of Glasgow, Glasgow, G12 8RZ, United Kingdom

3. Center for Research in Occupational Health (CiSAL), Universitat Pompeu Fabra, Barcelona, Spain

4. Unidad Central de Contingencias Comunes (U3C), ASEPEYO, Barcelona, Spain.

5. CIBER Epidemiología y Salud Pública (CIBERESP), Spain.

6. IMIM (Hospital del Mar Research Institute), Barcelona, Spain

Running title Sickness Absence Duration in Healthcare Workers

Word count: 4,375

Number of figures: 2

Number of tables: 2

Key terms work; return to work; nurses; doctors, back pain, depression, anxiety, healthcare worker

Corresponding Author
Dr Evangelia Demou
Research Fellow
MRC/CSO Social and Public Health Sciences Unit
University of Glasgow
200 Renfield Street, Glasgow, G2 3QB, UK
Email: evangelia.demou@glasgow.ac.uk
Tel: 0141 353 7580

For peer review only

For peer review only - http://bmjopen.bmj.com/site/about/guidelines.xhtml

Abstract

Objectives: Sickness absence (SA) among healthcare workers is associated with occupational and non-occupational risk factors, and impacts employee health, healthcare delivery and patient health. At the same time, it is one of the employment sectors with the highest rates of work-related ill-health in the UK. Musculoskeletal (MSK) and mental health (MH) issues are leading causes of SA, but there is a lack of research on how certain MSK/MH conditions impact on SA duration. The study aim is to determine differences in SA duration by MH and MSK disorders in healthcare employees.

Methods: Survival analyses were used to estimate SA duration due to MSK and MH problems over six years and Cox's proportional hazards models to determine the hazard ratios of returning-to-work, using a bespoke Scottish health board database with over 53,000 SA events. SA duration and time to return-to-work were estimated for employees by age, gender, job and health conditions.

Results: MSK and MH conditions accounted for 27% and 6%, respectively, of all SA events and 23.7% and 19.5% of all days lost. Average SA duration was 43.5 days for MSK and 53.9 days for MH conditions. For MSK conditions, employees with low back pain had the fastest return-to-work (median P_{50}: 7 days), while employees absent due to depression took the longest (P_{50}: 54 days). The most influential socio-demographic variables affecting return-to-work were age, gender, and job category.

Conclusions: Using a unique and rich database we found significant differences in SA duration by presenting condition in healthcare workers. Mental health conditions, and depression specifically, accounted for the most working days' absence. Significant variations in duration were also observed for musculoskeletal conditions. Our findings can inform public health practitioners and healthcare managers of the most significant factors impacting MSK and MH-related SA to develop and implement tailored and

For peer review only - http://bmjopen.bmj.com/site/about/guidelines.xhtml

targeted workplace interventions.

Article summary - Strengths and limitations of this study

- Healthcare is one of the employment sectors with significantly higher rates of sickness absence (SA), impacting on employee health, healthcare delivery and patient health. This study uses a unique, data-rich and bespoke database that allows for detailed assessment of the impact of MSK and MH conditions on absence duration and return to work for healthcare employees of a Scottish health board.
- Cox's proportional hazards models applying time varying coefficients were used to estimate SA duration due to musculoskeletal (MSK) and mental health (MH) problems over six years (N=48,007 unique SA events).
- The size and granularity of the database, giving six full years of sickness absence and return to work data, and the range of variables collected - including demographic, job, SA start and end dates, self-reported conditions- allowed for the investigation of SA duration and time to return-to-work for healthcare employees by age, gender, job and health conditions.
- SA data are from a single large health board (c.12,000 employees) which limits the degree of generalizability. Also as SA absence is multi-causal and it is necessary to consider an individual's work and workplace environment, there are a number of variables that are not collected as part of the service (e.g. operational and organizational variables) which could potentially be important risk factors for SA and RTW in this population.

For peer review only - http://bmjopen.bmj.com/site/about/guidelines.xhtml

Introduction

Sickness absence (SA) is a significant public health burden on government, employees, employers, and public resources [1-5]. This is due to loss of productivity, increased workload on other staff, as well as resources spent to cover incapacity to work and restore health [2 6]. SA data are increasingly being used as a measure of ill-health [7]. The public health burden of sickness absence is recognized and governments and employers are developing policies toward providing support to employers and employees in improving health, reducing sickness absence rates, and improving return to work (RTW) times [8-12].

Multiple factors need to be considered to effectively manage SA through policy and practice [13]. Sickness absence rates vary by sector and employer, with the healthcare sector exhibiting one of the highest rates[14]. Additionally, within the health service, variations in SA rates depend on region, job category and salary grade, among other factors [3 15 16]. Cause of SA can have a major impact on duration and overall costs. Long term sickness absence (LTSA), although only responsible for a small percentage of SA *events*, makes up approximately 75% of absence *costs*, with musculoskeletal (MSK) and mental health (MH) disorders being two of the leading causes of LTSA [2 3 14 17 18].

<u>Healthcare Employees and Sickness Absence</u>

The healthcare sector is a diverse entity, which presents a unique set of challenges in relation to SA duration [19]. Studies show that healthcare workers experience high exposures to both occupational and non-occupational risk factors, negatively impacting on SA events [19-24]. Healthcare employee absence leads to increases in the risks to quality of patient care, workload stress of colleagues, and employers'

For peer review only - http://bmjopen.bmj.com/site/about/guidelines.xhtml

staffing costs [19 25-28]. The most recent 2016 figures from the Health and safety Executive (HSE) report the healthcare industry as one of the employment sectors with the highest rates of work-related illness [29]. Ill health in the health and social care sector leads to around 4.8 million working days lost with the majority due to mental health disorders, followed by work-related musculoskeletal disorders [30]. A study examining quality of care and treatment in 14 English hospital trusts found a positive association between the in-patient to staff ratio and high hospital standardized mortality ratio (HSMR) scores [31] and also identified insufficient nursing establishments and poor staffing levels on night shifts and weekends, partially due to high SA rates [31].

Impact of Sickness Absence Cause and Duration

Certain health conditions result in longer periods of SA [14]. These thus have large potential interest as the focus of workplace interventions, due to the greater impact longer SA spells have on individuals, colleagues and employers [32 33]. There are potential long term effects on individuals who experience particular types of SA events; one Swedish cohort study, for example, found an association between long term absence and lower disposable income in the 2-6 years following the absence event [34]. A previous study investigating the effectiveness of an early SA management service in a Scottish Health Board, found that although the leading causes of SA were gastrointestinal problems, followed by cold/cough/flu, the greatest impact on total number of days lost was due to musculoskeletal (MSK) and mental health (MH) problems [18].

MSK related absence

For peer review only - http://bmjopen.bmj.com/site/about/guidelines.xhtml

Health Response UK reports musculoskeletal injury as the leading cause of absenteeism and related cost to UK Industry, with back pain as one of the most common causes of MSK-related sickness absence [17]. Back pain is often recurrent, and the majority of people who have activity-limiting back pain go on to have recurrent SA episodes [35]. A systematic review examining SA and return-to-work (RTW), found that approximately 20% of employees with back pain have some sickness absence episodes in the six to 12 month period following the back pain episode. RTW in this review was estimated at up to one month for 68% of individuals, 85% of individuals were back to work between 1–6 months and 93% were back to work at 6 months post the SA event [36]. Although RTW rates for back pain tend to be high, the recurrent nature of back pain increases the risk for substantial overall impact on work days lost through repeated periods of SA.

Neck problems are also a common MSK condition resulting in lost working days [17]. One cohort study investigating the relationship between physical and psychosocial features of the workplace and SA, found that work activities involving neck flexion and neck rotation, high job demands, low skill discretion, and low job security were significantly associated with SA due to neck pain [37].

Mental Health related absence

A number of systematic reviews highlight the importance of recognizing mental health issues in the workplace to assist in the reduction of associated SA [38 39]. However, there is limited evidence on how certain mental health conditions affect RTW times.

Depression is a leading cause of MH-related absence [14]. The extent and severity of depressive symptoms, comorbidity of anxiety, social and emotional support, education, and long symptom periods prior to diagnosis can affect the course of depression and RTW times [40-42].

For peer review only - http://bmjopen.bmj.com/site/about/guidelines.xhtml

For those experiencing anxiety that results in SA, previous anxiety episodes, older age, lower education levels, and long durations of untreated and undiagnosed symptoms, contribute to longer absences [42]. Detailed return-to-work figures following LTSA are scarce. Estimates suggest that as few as 13% of people who have experienced depression and anxiety are in employment, compared to 33% of people experiencing other chronic illnesses [43].

Socio-demographic and Occupational Factors and Sickness Absence

Several studies have investigated the associations between sickness absence and an employee's socio-demographic and socio-economic characteristics [19 44-48]. Evidence shows that sickness absence is multi-causal, and that in addition to providing support for an individual's specific characteristics and incapacitating condition, it is necessary to also consider an individual's work and workplace environment in order to effectively manage and improve return to work times [49 50].

Aim

Despite a great deal of research recognizing the prevalence of MSK and MH issues and their impact on SA rates [3 19-24]; there remains a lack of information on how certain types of musculoskeletal and mental health conditions may affect SA duration, which is vital to inform and improve current and new SA interventions, as well as support workplace modifications for SA prevention. This study aims to examine the impact of certain musculoskeletal and mental health conditions on sickness absence duration in healthcare workers, using a bespoke database [18 51] over a six year period, and to assess how health, socio-demographic factors, work characteristics and occupational factors affect SA duration.

For peer review only - http://bmjopen.bmj.com/site/about/guidelines.xhtml

Methods

Study population

The study population includes all participants in the EASY (Early Access to Support for You) SA management service of a Scottish health board [18 51]. EASY is a telephone-based service that provides early intervention (from Day 1 of absence) based on the biopsychosocial model [51 52] . Data on all staff who voluntarily engage with EASY (compliance rate was c.80%) are routinely entered into a bespoke database, including age, gender, job family, self-reported cause of absence, date of first day of absence, and return to work date [18 51]. Detailed descriptions of the EASY service can be found elsewhere [18 51]. We examined data on 66 490 unique absence events recorded in the EASY database between May 2008 and December 2014.

Defining and Recoding Variables

Sickness absence duration was calculated from the difference between the first date of absence and the RTW date. Cause of absence was grouped into eight categories: Respiratory, Musculoskeletal, Mental Health, Gastrointestinal, ENT, Cough/Cold/Flu, and All Other. Conditions assigned to the musculoskeletal category were grouped into nine subgroups according to anatomical site: Hip, Knee, Low Back, Lower Limb, Neck, Shoulder, Upper Limb, and All Other. Mental health conditions were allocated to eight subgroups according to diagnostic group: Bipolar Disorder, Anxiety, Depression, Schizophrenia, Panic attacks, Self-Harm, Stress, and All Other Psychiatric Disorders.

After missing data were eliminated (<0.05%), four main exclusion criteria were applied: (a) if there was no first day of absence (FDA) (N=196); (b) if the FDA was a Saturday or Sunday (N=4881), as there was no

For peer review only - http://bmjopen.bmj.com/site/about/guidelines.xhtml

SA service on those days and absences would not be recorded on FDA; (c) if the 'date opened' (i.e. the date the EASY service contacted the absentee) was before the first day of absence (N=828); (d) if the 'date opened' was equal to or after the RTW date (N=3 465). Due to overlaps among the missing data and exclusion criteria, a total of 13 286 absences were excluded, giving a total of 53 193 unique absence events; hence analysis was based on these 53 193 unique absence events.

Analytic Strategy

Descriptive Statistics

Descriptive statistics and crosstabs were produced for the entire EASY population, which included all causes of sickness absence (N=53 193). Descriptive statistics and crosstabs were then produced for two specific causes, MH (N=3 093) and MSK (N=6 969) conditions.

Mean absence duration was calculated for the entire EASY population in the study, the entire EASY population without MSK and MH (N=43 131), MSK only, MH only, MSK sub categories, and MH sub categories for each year from May 2008 to December 2015. The data were divided into seven years as follows: May 2008–April 2009 (Year 1); May 2009–April 2010 (Year 2); May 2010–April 2011 (Year 3); May 2011–April 2012 (Year 4); May 2012-April 2013 (Year 5); May 2013-April 2014 (Year 6); May 2014-December 2015 (Year 7). Absences commencing in Year 7 were not included as the year was incomplete and in numerous cases absence could still be ongoing; hence further analyses presented were based on 48 007 unique absence events.

For peer review only - http://bmjopen.bmj.com/site/about/guidelines.xhtml

Kaplan Meier Survival Analysis & Cox Proportional Hazards Model

Absence duration was analyzed using Kaplan Meier survival curves and Cox's proportional hazards models to determine the hazard ratios of absentees returning to work. We tested the assumption of proportional hazards using Schoenfeld residuals and where violated we corrected for this by adding the relevant time varying covariate (Table S1). The model takes into account each sickness absence event as well as individuals with multiple absence events by calculating cluster robust standard errors and the multivariate model controlled for several occupational and individual variables including: gender, age, job family, job type, cause of absence, day of absence, season of absence, and year of absence (Tables S2a-f).

All statistical analyses were conducted using STATA version 12.0 and R version 3.1.1.

Results

Our sample contains 11 694 individual people and 48,007 absences (May 08 – April 14). Descriptive statistics for our sample (May08-Dec14; N=48,007) are reported in **Table 1**. Almost 9 out of 10 (87.9%) absences were from female employees (N=42,187). Over the six year period investigated, the three most common causes of SA events are gastrointestinal (N=13,459), cold, cough, and flu (N=8,657), and musculoskeletal (N=6,530) problems. Almost half the absences (45.3%) were from the nursing/midwifery job category, and a further 20% in administrative services. Numbers in part-time (43.2%) and full-time employment (56.8%) were relatively similar.

Fewer of those with MSK-related absences (N=6,530) were in the two youngest age groups, than in the

For peer review only - http://bmjopen.bmj.com/site/about/guidelines.xhtml

total population, and the proportion of those with MSK absences who were in the Nursing/midwifery (N=3,428) job category (52.5%) was even higher than seen in the total population (45.3%). In the MH population, 91.8% are female, compared to 87.9% of the total population (Table 1).

Table 1. Descriptive statistics for absences in the total EASY population, and MSK and MH subgroups

		Total Sample (N=48,007)		Total MSK Absences (N=6,530)		Total MH Absences (N=2,921)	
		N	%	N	%	N	%
Age Group	16-29	6,885	14.34	588	9.00	253	8.66
	30-39	10,772	22.44	1,124	17.21	639	21.88
	40-49	15,257	31.78	2,291	35.08	1,102	37.73
	50-59	13,003	27.09	2,181	33.40	813	27.83
	60+	2,090	4.35	346	5.30	114	3.90
Gender	Male	5,820	12.12	963	14.75	241	8.25
	Female	42,187	87.88	5,567	85.25	2,680	91.75
Job Family	Administrative Services	9,597	19.99	1,003	15.36	550	18.83
	Allied Health Profession	4,914	10.24	502	7.69	208	7.12
	Healthcare Sciences	2,130	4.44	284	4.35	105	3.59
	Manager	150	0.31	13	0.20	5	0.17
	Medical & Dental	1,301	2.71	125	1.91	33	1.13
	Medical & Dental Support	876	1.82	83	1.27	35	1.20
	Nursing/Midwifery	21,734	45.27	3,428	52.50	1,614	55.26
	Other Therapeutic	2,100	4.37	193	2.96	74	2.53
	Personal and Social Care	443	0.92	56	0.86	18	0.62
	Support Services	4,762	9.92	843	12.91	279	9.55
Job Type	Part time	20,758	43.24	2,871	43.97	1,431	48.99

For peer review only - http://bmjopen.bmj.com/site/about/guidelines.xhtml

Full time	27,249	56.76	3,659	56.03	1,490	51.01

Mean duration (in days) of absence by cause of sickness

Gastrointestinal (GI) and cold, cough, and flu (CCF) problems account for the largest number of sickness absence *events*, 28% and 18%, respectively. However, Figure 1a shows that the *impact*, in number of days absent, these causes have on total number of working days lost is much less (11.8% for gastrointestinal and 6.3% for cold, cough and flu). The health conditions with the highest impact on total number of working days lost are musculoskeletal (24%) and mental health (20%) conditions. Mean absence duration ranged from 5.6 days for cold, cough and flu to 53.3 days for MH-related absences. MSK absences had an overall mean duration of 28.9 days.

Insert Figure 1.

The three most common types of MSK problems in this population are low back pain (33.6% of all SA events), lower limb (9%), and upper limb problems (9%). Figure 1b shows that, within the subgroup with absences due to MSK, low back pain had the highest percent impact on total number of working days lost (26.8%), followed by upper limb problems (12.3%), and lower limb problems (9.1%). Mean absence duration within the MSK-related absences ranged from 17 days due to neck problems to 40 days for upper limb. All other MSK conditions did not differ greatly in duration, and ranged from 23 to 34 days.

The three most common types of MH problems amongst SA episodes in the EASY population are stress (64.8%), anxiety (15.9%), and depression (13.7%). The job categories with the highest cases of MH-

For peer review only

related absences are nursing/midwifery (55.3%), and administrative services (18.8%). Figure 1c demonstrates that stress accounts for the largest percent of working days lost (62.7%), followed by depression (18.9%) and anxiety (14.6%). There was no significant change year on year in mean duration of MH-related absences (Figures S1a-c), with depression resulting in the longest absences in all years (overall mean duration of 72.1 days). Anxiety and stress related absences had a mean duration of 48.1 and 50.7 days, respectively.

Mean absence duration in years two, three, four, five, and six of the EASY service was compared to year one using linear regression for all causes of SA to examine potential significant changes over time, and then for each of the nine conditions (Figure S1a in the Supplementary Material). Compared to year one, mean absence duration for MSK cases in Year 2, Year 3 and Year 4 were significantly lower (Figure S1a in the Supplementary Material).; mean absence duration for gastrointestinal cases in years five and six was also significantly lower; and mean absence duration for cold, cough, and flu cases in years two, five and six was significantly lower (Figure S1a in the Supplementary Material).

For peer review only

<u>Sickness absence duration & return to work</u>

Figure 2 shows the Kaplan Meier RTW curves for all sickness absence events minus MSK and MH related absences (Figure 2a), for the MSK-related absences (Figure 2b, and Figure S2 in Supplementary Material) and for the MH-related absences (Figure 2c, and Figure S3 in Supplementary Material). RTW for staff absent because of MH problems was much longer than all other causes of absences (Figure 2a). For example, 50% for staff absent from work due to a MH problem had returned to work by 35 days (median), whereas 50% of those with an absence due to an MSK condition or all other conditions had returned within 10 and 5 days (respectively) of their FDA. As shown in Figure 2b, there are significant

For peer review only - http://bmjopen.bmj.com/site/about/guidelines.xhtml

differences in RTW duration by sub-conditions within absences due to a MSK condition. Upper limb conditions result in the longest absences (50% of staff RTW by 25 days) whereas lower back and neck problems, result in the shortest absences (50% of staff RTW by 7 days for both conditions). For the other musculoskeletal conditions (knee, lower limb, shoulder and other), 50% of the population RTW (P_{50}) between 10 and 14 days. Mental health related absences are much longer (**Figure 2c**). Depression is the leading cause of longer SA events, with 50% of staff RTW by 53 days, followed by stress (50% of staff RTW by 34 days) and anxiety and Other mental health conditions (50% of staff RTW by 29 days).

Table 2: Multivariate cox regression RTW hazard ratios for all SA episodes (minus MSK and MH), MSK and MH conditions with Time Varying Coefficients.

	All Conditions (minus MSK & MH)			Musculoskeletal Conditions			Mental Health Conditions		
	**HR**	**95% CI**	**P**	**HR**	**95% CI**	**P**	**HR**	**95% CI**	**P**
**Population**									
All EASY except MSK & MH	1			-	-	-	-	-	-
MSK	0.48	(0.47, 0.50)	0.000	-	-	-	-	-	-
MH	0.23	(0.22, 0.24)	0.000	-	-	-	-	-	-
**MSK condition**									
Lower back	-	-	-	1			-	-	-
Knee	-	-	-	0.83	(0.74, 0.94)	0.003	-	-	-
Lower limb	-	-	-	0.79	(0.72, 0.86)	0.000	-	-	-
Neck	-	-	-	1.17	(1.05, 1.30)	0.006	-	-	-
Shoulder	-	-	-	0.77	(0.68, 0.87)	0.000	-	-	-
Upper limb	-	-	-	0.60	(0.55, 0.65)	0.000	-	-	-
Other	-	-	-	0.72	(0.67, 0.78)	0.000	-	-	-
**MH condition**									
Depression	-	-	-	-	-	-	1		
Anxiety	-	-	-	-	-	-	1.64	(1.39, 1.93)	0.000
Stress	-	-	-	-	-	-	1.65	(1.39, 1.95)	0.000
Other	-	-	-	-	-	-	1.78	(1.38, 2.30)	0.000
**Gender**									
Male	1			1			1		

For peer review only - http://bmjopen.bmj.com/site/about/guidelines.xhtml

Female	0.89	(0.87, 0.94)	0.000	0.74	(0.68, 0.81)	0.000	1.07	(0.93, 1.23)	0.374
**Age**	0.99	(0.99, 0.99)	0.000	0.99	(0.99, 0.99)	0.000	0.99	(0.99, 1.00)	0.000
**Job category**									
Nursing/Midwifery	1			1			1		
Administrative services	1.21	(1.17, 1.25)	0.000	1.23	(1.12, 1.35)	0.000	0.98	(0.88, 1.08)	0.647
Allied Health Profession	1.33	(1.28, 1.39)	0.000	1.19	(1.07, 1.33)	0.001	1.15	(1.00, 1.33)	0.057
Healthcare Sciences	1.22	(1.15, 1.29)	0.000	1.31	(1.13, 1.53)	0.000	1.10	(0.88, 1.38)	0.419
Manager	1.36	(1.11, 1.66)	0.003	1.80	(1.05, 3.07)	0.033	1.46	(0.79, 2.69)	0.232
Medical & Dental	1.45	(1.34, 1.58)	0.000	1.24	(0.98, 1.57)	0.073	1.51	(0.94, 2.43)	0.085
Medical and Dental Support	1.16	(1.06, 1.27)	0.002	1.40	(1.06, 1.84)	0.016	0.91	(0.61, 1.35)	0.631
Other Therapeutic	1.52	(1.43, 1.63)	0.000	1.44	(1.19, 1.75)	0.000	1.24	(0.91, 1.68)	0.173
Personal and Social Care	1.14	(1.01, 1.28)	0.033	1.69	(1.26, 2.27)	0.000	0.78	(0.49, 1.22)	0.276
Support Services	1.01	(0.97, 1.05)	0.642	1.07	(0.99, 1.16)	0.103	1.21	(1.05, 1.40)	0.009
**Job type**									
Part time	1			1			1		
Full time	1.13	(1.10, 1.16)	0.000	1.04	(0.99, 1.11)	0.135	1.09	(1.00, 1.18)	0.044
**Year**									
May 08-Apr 09	1			1			1		
May 09-Apr 10	0.98	(0.95, 1.02)	0.389	1.14	(1.03, 1.26)	0.014	0.99	(0.85, 1.15)	0.884
May 10-Apr 11	0.95	(0.91, 0.99)	0.008	1.14	(1.03, 1.26)	0.013	0.99	(0.84, 1.16)	0.857
May 11-Apr 12	0.93	(0.90, 0.97)	0.001	1.11	(1.01, 1.23)	0.034	0.91	(0.79, 1.06)	0.249
May 12-Apr 13	0.91	(0.88, 0.95)	0.000	0.97	(0.88, 1.08)	0.627	0.89	(0.76, 1.04)	0.145
May 13-Apr 14	0.95	(0.92, 0.99)	0.018	1.07	(0.97, 1.19)	0.159	1.03	(0.88, 1.21)	0.687
TVC									
Population	1.01	(1.01, 1.01)	0.000	1.00	(1.00, 1.00)	0.000	1.00	(1.00, 1.0)	0.03
Age	1.00	(1.00, 1.00)	0.787						
Sex	1.00	(1.00, 1.00)	0.012	1.00	(1.00, 1.01)	0.000			
Job Category	1.00	(1.00, 1.00)	0.000	1.00	(1.00, 1.00)	0.019			
Job Type	1.00	(1.00, 1.00)	0.003						
Year	1.00	(1.00, 1.00)	0.268						

Insert Figure 2.

For peer review only - http://bmjopen.bmj.com/site/about/guidelines.xhtml

Multivariate Analysis for all EASY absences (minus MSK & MH), MSK and MH

Table 2 shows the results of the multivariate cox regression analysis to investigate the differences in the risk of return to work (RTW) between the three main absence groups, gender, job title and year of absence. In the analysis for the entire population, the proportional hazards assumption was not met for the population, sex, age job category, job type, day or year variables (See Table S1 in Supplementary Material). For the musculoskeletal injuries, the population, sex and job category variables violated the proportional hazards assumption, while for the mental health analysis the population and day variables violated it and therefore the analyses were re-run including these variables as time varying coefficients (Table 2 and Tables S2a-f).

In terms of absence cause, in comparison to all of EASY SA episodes (minus episodes due to MSK & MH), the risk of RTW was 52% (HR: 0.48; 95% CI .47-.50, P<0.0001) and 77% (HR: 0.23;95% CI .22-.24, P<0.0001) longer for absences due to MSK and MH conditions, respectively. No real differences were seen after adjusting for gender, age, job family, job type, cause of absence, day of absence, season of absence, and year of absence.

For MSK-related absences, staff absent due to neck problems were at risk of being off 17% longer (HR: 1.17; 95% CI 1.05-1.30, P=0.006) compared to those absent because of lower back problems, whereas employees who were absent because of all other MSK conditions (HR: 0.72; CI: 0.67, 0.78) had quicker RTW.

The risk of returning to work after being absent due to anxiety, stress and all other mental health related absences was significantly higher, when compared to depression, and were 64% (HR: 1.64; 95% CI 1.39-1.93), 65% (HR: 1.65; 95% CI 1.39- 1.95) and 78% (HR: 1.78; 95% CI 1.38-2.30) of longer duration,

For peer review only - http://bmjopen.bmj.com/site/about/guidelines.xhtml

respectively (all p<0.001).

The analysis by gender demonstrated that for all absences (minus MSK & MH) and for MSK absences women exhibited longer RTW times than men (Table 2). No significant gender difference in RTW was observed for mental health conditions. From the data, it is not possible to ascertain, whether full-time or part-time working has any association with RTW, as hours and days of employment were not included in the database.

In terms of job category, for all conditions (minus MSK & MH) nurses had the longest SA duration prior to RTW followed by staff in 'support services' (Table 2). Employees in 'other therapeutic services' and 'medical and dental' staff demonstrated 52% (HR: 1.52; 95%CI 1.43-1.63) and 45% (HR: 1.45; 95%CI 1.34-1.58), respectively, shorter times to RTW than staff in 'nursing/midwifery'. For MSK related absence, the only differences to the reference category 'nursing/midwifery' was observed for 'support services' (HR: 1.07; CIs: 0.99, 1.16; p=0.103) and 'medical & dental' (HR: 1.24; 95%Ci 0.98-1.57; p=0.073) but these fell just short of conventional levels of significance. For mental health related absences, only 'support services' (HR: 1.21; 95%CI 1.05=1.40; p=0.009) staff had significantly shorter time to RTW compared to 'Nursing/Midwifery' staff.

The analysis by year of absence using Year 1 as the reference category, showed that staff took significantly longer to return to work in years 3-6 for all conditions (minus MSK & MH). Amongst MSK absences, in years 2-4 staff time to RTW was significantly shorter than for year 1. There were no significant differences in time to RTW by year for staff absences due to a mental health condition. Additionally, when examining time to RTW by day of absence start, absences starting on a Tuesday and Friday took significantly longer to RTW (Tables S2a-f). No significant differences in time to RTW were

For peer review only

For peer review only - http://bmjopen.bmj.com/site/about/guidelines.xhtml

observed for season in which the absence occurred ($p>0.05$) (Tables S2a-f).

Discussion

Summary of findings

We found significant differences in sickness absence duration by presenting condition in a population of healthcare workers. Mental health conditions, and depression specifically, accounted for the most working days' absence. We also observed significant variations in duration for different musculoskeletal conditions. Upper limb disorders resulted in the longest sickness absence durations among these healthcare workers, with 50% of staff returning to work by day 25. Lower back and neck problems resulted in the shortest MSK-related absences (50% RTW by 7 days), whereas knee, lower limb, shoulder and other MSK conditions resulted in SA durations that were comparable to each other (50% RTW between 10-14 days).

Employees within the nursing and midwifery job category accounted for almost half (45.3%) of all sickness absence events recorded in the six year period, and over half of the MSK (52.5%) and MH-related (55.3%) absence events. Nurses and midwives also had longer times to RTW than every other job category. In terms of gender, no significant differences were detected in RTW between men and women who were absent from work because of mental health conditions, but for all other absence events women took longer to return to work than men.

Research in context to previous studies

For peer review only - http://bmjopen.bmj.com/site/about/guidelines.xhtml

The results of our study are in agreement with previous published work on the impact of musculoskeletal and mental health conditions on sickness absence and return to work [2 14 53-55]. A Danish cohort study aiming to identify prognostic factors associated with neck-shoulder pain resulting in long term sickness absence, found pain intensity and job characteristics, such as heavy physical workload, were significantly associated with longer absence duration [56]. Armijo-Olivo et al. (2016) also demonstrated that occupation and health condition were significant factors, among others, in the rehabilitation process of people affected by MSK conditions [57]. Similarly for mental health conditions, previous research suggests that sickness absence associated with psychological ill-health tends to be higher among NHS healthcare workers than for other employment sectors in the UK [58]. This may be due to the pressured nature of the work, constant organizational changes, and the large workload [59], supporting the notion that organizational factors may contribute to the level of psychological ill health experienced by staff. These findings are also reflected in our study, where significant variations in absence duration are observed not only by certain MSK and MH conditions, but also by job categories - with nursing and midwifery staff experiencing the majority of and longest SA events. One study looking at job family and sickness absence in the healthcare sector, reported that doctors had nine times lower rates of short-term sickness absence and four times lower rates of long-term sickness absence, while nurses had three times lower rates of short-term sickness absence when compared to other healthcare sector job families [44].

<u>Strengths of the study</u>

The latest Health and Safety Executive (HSE) report lists healthcare staff as one of the categories with

For peer review only - http://bmjopen.bmj.com/site/about/guidelines.xhtml

the highest sickness absence rates, and mental health and MSK as leading causes [14]. This study is therefore particularly important as we are able to investigate in detail the durations of SA events by particular MSK and MH conditions in this population. The unique and rich EASY database [51] enables analysis of routine data collected in a systemic way across all job categories within the healthcare sector. While several studies have reviewed sickness absence in healthcare settings, these have been limited in several ways. For instance, they examine a narrow range of healthcare workers, mainly doctors and nurses; or examine only broad categories of absence [39]. Thus, the size of the EASY database base, giving six full years of sickness absence and return to work data, and the range of variables collected - including demographic, job, start and end dates, self-reported conditions - are considerable strengths.

<u>Study limitations</u>

While the availability of such rich data on routine sickness absence is a major strength of this study, there are also some limitations to the data. This SA management service is only available Monday to Friday and therefore not all absences may have been recorded. To avoid any confounding we removed absences starting on a weekend. The cause of absence is self-reported by the employee when they call the service and not based on a clinical diagnosis, and co-morbidities - which may also impact on absence duration - are not collected [53].

Sickness absence is multi-causal and it is necessary to consider an individual's work and workplace environment. However, the lack of granularity in SA data recorded is often the barrier into investigating the risk factors and causes impacting on SA duration. Linking bespoke databases, such as the one in this study, to personnel data or having uniformity in the variables collected across health boards would allow

For peer review only - http://bmjopen.bmj.com/site/about/guidelines.xhtml

investigation of the impact of operational (e.g. shift work) and organisational (region, structures) risk factors on sickness absence rates and duration.

As this health board has a unique SA management service, the results may not be representative of all healthcare workers. However, a national standard requires all health boards in Scotland to work towards a 4% or less sickness absence rate [60]. While this health board had a higher SA rate in early 2008, by the end of 2008 the SA rate had fallen to similar levels to the other health boards [18]. The latest data show that this health board's SA rate of 5.20% is in line with the Scottish NHS average of 5.16% [60].

Implications for policy and practice

Due to the financial and morale repercussions sickness absence amongst healthcare employees has not only on healthcare staff themselves, but also their patients and employers [31], it is an important focus of attention for healthcare management, and for public health policies and practice reform. Recent systematic reviews have evaluated the effectiveness of different types of SA interventions and have found that multidisciplinary interventions involving collaboration between employees, health practitioners and employers working to implement tailored modifications for the absentee were consistently more effective than generic non-tailored interventions targeted at all employees [13 61].

Conclusions

The results of this study further establish the need for occupational health, organizational and management interventions to address recognized individual and workplace stressors that can impact on sickness absence duration. Our results suggest that employees with upper limb problems and depressive

For peer review only

symptoms in particular, could benefit from more tailored interventions to assist them in the return to work process following a SA event. A great burden of work loss due to both musculoskeletal and mental health conditions was observed for nurses and midwives.

This research is important in terms of improving the health and wellbeing of NHS staff but may also improve the quality of patient care, and subsequently public health. SA has far-ranging economic consequences for both employers and employees, as it simultaneously impacts on NHS resources/service delivery and on people's earnings if the SA is prolonged. These findings give a deeper insight into the link between health, organizational, operational and sociodemographic factors influencing sickness absence. Understanding these relationships allows health providers to be better placed to plan the allocation of resources, build better models of sickness absence management and inform the development of tailored sickness absence interventions for NHS staff.

Acknowledgements

The authors would like to acknowledge Mark Kennedy, Keith Murray, Moyra Anderson, and Raymond Hamill for their support.

Author Contributions

ED developed the study and was the main author of the manuscript. AB and SS conducted the statistical analysis and contributed to the manuscript writing. DFM advised on the statistical analysis. DFM, JB, KH, SVP and EBM all reviewed and contributed to the manuscript.

For peer review only - http://bmjopen.bmj.com/site/about/guidelines.xhtml

Ethical Considerations

NHS Lanarkshire Research and Development (R&D) Management Approval (ref number: L11071).

Funding

This work was supported by the Medical Research Council partnership grant MC/PC/13027 to ED and funding from the UK MRC and Chief Scientist Office (MC_UU_12017/12; SPHSU12) to AB and KH.

Competing Interests

None

Data Sharing Statement

No additional data available

References

1. Henderson M, Glozier N, Elliott KH. Long term sickness absence - Is caused by common conditions and needs managing. *BMJ* 2005;330(7495):802-03. doi: 10.1136/bmj.330.7495.802
2. Ritchie KA, Macdonald EB, Gilmour WH, et al. Analysis of sickness absence among employees of four NHS trusts. *Occup Environ Med* 1999;56(10):702-08.
3. Wise J. Audit finds large variations in NHS staff sick days. *BMJ* 2011;342 doi: 10.1136/bmj.d953
4. Hussey L, Turner S, Thorley K, et al. Work-related sickness absence as reported by UK general practitioners. *Occupational Medicine* 2012;62(2):105-11. doi: 10.1093/occmed/kqr205

For peer review only - http://bmjopen.bmj.com/site/about/guidelines.xhtml

5. Pekkala J, Blomgren J, Pietiläinen O, et al. Occupational class differences in long sickness absence: a register-based study of 2.1 million Finnish women and men in 1996–2013. *BMJ Open* 2017;7(7) doi: 10.1136/bmjopen-2016-014325
6. Black C. Health at Work - an independent review of sickness absence. In: The Stationary Office, ed., 2011.
7. Vahtera J, Kivimaeki M. Reducing sickness absence in occupational settings. *Occup Environ Med* 2008;65(4):219-20. doi: 10.1136/oem.2007.036061
8. Fit for Work. http://www.fitforwork.org/. 2015
9. Health Works. *A review of the Scottish Government's Healthy Working Lives Strategy,* The Scottish Government, Edinburgh, Available from: http://www.scotland.gov.uk/Publications/2009/12/11095000/0. In: Government TS, ed. Edinburgh, 2009.
10. Lammerts L, Schaafsma FG, Eikelenboom M, et al. Longitudinal Associations Between Biopsychosocial Factors and Sustainable Return to Work of Sick-Listed Workers with a Depressive or Anxiety Disorder. *Journal of occupational rehabilitation* 2016;26(1):70-9. doi: 10.1007/s10926-015-9588-z [published Online First: 2015/06/22]
11. van Vilsteren M, van Oostrom SH, de Vet HCW, et al. Workplace interventions to prevent work disability in workers on sick leave. *Cochrane Database of Systematic Reviews* 2015;Issue 10. Art. No.: CD006955.
12. Burton A, Kendall N, McCluskey S, et al. Telephonic support to facilitate return to work: what works, how, and when? In: Department for Work and Pensions, ed. London, 2013.
13. van Amelsvoort LG, Jansen NW, Kant I. Addressing long-term sickness absence: moving beyond disease, illness and work-related factors for effective prevention. *Scandinavian journal of work, environment & health* 2017(1):1-4. doi: 10.5271/sjweh.3605
14. HSE. Health and Safety Statistics, Annual report for Great Britain, 2014/2015 (Avaialbale from: http://www.hse.gov.uk/statistics/overall/hssh1415.pdf). In: Executive HaS, ed., 2015.
15. Boorman S. NHS Health and Well-being - Final Report. Leeds: Department of Health; 2009. [http://webarchive.nationalarchives.gov.uk/20130107105354/http://www.dh.gov.uk/prod_consum_dh/groups/dh_digitalassets/documents/digitalasset/dh_108907.pdf]. 2009
16. Suff P. Absence in the NHS. *Occup Health at Work* 2013;10(3):19-23.
17. ONS. Sickness absence in the labour market: february 2014. Available from: http://www.ons.gov.uk/employmentandlabourmarket/peopleinwork/labourproductivity/articles/sicknessabsenceinthelabourmarket/2014-02-25 2014 [
18. Brown J, Mackay D, Demou E, et al. The EASY (Early Access to Support for You) sickness absence service: a four-year evaluation of the impact on absenteeism. *Scand J Work Environ Health,* 2015;41(2):204-15. doi: 10.5271/sjweh.3480 [published Online First: 2015/01/28]
19. Gorman E, Yu S, Alamgir H. When healthcare workers get sick: exploring sickness absenteeism in British Columbia, Canada. *Work (Reading, Mass)* 2010;35(2):117-23. doi: 10.3233/wor-2010-0963 [published Online First: 2010/02/19]
20. Andersen LL, Clausen T, Burr H, et al. Threshold of Musculoskeletal Pain Intensity for Increased Risk of Long-Term Sickness Absence among Female Healthcare Workers in Eldercare. *Plos One* 2012;7(7) doi: 10.1371/journal.pone.0041287

For peer review only - http://bmjopen.bmj.com/site/about/guidelines.xhtml

21. Franche RL, Murray EJ, Ostry A, et al. Work disability prevention in rural areas: a focus on healthcare workers. *Rural and Remote Health* 2010;10(4)
22. Murphy IJ. Self-reported and employer-recorded sickness absence in doctors. *Occupational medicine (Oxford, England)* 2014;64(6):417-20. doi: 10.1093/occmed/kqu098
23. Roelen CAM, Mageroy N, van Rhenen W, et al. Low job satisfaction does not identify nurses at risk of future sickness absence: Results from a Norwegian cohort study. *International Journal of Nursing Studies* 2013;50(3):366-73. doi: 10.1016/j.ijnurstu.2012.09.012
24. van der Meer EWC, Boot CRL, van der Gulden JWJ, et al. Hand eczema among healthcare professionals in the Netherlands: prevalence, absenteeism, and presenteeism. *Contact Dermatitis* 2013;69(3):164-71. doi: 10.1111/cod.12099
25. Lalloo D, Demou E, Macdonald EB. Trends in NHS doctor and dentist referrals to occupational health. *Occupational medicine (Oxford, England)* 2016;66(4):316-9. doi: 10.1093/occmed/kqw016 [published Online First: 2016/03/02]
26. Alexis O. The management of sickness absence in the NHS. *British journal of nursing (Mark Allen Publishing)* 2011;20(22):1437-40, 42. doi: 10.12968/bjon.2011.20.22.1437 [published Online First: 2012/01/14]
27. Johnson CJ, Croghan E, Crawford J. The problem and management of sickness absence in the NHS: considerations for nurse managers. *Journal of nursing management* 2003;11(5):336-42. doi: 10.1046/j.1365-2834.2003.00404.x
28. McEwan IM. Absenteeism and sickness Absence. *Postgraduate Medical Journal* 1991;67(794):1067-71.
29. HSE. Health and safety at work. Summary statistics for Great Britain 2016. Available from: http://www.hse.gov.uk/statistics/overall/hssh1516.pdf?pdf=hssh1516. In: Executive HaS, ed., 2016.
30. HSE. Health and safety in the health and social care sector in Great Britain, 2014/15. Available from: http://www.hse.gov.uk/statistics/industry/healthservices/health.pdf?pdf=health. In: Executive HaS, ed., 2015.
31. Professor Sir Bruce Keogh KBE. Section 4.3. Workforce; "Review into the quality of care and treatment provided by 14 hospital trusts in England: overview report".

http://www.nhs.uk/nhsengland/bruce-keogh-review/documents/outcomes/keogh-review-finalreport.Pdf, 2013.
32. NICE. Depression: Treatment and Management of Depression in Adults. Clinical Guideline 90. London: Available at www.nice.org.uk. 2009
33. NICE. Anxiety: Management of Anxiety (Panic Disorder, With and Without Agoraphobia, and Generalised Anxiety Disorder) in Adults in Primary, Secondary and Community Care. Clinical Guideline 113. London: . Available at www.nice.org.uk. 2011
34. Wiberg M, Friberg E, Palmer E, et al. Sickness absence and subsequent disposable income: A population-based cohort study. *Scand J Public Health* 2015;43(4):432-40. doi: 10.1177/1403494815575341 [published Online First: 2015/03/31]
35. Hoy D, March L, Brooks P, et al. The global burden of low back pain: estimates from the Global Burden of Disease 2010 study. *Annals of the rheumatic diseases* 2014;73(6):968-74. doi: 10.1136/annrheumdis-2013-204428 [published Online First: 2014/03/26]

For peer review only - http://bmjopen.bmj.com/site/about/guidelines.xhtml

36. Wynne-Jones G, Cowen J, Jordan JL, et al. Absence from work and return to work in people with back pain: a systematic review and meta-analysis. *Occupational and Environmental Medicine* 2013 doi: 10.1136/oemed-2013-101571
37. Ariens GA, Bongers PM, Hoogendoorn WE, et al. High physical and psychosocial load at work and sickness absence due to neck pain. *Scandinavian journal of work, environment & health* 2002;28(4):222-31. [published Online First: 2002/08/30]
38. Dewa CS, Loong D, Bonato S. Work outcomes of sickness absence related to mental disorders: a systematic literature review. *BMJ open* 2014;4(7) doi: 10.1136/bmjopen-2014-005533
39. Michie S, Williams S. Reducing work related psychological ill health and sickness absence: a systematic literature review. *Occup Environ Med* 2003;60(1):3-9. [published Online First: 2002/12/25]
40. Judd LL, Schettler PJ, Solomon DA, et al. Psychosocial disability and work role function compared across the long-term course of bipolar I, bipolar II and unipolar major depressive disorders. *Journal of Affective Disorders* 2008;108(1-2):49-58. doi: 10.1016/j.jad.2007.06.014
41. Soderman E, Lisspers J, Sundin O. Depression as a predictor of return to work in patients with coronary artery disease. *Social science & medicine (1982)* 2003;56(1):193-202. [published Online First: 2002/11/19]
42. van den Brink RHS, Ormel J, Tiemens BG, et al. Predictability of the one-year course of depression and generalized anxiety in primary care. *General Hospital Psychiatry* 2002;24(3):156-63. doi: 10.1016/s0163-8343(02)00183-4
43. Munir F, Yarker J, Haslam C, et al. Returning to Work - The Role of Depression. Available from: http://www.mentalhealth.org.uk/publication-download/returning-work-role-depression. In: Foundation TMH, ed. Loughborough,, 2009.
44. Lim A, Chongsuvivatwong V, Geater A, et al. Influence of work type on sickness absence among personnel in a teaching hospital. *Journal of Occupational Health* 2002;44(4):254-63. doi: 10.1539/joh.44.254
45. Sandanger I, Nygard JF, Brage S, et al. Relation between health problems and sickness absence: gender and age differences--a comparison of low-back pain, psychiatric disorders, and injuries. *Scand J Public Health* 2000;28(4):244-52. [published Online First: 2001/03/03]
46. Krane L, Johnsen R, Fleten N, et al. Sickness absence patterns and trends in the health care sector: 5-year monitoring of female municipal employees in the health and care sectors in Norway and Denmark. *Human Resources for Health* 2014;12 doi: 10.1186/1478-4491-12-37
47. Kristensen TR, Jensen SM, Kreiner S, et al. Socioeconomic status and duration and pattern of sickness absence. A 1-year follow-up study of 2331 hospital employees. *BMC public health* 2010;10 doi: 10.1186/1471-2458-10-643
48. d'Errico A, Costa G. Socio-demographic and work-related risk factors for medium- and long-term sickness absence among Italian workers. *European journal of public health* 2012;22(5):683-8. doi: 10.1093/eurpub/ckr140 [published Online First: 2011/12/14]
49. MacEachen E, Clarke J, Franche R-L, et al. Systematic review of the qualitative literature on return to work after injury. *Scand J Work Environ Health* 2006;32(4):257-69.
50. Macdonald EB, Asanati K. Absence from work. *Contemporary Social Science* 2016;11(1):30-39. doi: 10.1080/21582041.2016.1246749

For peer review only - http://bmjopen.bmj.com/site/about/guidelines.xhtml

51. Demou E, Brown J, Sanati K, et al. A novel approach to early sickness absence management: The EASY (Early Access to Support for You) way. *Work (Reading, Mass)* 2015;53(3):597-608. doi: 10.3233/wor-152137 [published Online First: 2015/09/28]
52. Engel GL. Need for a new medical model - Challenge for biomedicine. *Science* 1977;196(4286):129-36. doi: 10.1126/science.847460
53. Ervasti J, Mattila-Holappa P, Joensuu M, et al. Predictors of Depression and Musculoskeletal Disorder Related Work Disability Among Young, Middle-Aged, and Aging Employees. *Journal of Occupational and Environmental Medicine* 2017;59(1):114-19. doi: 10.1097/jom.0000000000000921
54. Lotters F, Burdorf A. Prognostic factors for duration of sickness absence due to musculoskeletal disorders. *The Clinical journal of pain* 2006;22(2):212-21. [published Online First: 2006/01/24]
55. Nieuwenhuijsen K, Verbeek JH, de Boer A, et al. Predicting the duration of sickness absence for patients with common mental disorders in occupational health care. *Scandinavian Journal of Work Environment & Health* 2006;32(1):67-74.
56. Holtermann A, Hansen JV, Burr H, et al. Prognostic factors for long-term sickness absence among employees with neck-shoulder and low-back pain. *Scandinavian journal of work, environment & health* 2010;36(1):34-41. [published Online First: 2009/12/08]
57. Armijo-Olivo S, Woodhouse LJ, Steenstra IA, et al. Predictive value of the DASH tool for predicting return to work of injured workers with musculoskeletal disorders of the upper extremity. *Occupational and Environmental Medicine* 2016 doi: 10.1136/oemed-2016-103791
58. Williams S, Michie S, Pattani S. Improving the health of the NHS workforce. London: Nuffield Trust; http://discovery.ucl.ac.uk/16094/. 1998
59. Cox T. The nature and measurement of stress. *Ergonomics* 1985;28(8):1155-63. doi: 10.1080/00140138508963238 [published Online First: 1985/08/01]
60. ISD NHS. NHSScotland Workforce Information. Quarterly update of Staff in Post, Vacancies and Turnover at 31 March 2016. Available from: https://www.isdscotland.org/Health-Topics/Workforce/Publications/2016-06-07/2016-06-07-Workforce-Report.pdf. In: Scotland NNS, ed., 2016.
61. Durand MJ, Corbiere M, Coutu MF, et al. A review of best work-absence management and return-to-work practices for workers with musculoskeletal or common mental disorders. *Work (Reading, Mass)* 2014;48(4):579-89. doi: 10.3233/wor-141914 [published Online First: 2014/07/06]

For peer review only - http://bmjopen.bmj.com/site/about/guidelines.xhtml

Figure Captions:

Figure 1. Cause of sickness absence (% of total number of events & % Impact) for (a) all conditions, (b) MSK conditions**, and (c) mental health conditions***

*Impact is estimated as percent impact and calculated by number of events times the average condition-specific absence duration divided by the sum of impact for all causes times 100

**Number of absences due to 'Hip' were to small and grouped into 'Other' category

*** Number of absences due to 'Bipolar Disorder', 'Schizophrenia', 'Panic Attacks' and 'Self harm' were to small and grouped into 'Other' category

Figure 2. a. Return to Work curves for (top) all absences with 95%CI; (bottom-left) b. MSK-related absences by MSK condition; and (bottom-right) c. MH-related absences by MH condition (for Survival curves with 95%CIs for MSK and MH conditions see Supplementary Material Figures S2 and S3

Figure S1a. Mean duration (in days) of absence by cause of sickness

Figure S1b. Mean duration (in days) of absence by MSK cause of sickness

Figure S1c. Mean duration (in days) of absence by MH cause of sickness

Figure S2. Return to Work curves for all MSK-related absences by MSK condition with 95%CI

Figure S3. Return to Work curves for all MH-related absences by MH condition with 95%CI

For peer review only

For peer review only - http://bmjopen.bmj.com/site/about/guidelines.xhtml

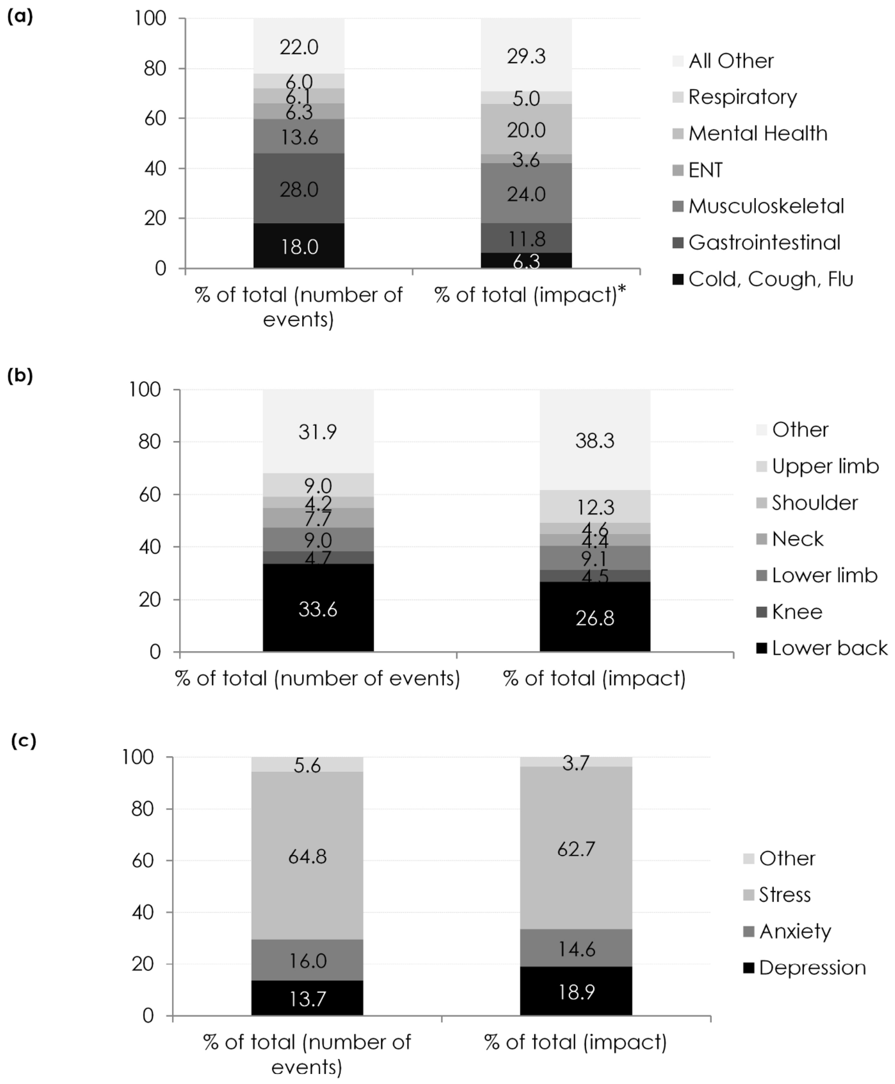

Figure 1. Cause of sickness absence (% of total number of events & % Impact) for (a) all conditions, (b) MSK conditions**, and (c) mental health conditions***
*Impact is estimated as percent impact and calculated by number of events times the average condition-specific absence duration divided by the sum of impact for all causes times 100
**Number of absences due to 'Hip' were to small and grouped into 'Other' category
*** Number of absences due to 'Bipolar Disorder', 'Schizophrenia', 'Panic Attacks' and 'Self harm' were to small and grouped into 'Other' category

225x300mm (300 x 300 DPI)

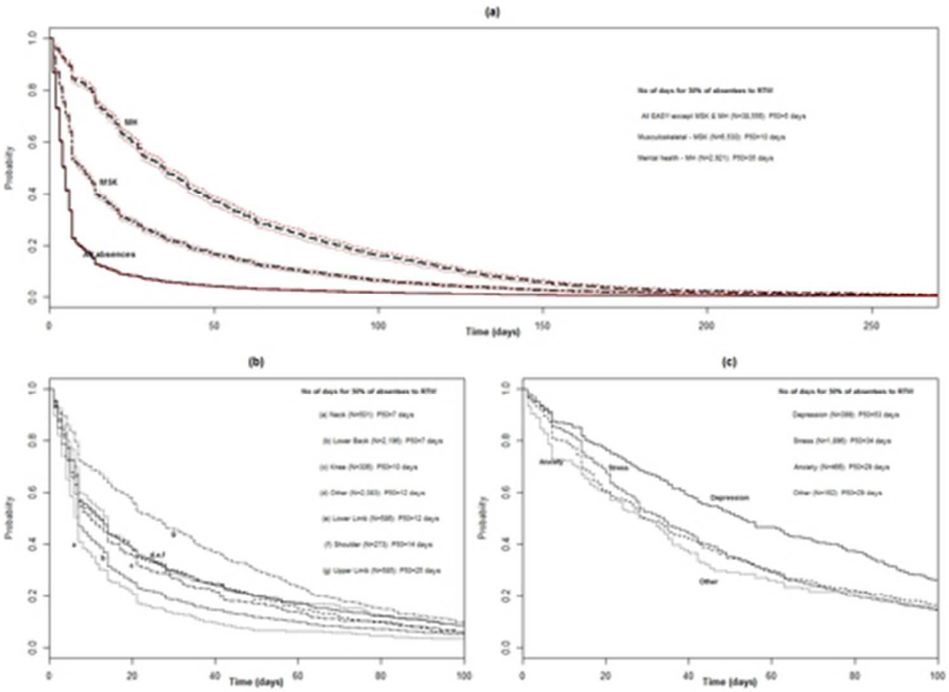

Figure 2. a. Return to Work curves for (top) all absences with 95%CI; (bottom-left) b. MSK-related absences by MSK condition; and (bottom-right) c. MH-related absences by MH condition (for Survival curves with 95%CIs for MSK and MH conditions see Supplementary Material Figures S2 and S3.

40x29mm (300 x 300 DPI)

Evaluating Sickness Absence Duration by Musculoskeletal and Mental Health Issues. A retrospective cohort study of Scottish Healthcare Workers

[Supplementary material]

Evangelia Demou PhD[1], Shanley Smith[2], Abita Bhaskar[1], Daniel F Mackay[2], Judith Brown PhD[2], Kate Hunt[1], Sergio Vargas-Prada MD[3,4], Ewan B Macdonald MD[2]

1. MRC/CSO Social and Public Health Sciences Unit, University of Glasgow, Glasgow, G2 3QB, United Kingdom

2. Healthy Working Lives Group, University of Glasgow, Glasgow, G12 8RZ, United Kingdom

3. Center for Research in Occupational Health (CiSAL), Universitat Pompeu Fabra, Barcelona, Spain

4. Unidad Central de Contingencias Comunes (U3C), ASEPEYO, Barcelona, Spain.

Running title Sickness Absence Duration in Healthcare Workers

Corresponding Author

Dr Evangelia Demou

Research Fellow

MRC/CSO Social and Public Health Sciences Unit

University of Glasgow

200 Renfield Street, Glasgow, G2 3QB, UK

Tel: 0141 353 7580

For peer review only

For peer review only - http://bmjopen.bmj.com/site/about/guidelines.xhtml

Figure S1a. Mean duration (in days) of absence by cause of sickness

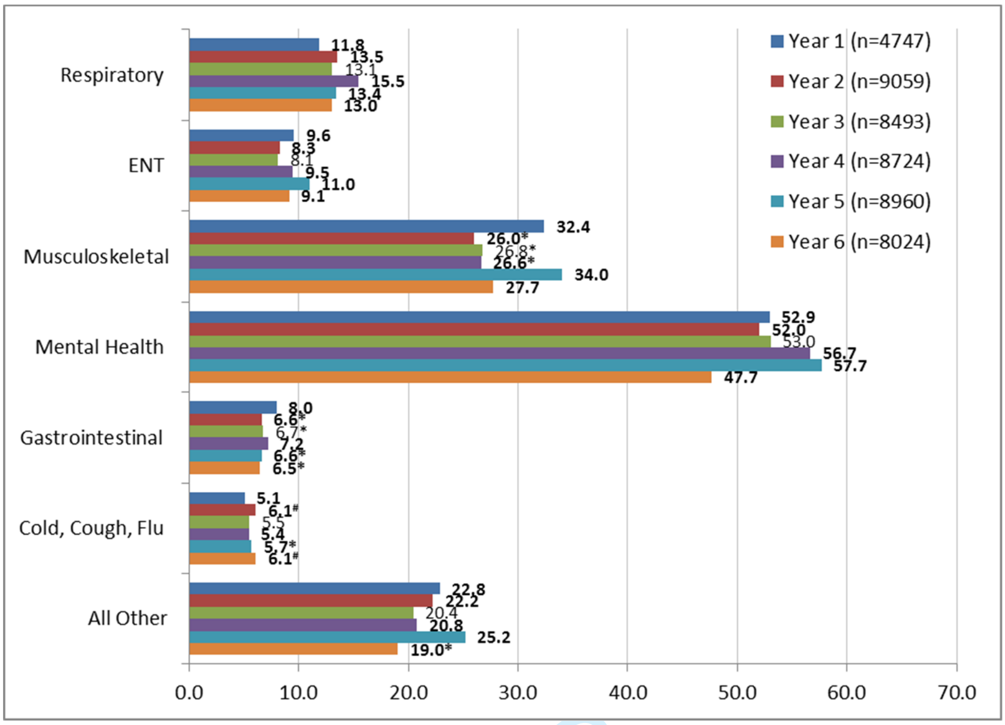

Figure S1b. Mean duration (in days) of absence by MSK cause of sickness

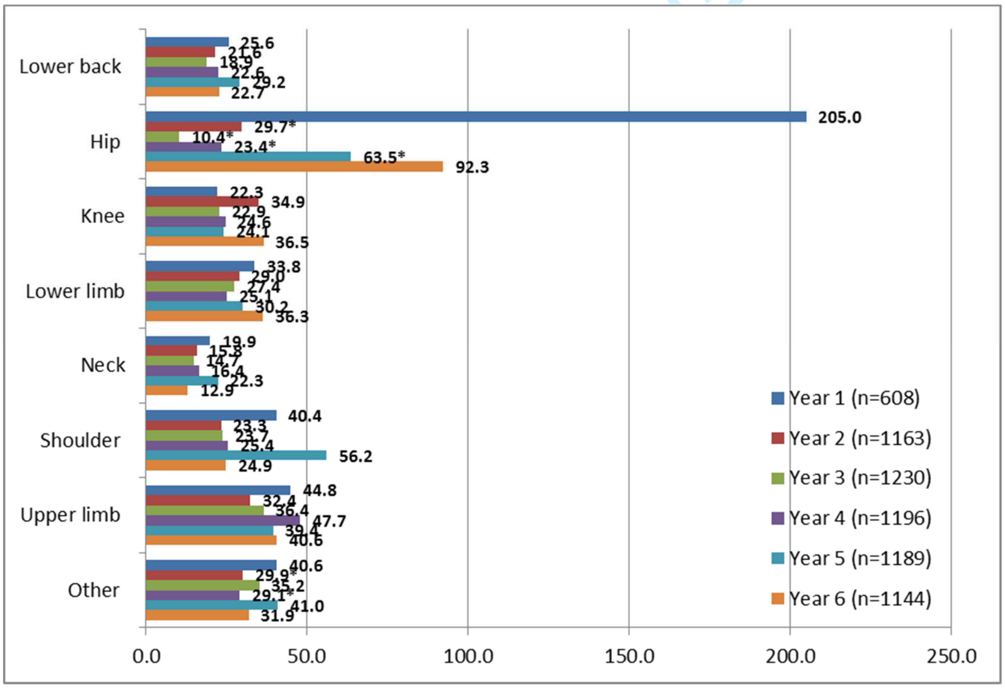

Figure S1c. Mean duration (in days) of absence by MH cause of sickness

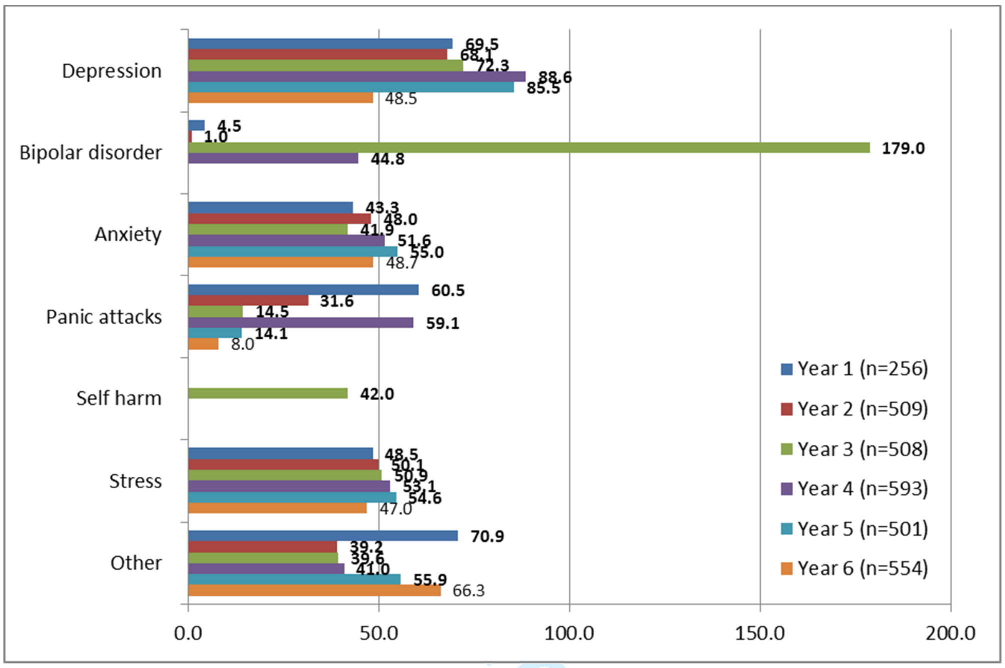

Table S1. Multivariate cox regression RTW hazard ratios for all (minus MSK and MH), MSK and MH conditions* with results from the Proportional Hazards test.

	All Conditions (minus MSK & MH)				Musculoskeletal Conditions				Mental Health Conditions			
	HR	95% CI	P	PH Test	HR	95% CI	P	PH Test	HR	95% CI	P	PH Test
**Population**												
All EASY except MSK & MH	1				-	-	-		-	-	-	
MSK	0.54	(0.53, 0.56)	0.000	0.0000	-	-	-		-	-	-	
MH	0.54	(0.33, 0.35)	0.000	0.0000	-	-	-		-	-	-	
**MSK condition**												
Lower back	-	-	-		1				-	-	-	
Knee	-	-	-		0.85	(0.75, 0.96)	0.008	0.0142	-	-	-	
Lower limb	-	-	-		0.80	(0.73, 0.88)	0.000	0.0005	-	-	-	
Neck	-	-	-		1.20	(1.08, 1.34)	0.001	0.5913	-	-	-	
Shoulder	-	-	-		0.80	(0.71, 0.91)	0.000	0.0013	-	-	-	
Upper limb	-	-	-		0.63	(0.59, 0.69)	0.000	0.0000	-	-	-	
Other	-	-	-		0.77	(0.72, 0.83)	0.000	0.0061	-	-	-	
**MH condition**												
Depression	-	-	-		-	-	-		1			
Anxiety	-	-	-		-	-	-		1.53	(1.33, 1.76)	0.000	0.2158
Stress	-	-	-		-	-	-		1.46	(1.30, 1.64)	0.000	0.0857
Other	-	-	-		-	-	-		1.51	(1.24, 1.85)	0.000	0.0004
**Gender**												
Male	1				1		1		1			
Female	0.91	(0.87, 0.95)	0.000	0.0000	0.81	(0.74, 0.88)	0.81	0.0004	1.06	(0.92, 1.23)	0.388	0.0702
**Age**	0.99	(0.99, 0.99)	0.000	0.0000	0.99	(0.99, 0.99)	0.99	0.8371	0.99	(0.99, 1.00)	0.000	0.4740
**Job category**												
Nursing/Midwifery	1				1				1			
Administrative services	1.19	(1.15, 1.23)	0.000	0.0059	1.18	(1.08, 1.28)	0.000	0.0025	0.97	(0.88, 1.08)	0.595	0.5969
Allied Health Profession	1.31	(1.26, 1.37)	0.000	0.0128	1.16	(1.05, 1.29)	0.004	0.3794	1.16	(1.00, 1.34)	0.043	0.7472
Healthcare Sciences	1.21	(1.14, 1.28)	0.000	0.3422	1.28	(1.09, 1.50)	0.003	0.0769	1.10	(0.87, 1.38)	0.437	0.3889

For peer review only - http://bmjopen.bmj.com/site/about/guidelines.xhtml

Manager	1.31	(1.04, 1.64)	0.020	0.0000	1.80	(1.04, 3.11)	0.036	0.7607	1.46	(0.79, 2.71)	0.229	0.7521
Medical & Dental	1.44	(1.33, 1.57)	0.000	0.0000	1.24	(0.97, 1.57)	0.080	0.0001	1.46	(0.90, 2.39)	0.127	0.0011
Medical and Dental Support	1.16	(1.06, 1.27)	0.001	0.4761	1.39	(1.06, 1.82)	0.018	0.9529	0.91	(0.62, 1.35)	0.644	0.7918
Other Therapeutic	1.17	(1.04, 1.31)	0.010	0.0017	1.44	(1.20, 1.75)	0.000	0.9414	1.22	(0.89, 1.67)	0.214	0.0001
Personal and Social Care	1.02	(0.97, 1.06)	0.458	0.7318	1.71	(1.28, 2.28)	0.000	0.8777	0.79	(0.51, 1.24)	0.308	0.7339
Support Services	1				1.10	(1.01, 1.19)	0.026	0.1622	1.21	(1.05, 1.40)	0.009	0.7192
**Job type**												
Part time	1				1				1			
Full time	1.12	(1.09, 1.14)	0.000	0.0003	1.04	(0.99, 1.11)	0.143	0.5504	1.09	(1.00, 1.18)	0.042	0.4597
**Year**												
May 08-Apr 09	1				1				1			
May 09-Apr 10	0.98	(0.95, 1.02)	0.417	0.0540	1.13	(1.02, 1.25)	0.020	0.9628	0.99	(0.85, 1.16)	0.91	0.9568
May 10-Apr 11	0.95	(0.92, 0.99)	0.013	0.0025	1.13	(1.03, 1.25)	0.014	0.7210	0.98	(0.84, 1.15)	0.822	0.2428
May 11-Apr 12	0.94	(0.90, 0.97)	0.001	0.0012	1.11	(1.00, 1.22)	0.045	0.6067	0.92	(0.79, 1.07)	0.279	0.5608
May 12-Apr 13	0.92	(0.88, 0.95)	0.000	0.0133	0.97	(0.87, 1.08)	0.560	0.3715	0.89	(0.77, 1.04)	0.154	0.3844
May 13-Apr 14	0.96	(0.92, 1.00)	0.029	0.0000	1.07	(0.96, 1.18)	0.217	0.3519	1.03	(0.88, 1.21)	0.687	0.9330

*results by Day of Absence and Season presented in Supplementary Material Tables S1a-f

For peer review only - http://bmjopen.bmj.com/site/about/guidelines.xhtml

Table S2a. Multivariate cox regression hazard ratios for full population, adjusted for sex, age, job category, job type, day of first absence, season of absence and year of absence.

All	HR	95% CI	P	PH Assumption
Day of absence				
Mon	1			
Tues	1.07	(1.04, 1.10)	0.000	0.5252
Wed	1.01	(0.98, 1.04)	0.384	0.3434
Thurs	1.01	(0.98, 1.04)	0.402	0.5309
Fri	0.96	(0.93, 0.99)	0.017	0.0021
Season				
Spring	1			
Summer	1.00	(0.97, 1.03)	0.931	0.0228
Autumn	1.00	(0.98, 1.03)	0.784	0.2458
Winter	1.02	(0.99, 1.05)	0.133	0.0905

Table S2b. Multivariate cox regression hazard ratios for full population, adjusted for sex, age, job category, job type, day of first absence, season of absence, year of absence and time varying coefficients.

All	HR	95% CI	P
Day of absence			
Mon	1		
Tues	1.06	(1.04, 1.09)	0.000
Wed	1.01	(0.98, 1.04)	0.612
Thurs	1.00	(0.97, 1.03)	0.976
Fri	0.94	(0.91, 0.98)	0.001
Season			
Spring	1		
Summer	1.00	(0.97, 1.03)	0.992
Autumn	1.01	(0.98, 1.03)	0.598
Winter	1.02	(0.99, 1.04)	0.163

For peer review only

For peer review only - http://bmjopen.bmj.com/site/about/guidelines.xhtml

Table S2c. Multivariate cox regression hazard ratios for staff with musculoskeletal conditions, adjusted for sex, age, job category, job type, day of first absence, season of absence and year of absence.

Musculoskeletal	HR	95% CI	P	PH Assumption
Day of absence				
Mon	1			
Tues	1.06	(0.99, 1.14)	0.105	0.2140
Wed	1.06	(0.99, 1.14)	0.1	0.5936
Thurs	1.08	(1.00, 1.17)	0.064	0.1939
Fri	1.00	(0.92, 1.09)	0.978	0.9834
Season				
Spring	1			
Summer	1.01	(0.94, 1.09)	0.694	0.7564
Autumn	1.01	(0.94, 1.08)	0.793	0.3528
Winter	1.02	(0.95, 1.09)	0.641	0.4783

Table S2d. Multivariate cox regression hazard ratios for staff with musculoskeletal conditions, adjusted for sex, age, job category, job type, day of first absence, season of absence, year of absence and time varying coefficients.

Musculoskeletal	HR	95% CI	P
Day of absence			
Mon	1		
Tues	1.06	(0.99, 1.14)	0.116
Wed	1.06	(0.99, 1.15)	0.090
Thurs	1.08	(1.00, 1.17)	0.050
Fri	1.00	(0.92, 1.09)	0.996
Season			
Spring	1		
Summer	1.01	(0.94, 1.08)	0.824
Autumn	1.01	(0.94, 1.08)	0.798
Winter	1.01	(0.95, 1.08)	0.679

For peer review only

Table S2e. Multivariate cox regression hazard ratios for staff with mental health conditions, adjusted for sex, age, job category, job type, day of first absence, season of absence and year of absence.

Mental health	HR	95% CI	P	PH Assumption
Job type				
Part time	1			
Full time	1.030726	(0.93, 1.15)	0.579	0.3336
Day of absence	1.015467	(0.92, 1.13)	0.772	0.027
Mon	0.987987	(0.88, 1.11)	0.839	0.5043
Tues	0.915464	(0.81, 1.03)	0.139	0.0068
Wed				
Thurs	1			
Fri	1.039512	(0.93, 1.16)	0.496	0.3026
Season	1.056732	(0.96, 1.17)	0.275	0.4850
Spring	1.019983	(0.92, 1.13)	0.712	0.5526
Summer	1			
Autumn	1.030726	(0.93, 1.15)	0.579	0.3336
Winter	1.015467	(0.92, 1.13)	0.772	0.027

Table S2f. Multivariate cox regression hazard ratios for staff with mental health conditions, adjusted for sex, age, job category, job type, day of first absence, season of absence, year of absence and time varying coefficients.

Mental health	HR	95% CI	P
Job type			
Part time	1		
Full time	1.00	(0.90, 1.12)	0.956
Day of absence	0.96	(0.86, 1.08)	0.519
Mon	0.91	(0.79, 1.05)	0.188
Tues	0.82	(0.70, 0.96)	0.011
Wed			
Thurs	1		
Fri	1.04	(0.93, 1.17)	0.448
Season	1.06	(0.96, .17)	0.235
Spring	1.03	(0.92, 1.14)	0.638
Summer	1		
Autumn	1.00	(0.90, 1.12)	0.956
Winter	0.96	(0.86, 1.08)	0.519

For peer review only - http://bmjopen.bmj.com/site/about/guidelines.xhtml

For peer review only

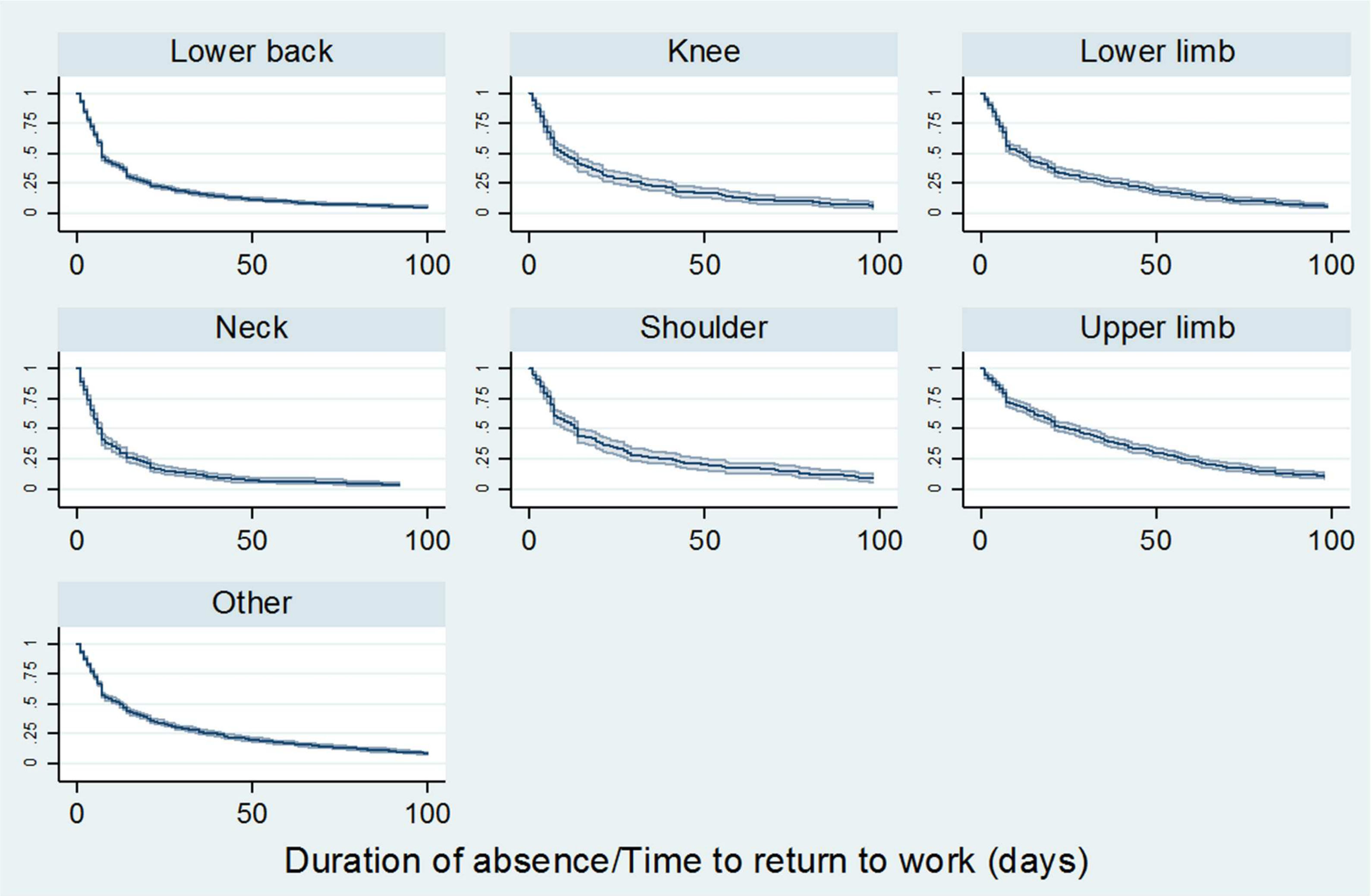

Figure S2. Return to Work curves for all MSK-related absences by MSK condition with 95%CI

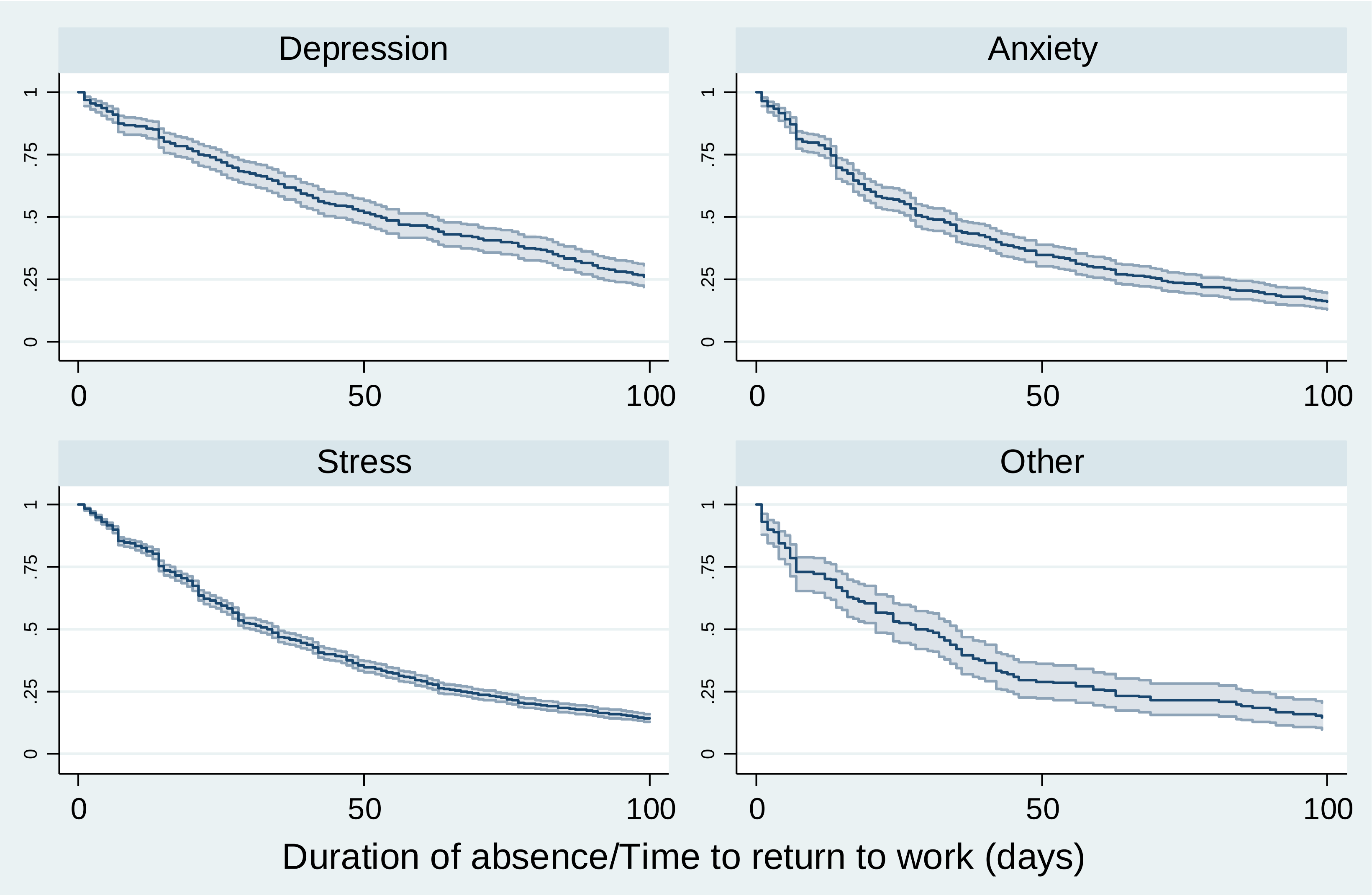

Figure S3. Return to Work curves for all MH-related absences by MH condition with 95%CI

For peer review only - http://bmjopen.bmj.com/site/about/guidelines.xhtml

STROBE Statement—checklist of items that should be included in reports of observational studies

	Item No.	Recommendation	Page No.	Relevant text from manuscript
**Title and abstract**	1	(*a*) Indicate the study's design with a commonly used term in the title or the abstract	2	A retrospective cohort study of Scottish Healthcare Workers.
		(*b*) Provide in the abstract an informative and balanced summary of what was done and what was found	2	Survival analyses and Cox's proportional hazards models were used to estimate SA duration……
**Introduction**				
Background/rationale	2	Explain the scientific background and rationale for the investigation being reported	4-7	
Objectives	3	State specific objectives, including any prespecified hypotheses	7	Study aim
**Methods**				
Study design	4	Present key elements of study design early in the paper	8	
Setting	5	Describe the setting, locations, and relevant dates, including periods of recruitment, exposure, follow-up, and data collection	8-9	
Participants	6	(*a*) *Cohort study*—Give the eligibility criteria, and the sources and methods of selection of participants. Describe methods of follow-up<br>*Case-control study*—Give the eligibility criteria, and the sources and methods of case ascertainment and control selection. Give the rationale for the choice of cases and controls<br>*Cross-sectional study*—Give the eligibility criteria, and the sources and methods of selection of participants	8-10	
		(*b*) *Cohort study*—For matched studies, give matching criteria and number of exposed and unexposed<br>*Case-control study*—For matched studies, give matching criteria and the number of controls per case		
Variables	7	Clearly define all outcomes, exposures, predictors, potential confounders, and effect modifiers. Give diagnostic criteria, if applicable	9-10	
Data sources/ measurement	8*	For each variable of interest, give sources of data and details of methods of assessment (measurement). Describe comparability of assessment methods if there is more than one group	*9-10*	
Bias	9	Describe any efforts to address potential sources of bias	9-10	multivariate model controlled for several occupational and individual
Study size	10	Explain how the study size was arrived at	8-9	

Continued on next page

For peer review only - http://bmjopen.bmj.com/site/about/guidelines.xhtml

Quantitative variables	11	Explain how quantitative variables were handled in the analyses. If applicable, describe which groupings were chosen and why	9-10	
Statistical methods	12	(*a*) Describe all statistical methods, including those used to control for confounding	9-10	
		(*b*) Describe any methods used to examine subgroups and interactions	8-10	
		(*c*) Explain how missing data were addressed	8-9	
		(*d*) *Cohort study*—If applicable, explain how loss to follow-up was addressed<br>*Case-control study*—If applicable, explain how matching of cases and controls was addressed<br>*Cross-sectional study*—If applicable, describe analytical methods taking account of sampling strategy	9	
		(*e*) Describe any sensitivity analyses		
**Results**				
Participants	13*	(a) Report numbers of individuals at each stage of study—eg numbers potentially eligible, examined for eligibility, confirmed eligible, included in the study, completing follow-up, and analysed	10-11	
		(b) Give reasons for non-participation at each stage	na	
		(c) Consider use of a flow diagram	na	
Descriptive data	14*	(a) Give characteristics of study participants (eg demographic, clinical, social) and information on exposures and potential confounders	11	Table 1
		(b) Indicate number of participants with missing data for each variable of interest	na	
		(c) *Cohort study*—Summarise follow-up time (eg, average and total amount)		
Outcome data	15*	*Cohort study*—Report numbers of outcome events or summary measures over time		
		Case-control study—Report numbers in each exposure category, or summary measures of exposure		
		Cross-sectional study—Report numbers of outcome events or summary measures	*11& 14*	*Tables 1 &2*
Main results	16	(*a*) Give unadjusted estimates and, if applicable, confounder-adjusted estimates and their precision (eg, 95% confidence interval). Make clear which confounders were adjusted for and why they were included	14 & 16	
		(*b*) Report category boundaries when continuous variables were categorized		
		(*c*) If relevant, consider translating estimates of relative risk into absolute risk for a meaningful time period		

Continued on next page

For peer review only - http://bmjopen.bmj.com/site/about/guidelines.xhtml

Other analyses	17	Report other analyses done—eg analyses of subgroups and interactions, and sensitivity analyses	17	The analysis by year of absence…
**Discussion**				
Key results	18	Summarise key results with reference to study objectives	17-18	Summary of findings section
Limitations	19	Discuss limitations of the study, taking into account sources of potential bias or imprecision. Discuss both direction and magnitude of any potential bias	20	Study limitations section
Interpretation	20	Give a cautious overall interpretation of results considering objectives, limitations, multiplicity of analyses, results from similar studies, and other relevant evidence	17-20	
Generalisability	21	Discuss the generalisability (external validity) of the study results	20	The latest data show that the NHSL SA rate is in line with the Scottish NHS average
**Other information**				
Funding	22	Give the source of funding and the role of the funders for the present study and, if applicable, for the original study on which the present article is based	22	Funding section

*Give information separately for cases and controls in case-control studies and, if applicable, for exposed and unexposed groups in cohort and cross-sectional studies.

Note: An Explanation and Elaboration article discusses each checklist item and gives methodological background and published examples of transparent reporting. The STROBE checklist is best used in conjunction with this article (freely available on the Web sites of PLoS Medicine at http://www.plosmedicine.org/, Annals of Internal Medicine at http://www.annals.org/, and Epidemiology at http://www.epidem.com/). Information on the STROBE Initiative is available at www.strobe-statement.org.

For peer review only

For peer review only - http://bmjopen.bmj.com/site/about/guidelines.xhtml
